# *CCDC12* promotes tumor development and invasion through the Snail pathway in colon adenocarcinoma

**DOI:** 10.1038/s41419-022-04617-y

**Published:** 2022-02-25

**Authors:** Fengying Du, Lipan Peng, Qiang Wang, Kangdi Dong, Wenting Pei, Hongqing Zhuo, Tao Xu, Changqing Jing, Leping Li, Jizhun Zhang

**Affiliations:** 1 Department of Gastrointestinal Surgery, Shandong Provincial Hospital Affiliated to Shandong First Medical University, Jinan, Shandong China; 2 Cheeloo College of Medicine, Shandong University, Jinan, Shandong China; 3 Personnel Office, Shandong Provincial Hospital Affiliated to Shandong First Medical University, Jinan, Shandong China; 4 Hematology and Oncology, Qilu Children’s Hospital of Shandong University, Jinan, Shandong China; 5 Center for Post-doctoral Studies, Shandong University of Traditional Chinese Medicine, Jinan, Shandong China

**Keywords:** Diagnostic markers, Colon cancer

## Abstract

Integrative expression Quantitative Trait Loci (eQTL) analysis found that rs8180040 was significantly associated with Coiled-coil domain containing 12 (*CCDC12*) in colon adenocarcinoma (COAD) patients. Immunohistochemical staining and western blotting confirmed *CCDC12* was highly expressed in COAD tissues, which was consistent with RNA-Seq data from the TCGA database. Knockdown of *CCDC12* could significantly reduce proliferation, migration, invasion, and tumorigenicity of colon cancer cells, while exogenous overexpression of *CCDC12* had the opposite effect. Four plex Isobaric Tags for Relative and Absolute Quantitation assays were performed to determine its function and potential regulatory mechanism and demonstrated that overexpression of *CCDC12* would change proteins on the adherens junction pathway. Overexpressed Snail and knocked down *CCDC12* subsequently in SW480 cells, and we found that overexpression of Snail did not significantly change *CCDC12* levels in SW480 cells, while knockdown of *CCDC12* reduced that of Snail. *CCDC12* plays a significant role in tumorigenesis, development, and invasion of COAD and may affect the epithelial to mesenchymal transformation process of colon cancer cells by regulating the Snail pathway.

## Introduction

The incidence of colon cancer is the fifth-highest for malignant tumors, with over one million new colon cancer patients worldwide each year [1]. In the Asian population, approximately 90% of colon cancers are histologically classified as adenocarcinomas, and the prognosis of them is poor [2, 3]. Refractory and metastatic colon adenocarcinoma (COAD) has been a major problem worldwide [4–6]. Somatic mutations and activation of key oncogenic pathways have often been observed in COAD. Hence, it is essential to understand the mechanism of refractory and metastatic COAD.

The coiled-coil domain containing 12 (*CCDC12*) is an evolutionarily conserved protein that encodes a coiled-coil domain, which is located in the 3p21.31 region of human chromosome 3 [7]. *CCDC12* has been reported to be associated with erythroid differentiation [8], and split-ubiquitin system [9]. However, the coiled-coil domain-containing family members have been associated with cancer. *CCDC106* is related to the progression and poor prognosis of non-small cell lung cancer [10], and *CCDC67* has been demonstrated to inhibit the proliferation of papillary thyroid carcinomas [11]. A genome-wide association study (GWAS) identified colorectal cancer risk single nucleotide polymorphism (SNP) rs1076394 as an expression Quantitative Trait Loci (eQTL) for *CCDC12* [12]. However, the specific carcinogenesis of *CCDC12* has not been deciphered.

In this study, we demonstrated that high expression levels of *CCDC12* in COAD were closely associated with tumor development and aggression. *CCDC12* promoted COAD tumor cell proliferation, invasion, migration, and inhibited apoptosis in in vitro and in vivo experiments. Furthermore, *CCDC12* could regulate epithelial to mesenchymal transformation (EMT) of COAD cells through the Snail pathway. Our study demonstrated a biological link between *CCDC12* and COAD, which could be used as a potential therapeutic target.

## Results

### SNP rs8180040 is significantly associated with *CCDC12* expression based on integrative eQTL analysis

**Fig. 1:**
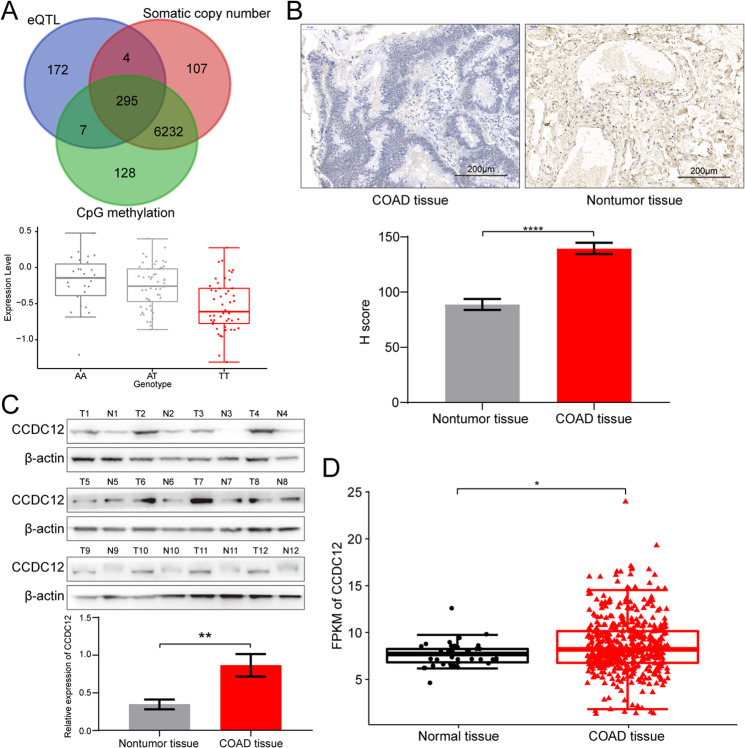
CCDC12 is an oncogene associated with colon cancer. **A** The cis-eQTL analysis determined a significant association between SNP rs8180040 and CCDC12 expression levels. **B** IHC staining demonstrating higher expression levels of CCDC12 in COAD tissues (40×). **C** Western blotting demonstrates high expression levels of CCDC12 in COAD tissues. **D** The expression status of CCDC12 derived from COAD RNA-Seq data from the TCGA database. (* <0.05; ** <0.01; **** <0.0001).

In ancestry verification, the 130 samples were compared with Haplotypemap (The International HapMap Project, HapMap, Han Chinese in Beijing, CHB), and their clinicopathologic data were summarized in Table S1. After filtering out false discovery rate (FDR) < 0.1, we obtained 5,029,762 SNP-genes and identified 2,030 significant associations with. They mapped to a total of 1964 SNP loci and 478 unique target genes (Table S2), where 332 were found to be regulated by a single cis-acting SNP locus, and 1,899 cis-acting eQTL loci were associated with one target gene. In addition, 299 target genes were significantly associated with somatic copy number, and 302 were also target genes of eQTLs (Fig. 1A). Subsequently, we identified 25 SNP-gene expression associations in 22 loci with *P* < 0.05 for genes within 2 Mb of the risk SNPs (Table S3). After correcting for multiple testing (FDR < 0.05), only one SNP-gene association was found to be significant (SNP rs8180040 with genes *CCDC12*, genotype in Fig. 1A).

### High expression of *CCDC12* in COAD shows its pro-cancer properties

Immunohistochemistry showed that CCDC12 was overexpressed in COAD specimens in contrast with normal colon tissues (51/75 vs. 8/75, *P* < 0.001, Fig. 1B), which is consistent with our previous study [13]. Subsequently, western blotting with fresh tissues from 12 patients also confirmed this result. CCDC12 was significantly higher in 10 colon cancer tissue samples compared to normal (Fig. 1C). RNA-Seq data from the TCGA database, which included 456 COAD patients and 41 corresponding normal tissues, showed that *CCDC12* was statistically significantly overexpressed in COAD (*P* < 0.001, Fig. 1D). All these results imply that *CCDC12* is a colon cancer-associated oncogene.

### Knockdown of *CCDC12* inhibits tumor proliferation, migration, invasion, and promoted apoptosis

Through real-time quantitative polymerase chain reaction (RT-qPCR) with 5 different colon cancer cell lines and the CCD-18Co as the control, we found *CCDC12* was relatively overexpressed in LOVO and SW480 cell lines (Fig. S1), hence we reduced their expression levels using sh-CCDC12 RNA. And shRNA1 had the best knockdown efficiency for *CCDC12* in both SW480 and LOVO cell lines (Fig. S2), which were used in subsequent in vitro and in vivo assays. We set *CCDC12* knockdown group (CCDC12-KD, cells transfected with shRNA1), normal group (NC, naïve cells), and blank control group (Vec, cells transfected with empty vector, Fig. S3A), and each experiment was repeated three times independently.

**Fig. 2:**
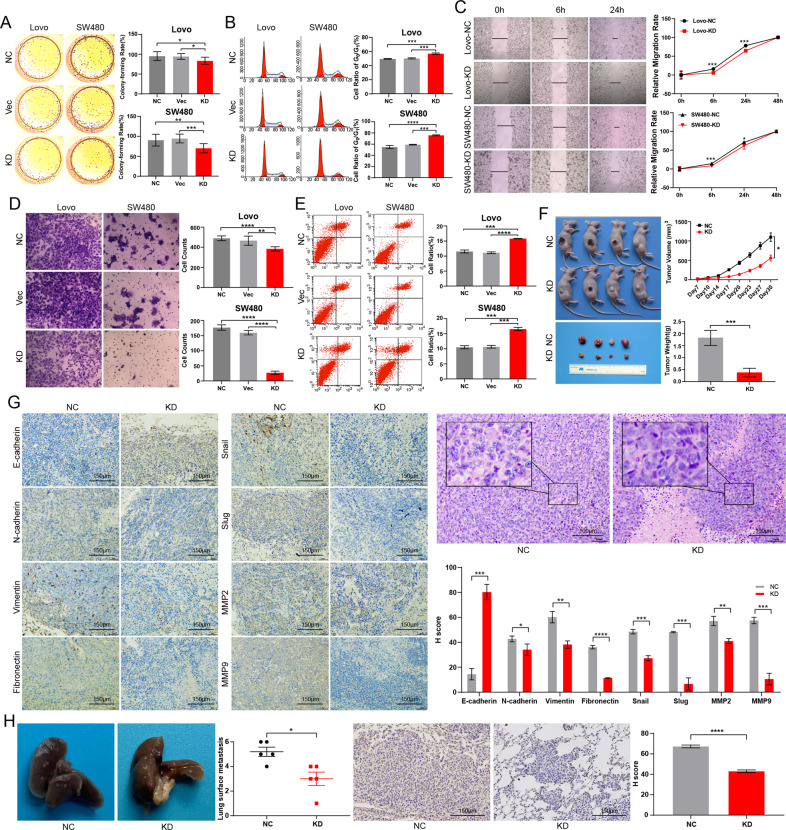
Knock-down of CCDC12 expression levels inhibited the biological behavior of colon cancer cells effectively. **A** Inhibition of colony formation in colon cancer cells (100×). **B** The ratio of cells blocked in the *G*_0_/*G*_1_ phase increased. **C** Decrease in the distance of cell migration (200×). **D** Reduced invasion of cells (200×). **E** Apoptosis rates of cells were elevated. **F** The volumes and weights of xenograft tumors were significantly reduced. **G** IHC and H&E stain for the expression of key EMT molecules in xenograft tumor (40×). **H** Liver metastatic nodules with their IHC staining (40×). (* <0.05; ** <0.01; *** <0.001; **** <0.0001).

After exogenous silencing of *CCDC12*, the colony-forming ability of cells was significantly reduced (Fig. 2A), and predominately in the *G*_0_/*G*_1_ phase (Fig. 2B). In MTS assays, the rates of cell proliferation in the CCDC12-KD group were significantly lower (Fig. S4A). Wound-healing assays proved cell migration downregulated, especially at 24 hs (Fig. 2C). And we found that the cell invasion ability was much lower after *CCDC12* knockdown, which more obvious in the SW480 cell line (Fig. 2D). With the Annexin V-FITC kit, we observed that the total apoptosis rate (UR + LR) of cells in the CCDC12-KD group was increased (Fig. 2E).

After 30 days of tumor-bearing growth, the volume and weight of tumors in BABL/c mice from xenograft models in the CCDC12-KD group were much smaller (Fig. 2F, with one mouse died due to an accident). The immunohistochemistry (IHC) stain results of transplanted tumors confirmed that many classical molecular markers of EMT changed significantly (Fig. 2G). Hematoxylin-eosin (H&E) stain showed the cell nucleus division was reduced, the cell gap became smaller, and the cells were relatively epithelialized. After the knockdown of *CCDC12*, the number of cancer nodules on the liver of mice was reduced, and IHC showed that the expression of CCDC12 was also downregulated (Fig. 2H). All these results indicated that knockdown of *CCDC12* could effectively inhibit colon cancer cell proliferation, invasion, migration, and promote apoptosis in vivo and in vitro, which was related to EMT.

### Overexpression of *CCDC12* promotes tumor proliferation, migration, invasion, and inhibits apoptosis

**Fig. 3:**
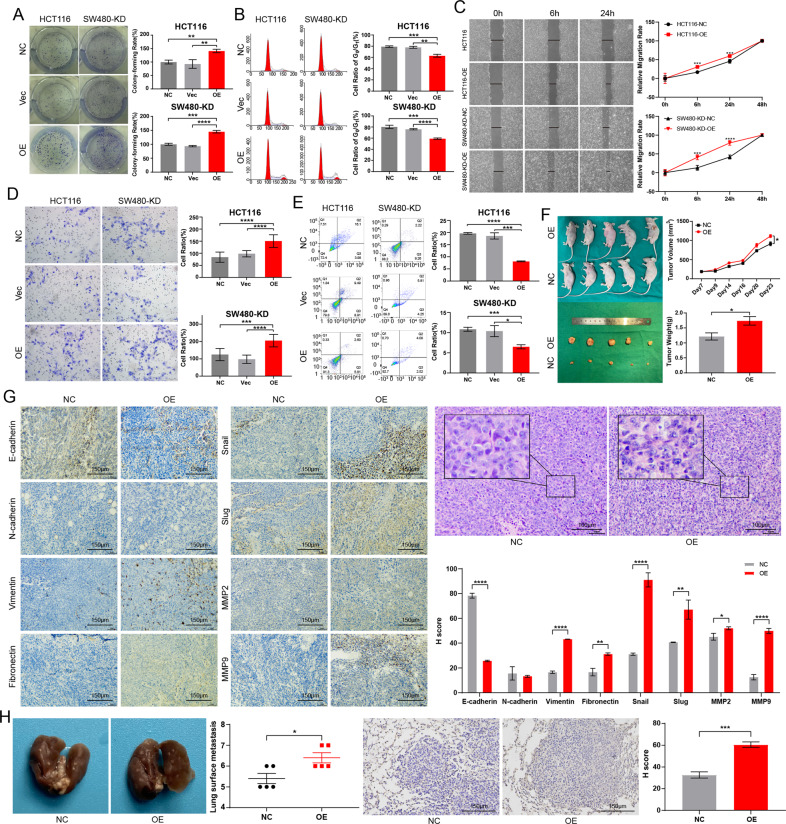
Overexpression CCDC12 enhances the biological behavior of tumor cells. **A** Increase in colony formation in colon cancer cells (100×). **B** The proportion of cells blocked in the *G*_0_/*G*_1_ phase was reduced. **C** Increase in cell migration ability (200×). **D** Increase in invasion ability (200×). **E** Apoptosis rates of cells were reduced. **F** The volumes and weights of xenograft tumors were significantly increased. **G** IHC and H&E stain for the expression of key EMT molecules in xenograft tumor (40×). **H** Liver metastatic nodules with their IHC staining (40×). (* <0.05; ** <0.01; *** <0.001; **** <0.0001).

Based on expression levels of *CCDC12* in colon cancer cell lines (Fig. S1), HCT116 cell line and *CCDC12*-knocked SW480 cell line were selected to overexpress *CCDC12* (OE-CCDC12 group, Figs. S5 and S3B), with NC group and Vec group as a knockdown. On the seventh day, both SW480-KD and HCT116 cells showed significantly better clone-formation ability after overexpression of *CCDC12* (Fig. 3A), and the proportion of cells in the G_0_/G_1_ phase decreased (Fig. 3B). In MTS assays, the cells did proliferate more in the OE-CCDC12 group (Fig. S4B). Next, we found cells exhibited the opposite result to those after knockdown of *CCDC12*, with a greatly enhanced migration ability (Fig. 3C). As shown in Fig. 3D, the invasive ability of cells in the OE-CCDC12 group was higher compared to cells in NC and Vec groups. Furthermore, the total apoptotic rate of cells was reduced by approximately 50% in the OE-CCDC12 group (Fig. 3E), which was more evident in HCT116 cell lines.

Tumor volume and weight from nude mice injected with OE-CCDC12 HCT116 cells were larger compared with native HCT116 cells (Fig. 3F). Unlike knockdown of *CCDC12*, the results of IHC didn’t show changes on N-cadherin when overexpressed *CCDC12*, while others changed (Fig. 3G). H&E staining did confirm increased nuclear division and more disordered after overexpression of *CCDC12*. As for the number of nodules in the liver, it did increase and IHC confirmed the expression of CCDC12 also increased (Fig. 3H). What is interesting is that *CCDC12* overexpressed caused cells in the liver of mice to become tightly connected, which also echoed the performance after the knockdown of *CCDC12*. These findings demonstrate that over-expression of *CCDC12* increases colon cancer cell proliferation, migration, and invasiveness both in vivo and in vitro while reducing apoptosis levels and promoting the development of cells towards the mesenchymal state.

### *CCDC12* induces an epithelial-mesenchymal transition to aggravate COAD

**Fig. 4:**
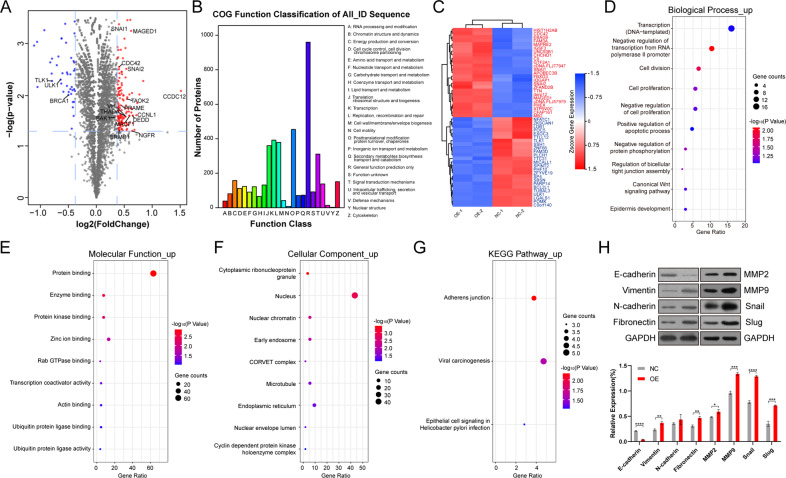
Bioinformatic analysis and validation of differentially expressed proteins. **A** Differentially expressed proteins after CCDC12 overexpressed. **B** COG function annotation of all differentially expressed proteins. **C** Cluster heatmap of the top 50 differentially expressed proteins. **D** GO annotation of the increased expressed proteins in the Biological Process term (BP). **E** GO annotation of the increased expressed proteins in Molecular Function term (MF). **F** GO annotation of the increased expressed proteins in Cellular Component (CC). **G** KEGG pathway enrichment results for the increased expressed proteins. **H** Western blotting to verify the association between CCDC12 and key EMT-related proteins. (* <0.05; ** <0.01; *** <0.001; **** <0.0001).

We compared HCT116 cells after overexpression of *CCDC12* with native HCT116 cells using 4 plex isobaric tags for relative and absolute quantitation (iTRAQ) technology to discover the specific effects of CCDC12 on the cells at the protein level. We got 127 upregulated proteins and 42 downregulated proteins in OE-CCDC12 HCT116 cells (Fig. 4A). According to the Orthologous Groups of proteins (COG) database, differentially expressed proteins enriched in translation, ribosomal structure, and biogenesis; posttranslational modification, protein turnover, chaperones; signal transduction mechanisms (Fig. 4B). These annotated functions imply that CCDC12 plays a signaling role in the nucleus, especially in the mis-signaling process of cancer. The top 50 differentially expressed proteins clustered into several groups to classify *CCDC12* interacting proteins (Fig. 4C, full heatmap shown in Fig. S6, and the detailed data sheet is shown in Table S4). Within this, EMT-related markers *SNAI1*, *SNAI2*, and *CDC42* were upregulated along with *CCDC12*. We then annotated the increased and decreased proteins in Gene Ontology (GO) analysis separately, and only increased proteins were mentioned in this study (remaining results presented in Fig. S7). As for Biological Process (BP) terms, proteins were mostly annotated into transcription (DNA-templated), cell division, and cell proliferation (Fig. 4D); to Molecular Function (MF), the majority were protein binding, enzyme binding, protein kinase binding, and Zinc ion binding (Fig. 4E); in Cellular Component (CC), they were cytoplasmic ribonucleoprotein granule and nucleus (Fig. 4F). In terms of Kyoto Encyclopedia of Genes and Genomes (KEGG) signaling pathways, adherens junction was annotated (Fig. 4G), which reinforces the potential association between *CCDC12* and EMT. To test this conjecture, we performed western blotting using the same cells. As shown in Fig. 4H, overexpression of *CCDC12* induced significant changes in E-cadherin, Vimentin, Fibronectin, Matrix Metallopeptidase 9 (MMP 9), Snail, and Slug. Encouragingly, Snail and Slug, two members of the Snail superfamily of zinc-finger transcription factors, produced great upregulation, which is in line with the results of the iTRAQ experiment. In summary, *CCDC12* regulated COAD by altering the expression levels of several biologically functional proteins that were associated with EMT, especially zinc finger transcription factors.

### *CCDC12* regulates EMT in COAD via zinc finger transcription factors

**Fig. 5:**
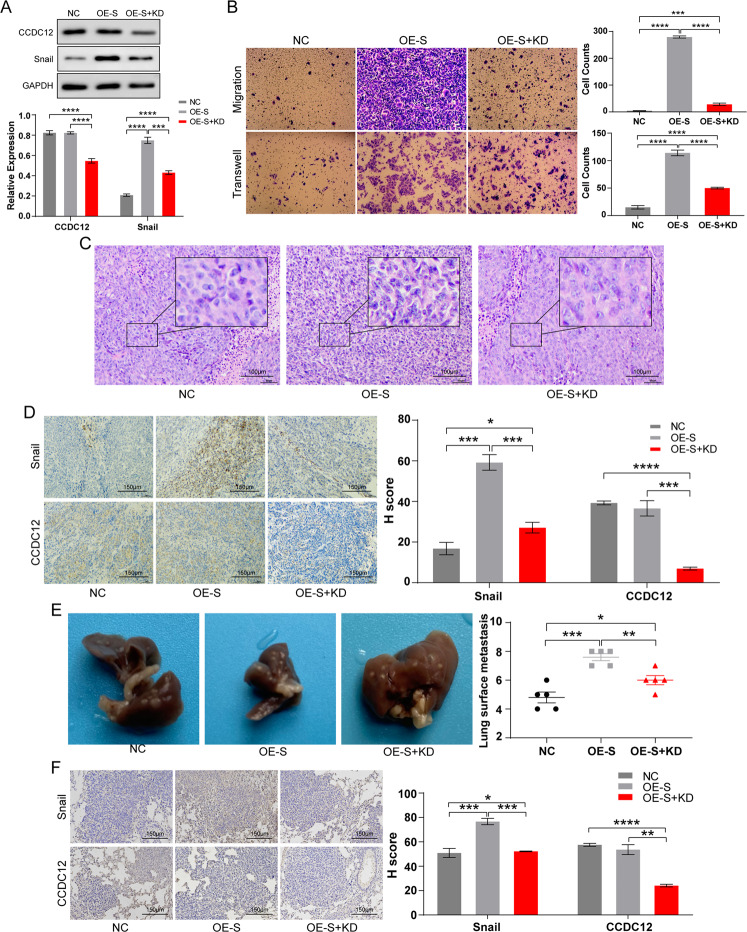
CCDC12 regulates colon cancer progression through Snail. **A** Western blot confirmed CCDC12 affected the expression levels of Snail. **B** CCDC12 regulates Snail to promote invasion and migration of colon cancer cells (200×). **C** H&E stain evaluates the malignancy of xenograft tumor (40×). **D** IHC stain for the expression of CCDC12 and Snail in xenograft tumor (40×). **E** Number of liver nodules after overexpression of Snail and sequential knockdown CCDC12. **F** IHC stain for liver nodules (40×). (* <0.05; ** <0.01; *** <0.001; **** <0.0001).

We overexpressed Snail in SW480 cells (Fig. S8) and then used shRNA1 to knockdown *CCDC12* sequentially. The results of western blotting confirmed that CCDC12 did not change after overexpression of Snail, but knockdown of *CCDC12* reduced the amount of Snail to a certain extent (Fig. 5A). To determine whether the association between *CCDC12* and Snail affects the process of EMT, a transwell assay was performed to assess changes in cell invasion and migration. And we found overexpressed Snail significantly increased cell invasion and migration, while, knocking down *CCDC12* subsequently reduced them but were still higher compared to cells in the NC group (Fig. 5B). After injecting cells into mice, the OE-Snail group showed significantly increased nuclear division and decreased intercellular adhesion, which was improved after the knockdown of *CCDC12* (Fig. 5C). The IHC results of transplanted tumors were similar to WB, sequential knockdown of *CCDC12* did reduce the level of Snail as well, but it was still higher than that from the NC group (Fig. 5D). On metastatic hepatic nodules, Snail greatly increased the number of nodules, and subsequently knocked down *CCDC12* also reduced it to some extent (Fig. 5E). As shown in Fig. 5F, the same results were also presented on the IHC of metastatic hepatic nodules, but the degree of dispersion between cells did not seem to change much. These results suggested that *CCDC12* regulates EMT in COAD by affecting Snail expression.

## Discussion

The *CCDC12* gene, located on 3p21.31 of chromosome 3 [7], has been reported to play a role in colorectal tumorigenesis [12]. *CCDC12* has been demonstrated to accelerate the growth of K562 cells by upregulating CD235, ε-globin, and γ-globin in human chronic myeloid leukemia [8]. Anne et al. reported that *CCDC12* may be associated with ubiquitination [9], which is particularly critical in tumor cells and could participate in the modification and degradation of some cancer factors to affect the biological behavior of tumors. In addition, Ke et al. using GWAS found that *CCDC12* may be a potential risk gene for colorectal cancer and associated with a potential regulatory variant, rs1076394 [12, 14]. In this study, we demonstrated that *CCDC12* was highly expressed in colon adenocarcinomas and may affect cancer metastasis by regulating EMT. Meanwhile, *CCDC12* promotes EMT in COAD through the Snail pathway, which has not been studied yet.

We analyzed GWAS data from National Human Genome Research Institute (NHGRI) database and then performed deep mining of colorectal cancer-related data from TCGA. Our team found numerous colorectal cancer-related risk SNPs, which were verified through 130 additional samples. With this, we identified rs10505477 and rs6983267 that had high pairwise linkage disequilibrium. After integrative eQTL-based analysis, we identified 25 SNP-gene expression associations in 22 risk SNP loci, and multiple corrections were performed to improve the reliability. We found SNP rs8180040 and *CCDC12* had a significant correlation, both of which were located on chromosome 3 and had a strong geometric correlation. SNP rs8180040 was located at chr3: 47347457 (GRCh38.p12) with *T* > *A* allele (*T* = 0.5992 and *A* = 0.4008). The gene frequency of rs8180040 was slightly different in different populations. The Asian population had the highest allele A frequency of 0.5, especially in East Asian populations; the African population had the lowest frequency of 0.23; the European population was close to the average (*A* = 0.4088) [12, 15, 16], and our results were similar to previous studies. Hence, we inferred that rs8180040 may affect the occurrence of colorectal cancer by regulating *CCDC12* expression.

In our study, we proved that *CCDC12* was highly expressed in colon cancer tissues by RNA sequencing data from the TCGA database, western blotting of fresh tissues, and IHC of paraffin-embedded tissues. These results demonstrate that *CCDC12* is a proto-oncogene from multiple perspectives, which is consistent with the previous reports [12]. Cox regression analysis showed that *CCDC12* was not a prognostic factor, although the survival curves could be better separated, this was not statistically significant. We suspect that it may be caused by an excess of mutation in *CCDC12* in COAD patients from the TCGA database as if *TP53* is highly expressed in prostate cancer. Interestingly, in female patients, we found *CCDC12* overexpression leads to a poorer prognosis, but not in male patients. These findings demonstrated that *CCDC12* plays a potential role in COAD tumorigenesis and development.

We next performed a series of in vivo and in vitro experiments to determine how *CCDC12* regulates the EMT process of COAD cells to understand its biological role. In in vivo experiments, both knockdown and overexpression of *CCDC12* resulted in tumor cells exhibiting a clear transition between epithelial and mesenchymal states, and overexpression of *CCDC12* effectively promoted differentiation of colon cancer cells to the mesenchymal state. This was also well verified in vitro experiments. In particular, the results of H&E staining and IHC of tumors, and IHC of metastatic hepatic nodules from nude mouse xenograft tumor model reinforced that *CCDC12* could accelerate nuclear division of cells, weaken intercellular adhesion and increase distance, and also downregulate the expression of E-cadherin, a typical epithelial state marker, and upregulate Vimentin, Fibronectin, Snail, Slug, MMP 2, and MMP 9, classical markers of mesenchymal state [17, 18]. More confusingly, overexpression and knockdown of *CCDC12* did not occur to the same extent but had similar effects on EMT markers, a phenomenon that was particularly evident in IHC staining of nude mouse transplanted tumors. This may be a spurious change caused by the species differences between human-derived cells and BABL/c nude mice. Coupled with the fact that our modified *CCDC12* is a human-derived sequence, it is unknown whether this will lead to changes in the gene interaction network of tumorigenesis in nude mice. For this reason, we are now carrying out a study on humanized nude mice to observe the regulatory role of human *CCDC12* in EMT. In addition, the knockdown of *CCDC12* resulted in a higher proportion of cells in the *G*_0_/*G*_1_ phase, which is the exact opposite of what happened after overexpression of *CCDC12*. The blockage of the cell cycle may be related to the failure of the cell cycle checkpoint, which is one of the underlying causes of tumorigenesis [19–21]. To our delight, the results of flow cytometry showed that *CCDC12* could effectively promote apoptosis of cancer cells after knockdown, and correction of cancer cell non-apoptosis is one of the means to treat cancer [22–24]. Our team ventured the hypothesis that a complete knockout of *CCDC12* might hold promise for treating colon adenocarcinoma, but perhaps it would also cause other dysfunctions as a result of a complete knockout. After all, *CCDC12* plays more than just a role in the human body.

Through iTRAQ assays, we observed that overexpression of *CCDC12* could induce expression levels changes in 169 proteins, which clarifies, on a smaller scale, the molecules that interact with *CCDC12*. In COG analysis, about one thousand proteins are annotated with definite functions and enriched in the following annotations: translation, ribosomal structure, and biogenesis [25]; transcription [26]; and signal transduction mechanisms [27], all of which have been strongly associated with cancer. These functions were annotated to suggest a possible role of *CCDC12* as a regulator in colorectal cancer. Because of its localization in the nucleus, it may influence cell division by regulating ribosome production, a process that involves elaborate gene interactions and signal transduction. Cluster analysis divided the differentially expressed proteins into two groups and was in good agreement with the original groups. For BP classification in GO annotation, the majority of proteins were annotated to transcription (DNA-templated), cell division, and cell proliferation. These constituted the core functions involved in cancer biology, which were based on changes in protein-coding genes and non-coding regulatory elements [28], and this is also consistent with the results of the phenotypic experiments we conducted, in which *CCDC12* promotes the proliferation of colon cancer cells. We also classified differentially expressed proteins into MF terms, which included zinc ion binding. Zinc ions play a key role in homeostasis, immune function, and apoptosis [29], and may also induce p53 misfolding [30]. We suspected that the zinc ion mentioned in the results might be a member of the Snail superfamily and therefore performed a follow-up verification, which indeed turned out to be true, that *CCDC12* influences the malignant progression of colon cancer through Snail. With regards to CC classification, several proteins were annotated to the nucleus, cytoplasmic ribonucleoprotein granule, and nuclear chromatin. And we observed that *CCDC12* was localized in the nucleoplasm, which suggested that *CCDC12* may affect proteins in the nucleus, such as Snail (localized in the nucleus and cytosol). KEGG pathway enrichment analysis demonstrated that cancer-related pathways such as adherens junction and viral carcinogenesis were annotated. Early EMT is associated with the overall deterioration of cell-cell adhesion, which triggers the front-rear polarization of cells required for migration [17]. It is gratifying that in the adherens junction pathway, Snail and Slug are its typical representatives, both of which could be detected in the nucleus. All these findings demonstrated that *CCDC12* may regulate EMT of colon cancer cells through Snail located in the nucleus.

During EMT, epithelial cells lose their cell polarity and connection with the basement membrane, resulting in the ability to migrate and invade, resist apoptosis, and degrade extracellular matrix [18], which were consistent with our in vitro experiments. And in in vivo experiments, *CCDC12* has also been shown to promote transplant tumor growth and liver metastasis, with sections of tumors and liver nodules indeed showing a higher degree of cell malignancy. *CCDC12* is an oncogene and is associated with the EMT process, which is not previously reported. Fortunately, Snail-associated animal models also showed the correlation between *CCDC12* and Snail, and precisely, *CCDC12* regulated the growth and invasion of colon cancer through Snail. Through our series of experiments, we can infer that *CCDC12* promotes colon carcinogenesis and the EMT process by promoting the expression of Snail.

## Materials and methods

### Association and eQTL analysis based on colorectal cancer-associated SNPs

Colorectal cancer-associated SNPs were extracted from the NHGRI GWAS database (Table S5). The datasets for germline genotypes, ancestry verification, expression, methylation, somatic copy number aberrations, germline copy number aberrations were downloaded from the TCGA portal. SNP loci with minor allele frequency (MAF) > 0.05 from TCGA (subjects) and HapMap cell lines (controls) were downloaded on EIGENSTRAT and the top two principal components were retrieved (Fig. S9). The average segmented copy-number scores of genetic intervals between the transcription start and end sites set as gene-based somatic copy-number, and CpG methylation value identified with cut-off values of 0.2, 0.4, 0.6, 0.8, and 1.0 (Fig. S10). EQTL analysis was performed according to the flowchart from Li et al. [31] and detailed steps showed in Supplementary methods 1.

### Ancestry verification samples, tissue samples, and tissue microarrays

130 cases of COAD patients for ancestry verification and 12 fresh paired tissues from Shandong Provincial Hospital. Germline genotypes were measured with patients’ peripheral blood and matched tumor samples. All patients hadn’t received preoperative radiotherapy or chemotherapy. Tissue microarrays purchased from Molbase Co. Ltd. (Shanghai, China), consisted of 75 paired human COAD and adjacent colon tissue. This study was approved by the Ethics Committee of Shandong Provincial Hospital. All procedures were performed following the International Ethical Guidelines for Biomedical Research Involving Human Subjects and Declaration of Helsinki. Written informed consent from all patients was obtained. The sample size was calculated to fit the experimental requirements.

### Cell lines and RNA interference or overexpression

HCT116, T84, LOVO, SW480, RKO, and CCD-18Co cell lines were obtained from the Chinese Tissue Culture Collections (CTCC, China) with STR confirmed. Cell culture environment was described in Supplementary methods 2. Three *CCDC12* short hairpin RNA (shRNA) were transfected into SW480 and LOVO cell lines for 24 hs at 37 °C (KD group). Lentivirus with puromycin resistance was used to overexpress *CCDC12* in HCT116 cells (OE group) and interfered with SW480 (SW480-KD, transfected CCDC12-shRNA). Lentivirus expressing *SNAI1* infected SW480 cell line (OE-Snail group) and expressed red fluorescent protein. 72 h after transfection, exposed to puromycin for 48 h or observed with a fluorescent microscope (Olympus, IX71, Japan) to select. All shRNA and lentivirus were designed and synthesized by Genechem (Shanghai, China)

### Immunohistochemistry staining, western blotting, and real-time quantitative polymerase chain reaction

IHC staining was performed using a Power-Vision two-step tissue staining kit (ZSGB-BIO, Cat. PV-6001, Beijing, China) referring to the manual and the results were evaluated using H-scores by 3 researchers independently. Extracted proteins from cells and tissues were electrophoresed on a 10% SDS-PAGE and then transferred onto a 0.45 μm Immobilon-P Transfer Membrane (Millipore, Cat.IPVH00010, USA) using the wet transfer method. Incubated with primary antibody overnight at 4 °C and sequentially incubated with corresponding secondary antibody for 1 h at room temperature. Bands were visualized through the ECL kit (Millipore, Cat.WBKLS0500, USA) and Amersham Imager 680 system. Total RNA was extracted and reverse transcribed to cDNA through Reverse Transcription System (Promega, USA). Quantitative RT-qPCR was performed with SYBR Green qPCR SuperMix (Invitrogen, USA) and ABI PRISM® 7500 Sequence Detection System based on the manufacturer’s instructions. 18srRNA was used as the internal reference control with the ΔΔCT method. Antibodies and primer sequences are listed in Supplementary Methods 3–4.

### Tumor-related phenotypic experiments

In the colony-forming assay, 100 cells in the logarithmic growth phase were seeded into a six-well plate. When colony formation, stained with 1% crystal violet solution for 20 min and counted under a microscope. For the MTS assay, 1 × 10^4^ cells were seeded into a 96-well plate. CellTiter 96^®^ A Q_ueous_ One Solution Cell Proliferation Assay (Promega, Cat.G3582, USA) was used to measure cell proliferation and was performed based on the manufacturer’s instructions. OD was measured using a Multiscan MK3 microplate reader at 490 nm. Monolayer cell was scraped off via a pipette tip in the middle of the plate when 95% confluent. Cell migration was measured every 6 h using the Image Pro-Plus 6.0. Transwell chambers (BD, Cat.353097) with Matrigel (BD, Cat.356234, USA) were used for invasion assays. 1 × 10^5^ cells with the serum-free medium were placed in the upper chamber. The bottom chamber contained a medium with 20% serum. 4% paraformaldehyde was used to fix the cells and stained with crystal violet solution.

The Annexin V-FITC apoptosis detection kit (Keygen, Cat.KGA106, Jiangsu, China) was used per the manufacturer’s instructions. 1.25 μl Annexin V-FITC reagent was added to 500 μl of cell suspension (1 × 10^6^/mL) and then incubated for 15 min at room temperature in the dark. After centrifuging at 1000 × *g* for 5 min, the supernatant was removed and resuspended in 0.5 mL pre-cooled binding buffer. Then, 10 μl Propidium Iodide was added and incubated in the dark before being read on the BD FACSCalibur CellSorting System. Based on Cell Cycle Detection Kit (Keygen, Cat.KGA511, Jiangsu, China), 5 μl (10 mg/mL) of RNase A was added to cells and incubated at 37 °C for 1 h. Afterward, 50 μg/mL PI and 0.2% Triton X-100 were added and incubated at 4 °C in the dark for 30 min. BD FACSCalibur CellSorting System was used to measure cell cycle phases. 2–3 × 10^4^ cells were counted and analyzed using ModFit software.

### Xenograft mouse models

4-week-old BALB/c nude mice (*n* = 5 each group) were purchased from Charles River Laboratories (Beijing, China) fed on an ordinary diet. Xenograft tumors were established by subcutaneous injection of 200 μl cell suspension (5 × 10^5^ cells in KD group and 2 × 10^5^ cells in OE group with their respective control groups) into the underarms or backs of nude mice. Mice were euthanized 30 days after inoculation, and tumors were removed for subsequent analysis. The animal experiments were approved by the Animal Care and Use Committee of Shandong Provincial Hospital.

### 4 plex isobaric tags for relative and absolute quantitation (iTRAQ) assays

Proteins were extracted from *CCDC12* over-expressing HCT116 cells and control HCT116 cells. The Bradford quantitative method was used to determine the total protein content. Proteins were reductively alkylated using DTT and TEAB. After trypsin (full) digestion for 16 h at 37 °C, peptides were acidified with 0.1% FA. The components were grouped based on high pH C18 Column (5 μm, 100 Å, 4.6 × 250 mm, Durashell C18) with Thermo DINOEX Ultimate 3000 BioRS LC system, and the isolated components were analyzed by LC-MS/MS (Thermo Fisher Q-exactive HF-X) with ChromXP C18 Column (3 μm, 120 Å, 7.5 × 100 mm, AB SCIEX), and then captured via eksigent Chromxp Trap Column (3 μm C18-CL, 120 Å, 350 μm × 0.5 mm). Proteome Discoverer 2.2 was employed to analyze raw data and the protein database is UniProt-human-9606-20181130.fasta. Specifically, we set precursor ion mass tolerance as ± 10 ppm, fragment ion mass tolerance as ± 0.02 Da, and max missed cleavages is 2. To improve the quality of the analysis results and reduce the false positive rate, the confidence level of peptides and proteins meets FDR ≤ 1% while containing at least one unique peptide considered as a plausible protein. The results were analyzed through Cluster of COG analysis, GO analysis, and (KEGG pathway enrichment. Information on the chromatography columns is in Supplementary Methods 5. The threshold of fold change (FC) was set at ≥ 1.3 or ≤ 0.77 with a *q* value < 0.05.

### Statistical analysis

Statistical analysis was performed using R 3.5.1. Data were expressed as mean ± SD. One-way analysis of variance and Student’s T-test was used to analyze differences among groups. χ^2^ test or linear correlation was used to determine the correlation between *CCDC12* expression and clinicopathological features. Kaplan–Meier method was used to generate survival curves with a log-rank test. MAF > 0.05, FDR < 0.1, α = 0.05 and *P* < 0.05 with two sides were considered statistically significant.

## Supplementary information

Supplementary figures, tables, and methods for online publication.

Reproducibility checklist

## Data Availability

The datasets generated and/or analyzed during the current study are available in the PRIDE repository with ProteomeXchange accession as PXD029620.
