# Correction to: *CCDC12* promotes tumor development and invasion through the Snail pathway in colon adenocarcinoma

**DOI:** 10.1038/s41419-022-04742-8

**Published:** 2022-03-28

**Authors:** Fengying Du, Lipan Peng, Qiang Wang, Kangdi Dong, Wenting Pei, Hongqing Zhuo, Tao Xu, Changqing Jing, Leping Li, Jizhun Zhang

**Affiliations:** 1 Department of Gastrointestinal Surgery, Shandong Provincial Hospital Affiliated to Shandong First Medical University, Jinan, Shandong China; 2 Cheeloo College of Medicine, Shandong University, Jinan, Shandong China; 3 Personnel Office, Shandong Provincial Hospital Affiliated to Shandong First Medical University, Jinan, Shandong China; 4 Hematology and Oncology, Qilu Children’s Hospital of Shandong University, Jinan, Shandong China; 5 Center for Post-doctoral Studies, Shandong University of Traditional Chinese Medicine, Jinan, Shandong China

**Keywords:** Diagnostic markers, Colon cancer

Correction to: *Cell Death & Disease* 10.1038/s41419-022-04617-y, published online 25 February 2022

The original version of this article unfortunately contained errors in the affiliations. The correct affiliations should be as follows: Fengying Du1,2,6, Lipan Peng1,6, Qiang Wang3, Kangdi Dong1, Wenting Pei4, Hongqing Zhuo1, Tao Xu1, Changqing Jing1, Leping Li1 and Jizhun Zhang1,5

1Department of Gastrointestinal Surgery, Shandong Provincial Hospital Affiliated to Shandong First Medical University, Jinan, Shandong, China.

2Cheeloo College of Medicine, Shandong University, Jinan, Shandong, China.

3Personnel Office, Shandong Provincial Hospital Affiliated to Shandong First Medical University, Jinan, Shandong, China.

4Hematology and Oncology, Qilu Children’s Hospital of Shandong University, Jinan, Shandong, China.

5Center for Post-doctoral Studies, Shandong University of Traditional Chinese Medicine, Jinan, Shandong, China.

6These authors contributed equally: Fengying Du and Lipan Peng. The original article has been corrected.

