# Supplementary material for: CCDC12 promotes tumor development and invasion through the Snail pathway in colon adenocarcinoma
Source: Cell Death Dis. 2022 Feb 25;13(2):187. doi: 10.1038/s41419-022-04617-y (PMC8881494; doi:10.1038/s41419-022-04617-y)
Supplement: Supplementary file 1 — Supplementary figures, tables, and methods for online publication. [file 41419_2022_4617_MOESM1_ESM.docx]

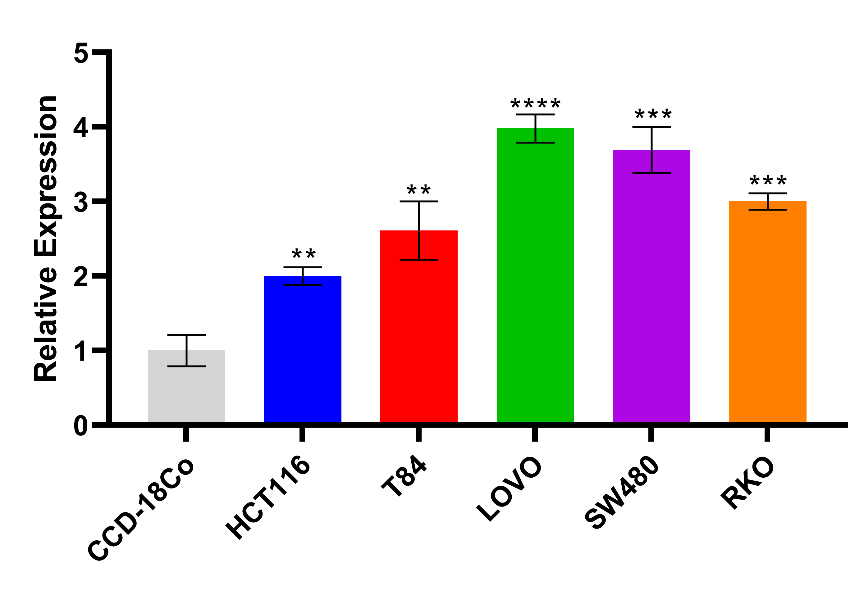


**Figure S1. Relative expression levels of CCDC12 in five colon cancer cell lines and colon fibroblast cells.**


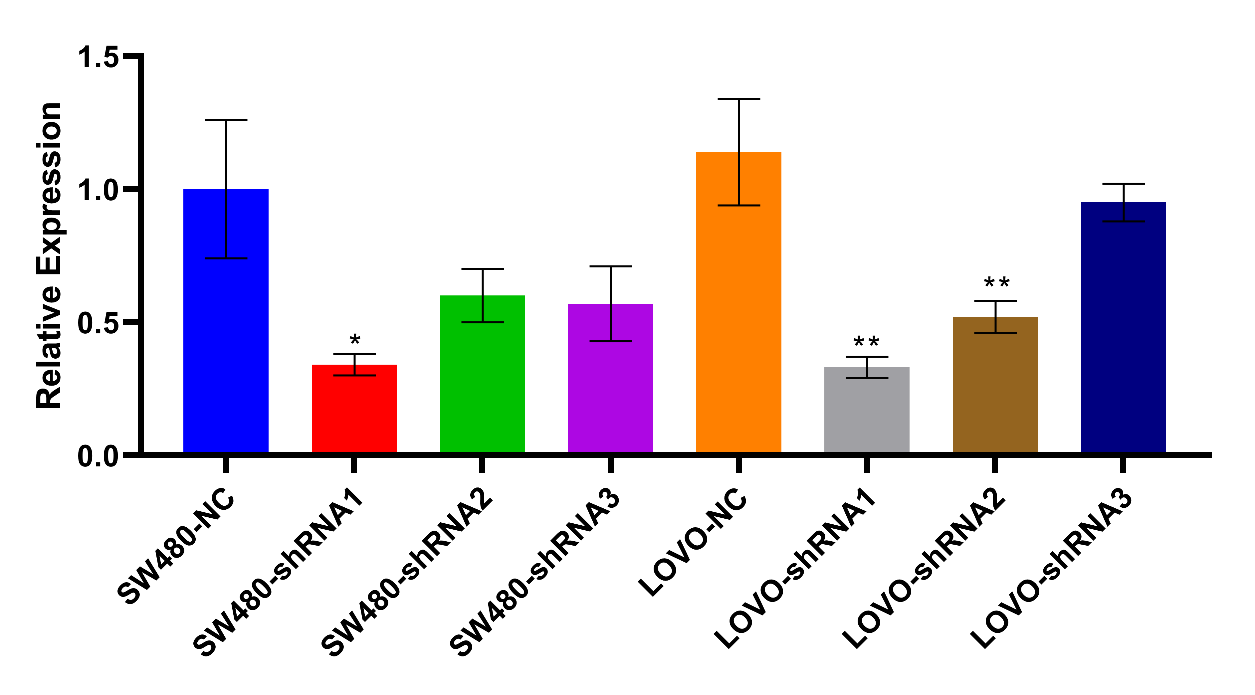


**Figure S2. The potency of the three shRNAs to knockdown CCDC12 expression levels.**


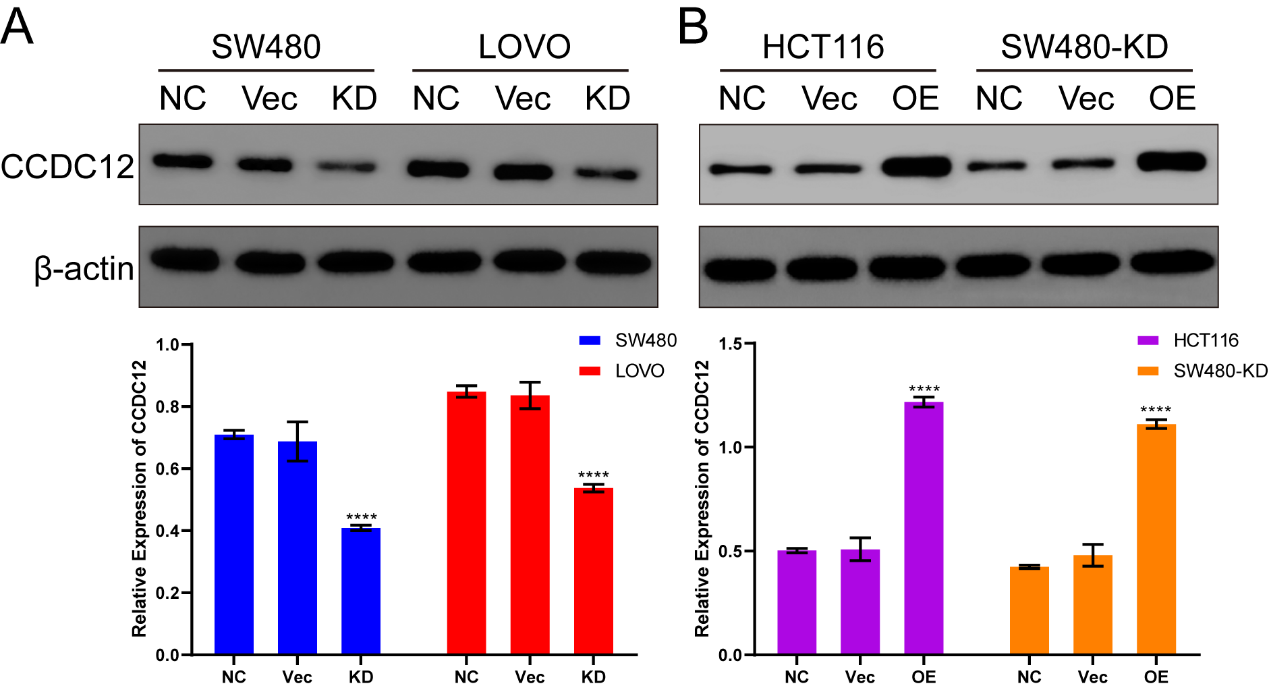


**Figure S3.** **Western blot to verify the efficiency of knockdown and overexpression of CCDC12.** (A) Knockdown of CCDC12. (B) Overexpression of CCDC12.


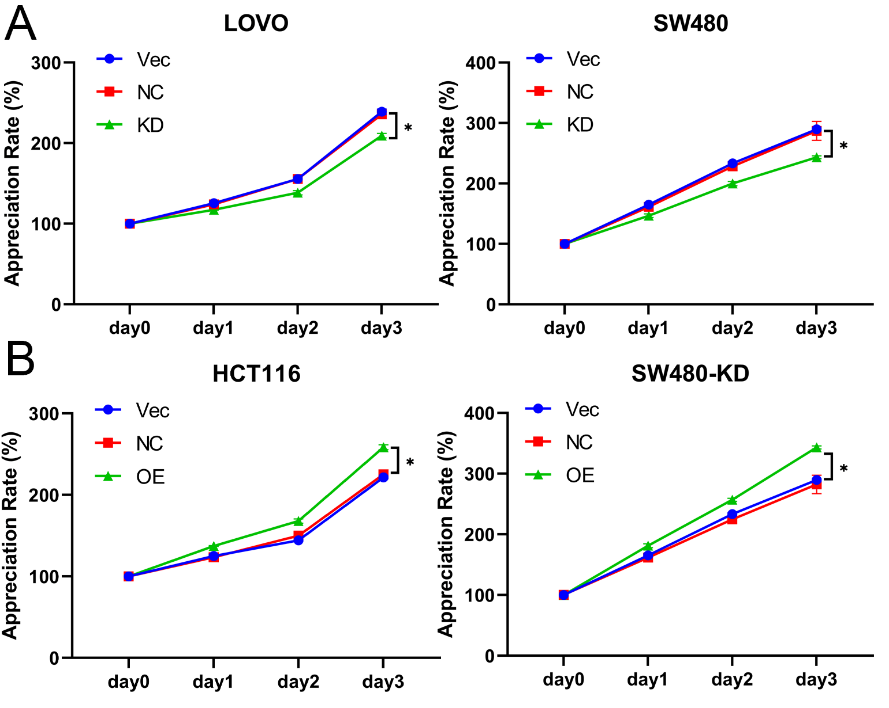


**Figure S4.** **Cell proliferation was determined using MTS assays.** (A) Knockdown of CCDC12. (B) Overexpression of CCDC12.


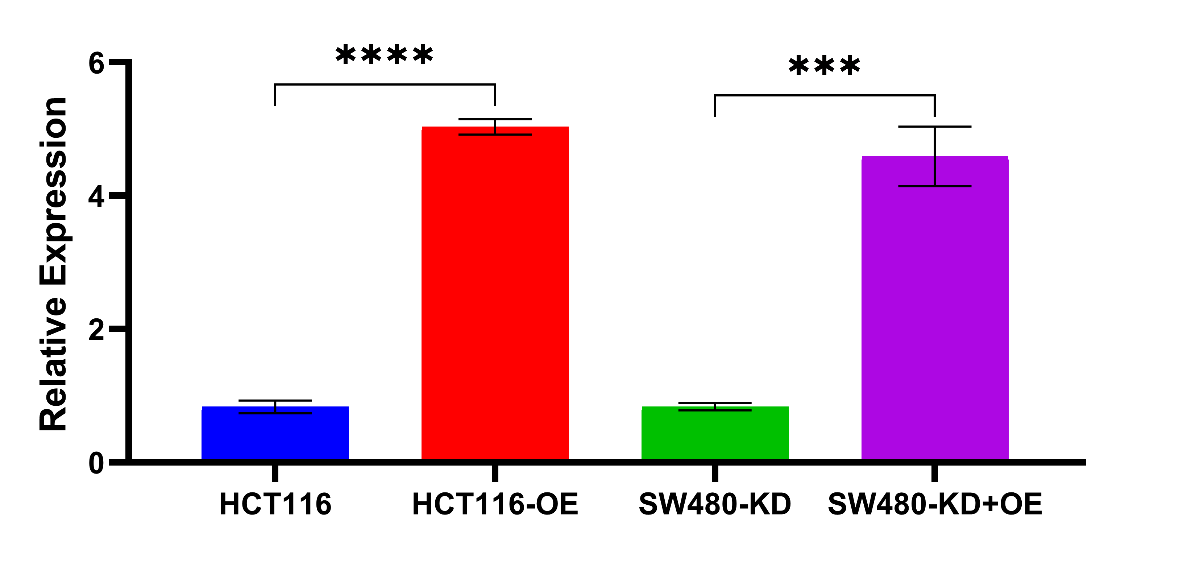


**Figure S5.** **The effect of lentivirus over-expressing CCDC12.**


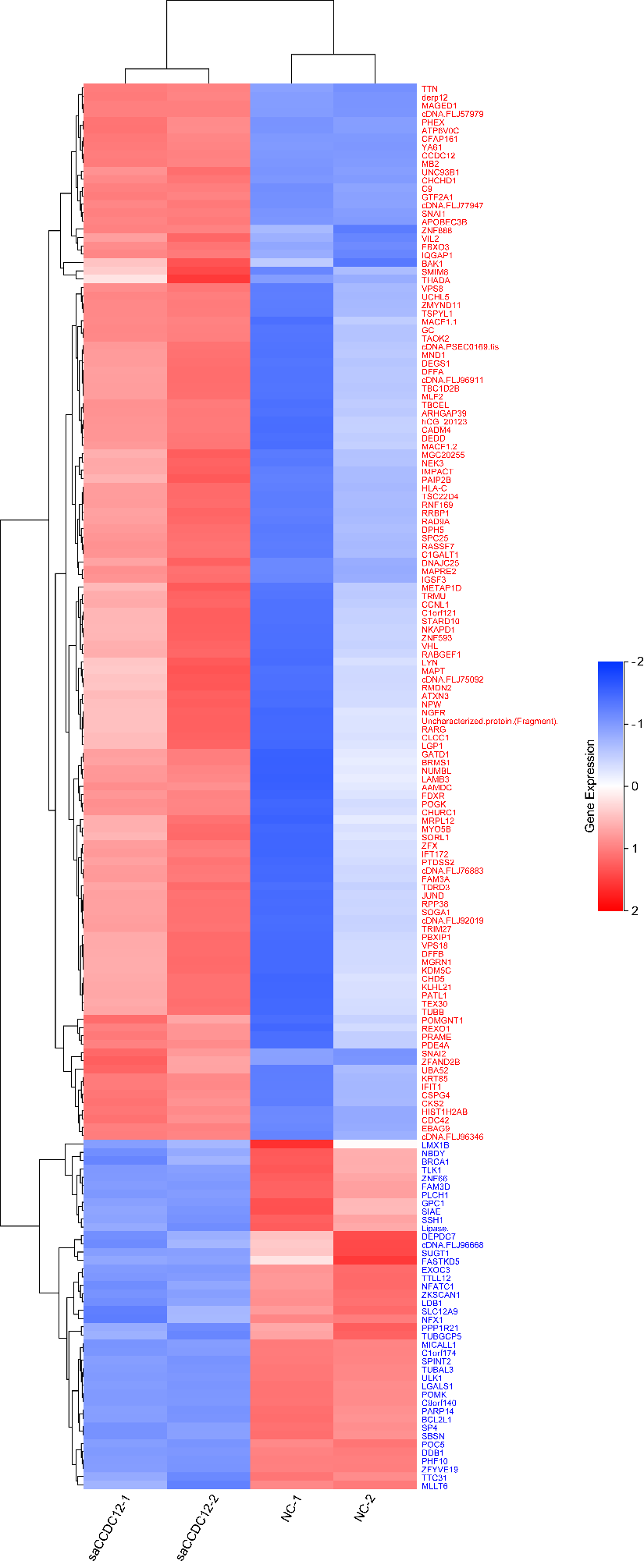


**Figure S6. Cluster heatmap of the full differentially expressed proteins.**


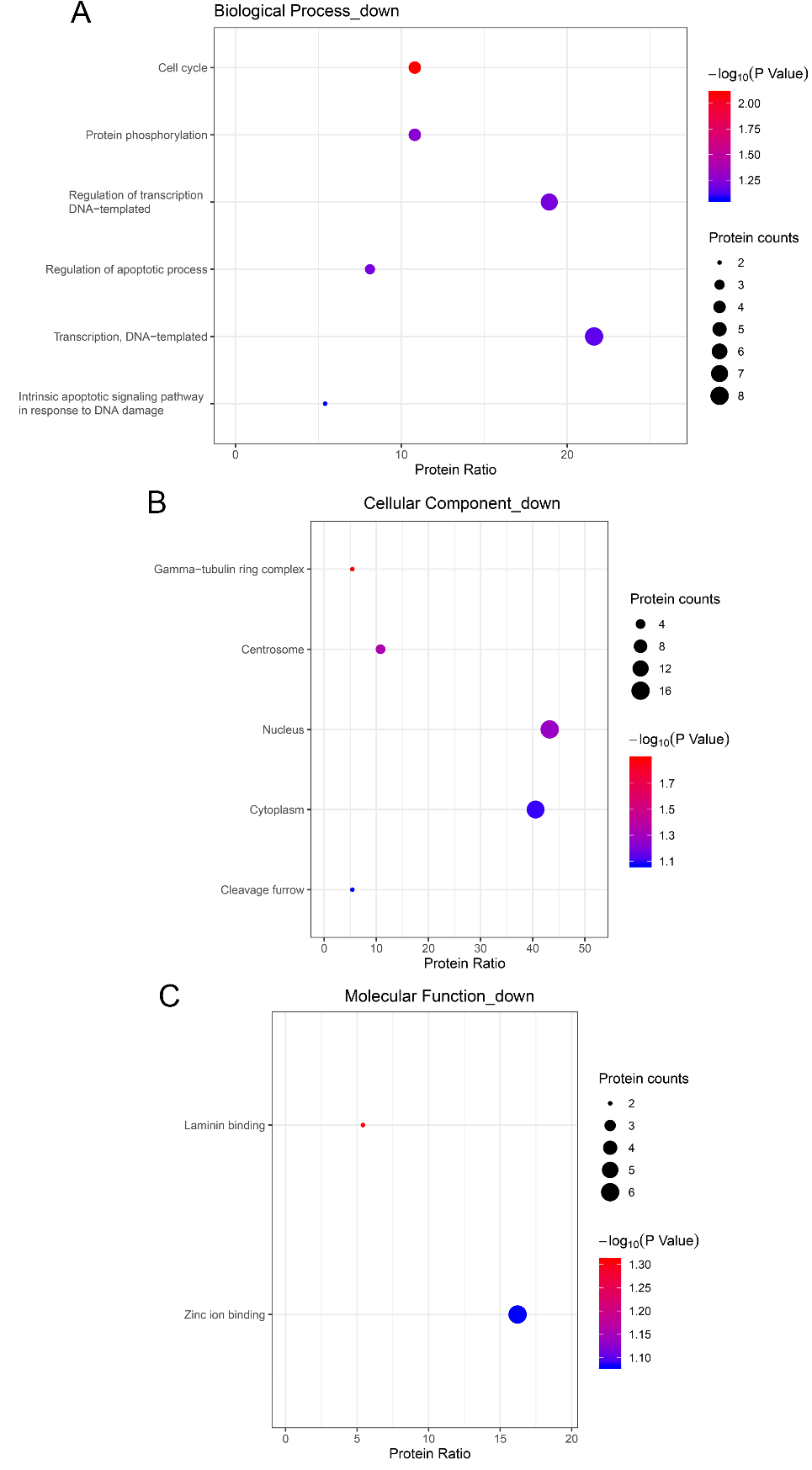


**Figure S7.** **Bioinformatic analysis and validation of differentially expressed proteins.** (A) GO annotation of the increased expressed proteins (BP). (B) GO annotation of the increased expressed proteins (MF). (C) GO annotation of the increased expressed proteins (CC).


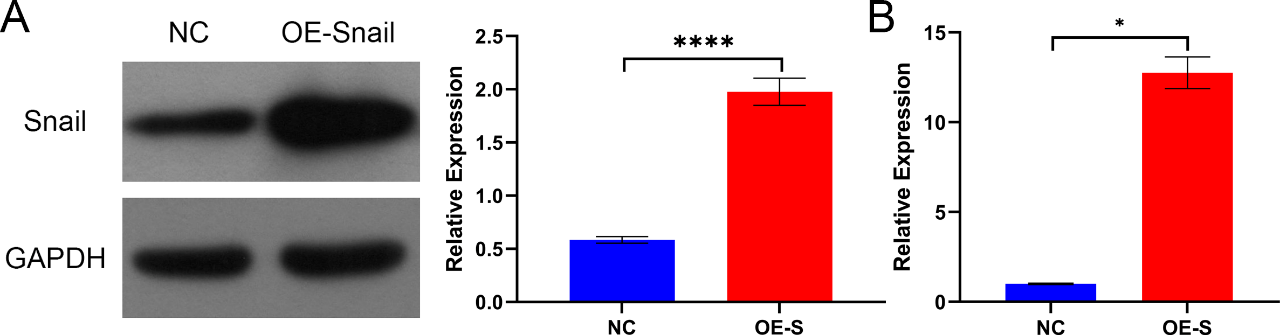


**Figure S8. Confirmation of Snail overexpression.** (A) A western blotting analysis demonstrating Snail overexpression. (B) RT-qPCR demonstrating Snail overexpression.


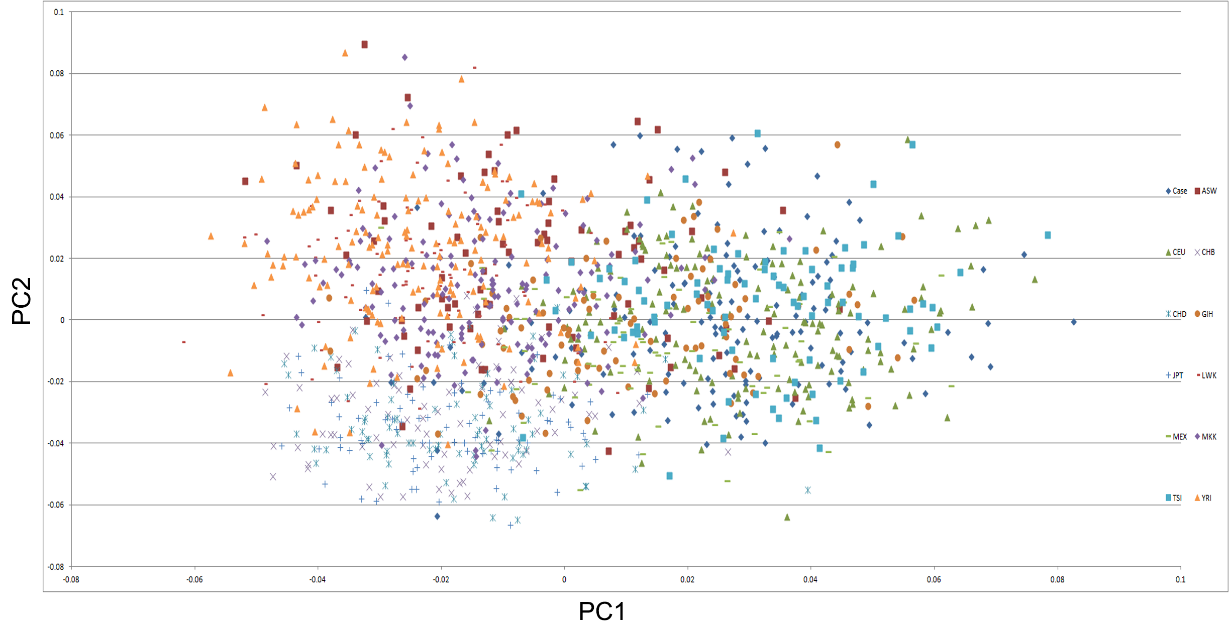


**Figure S9.** **Ancestry verification confirmed 130 samples as HapMap CHB derived from the 147 CRC samples.**


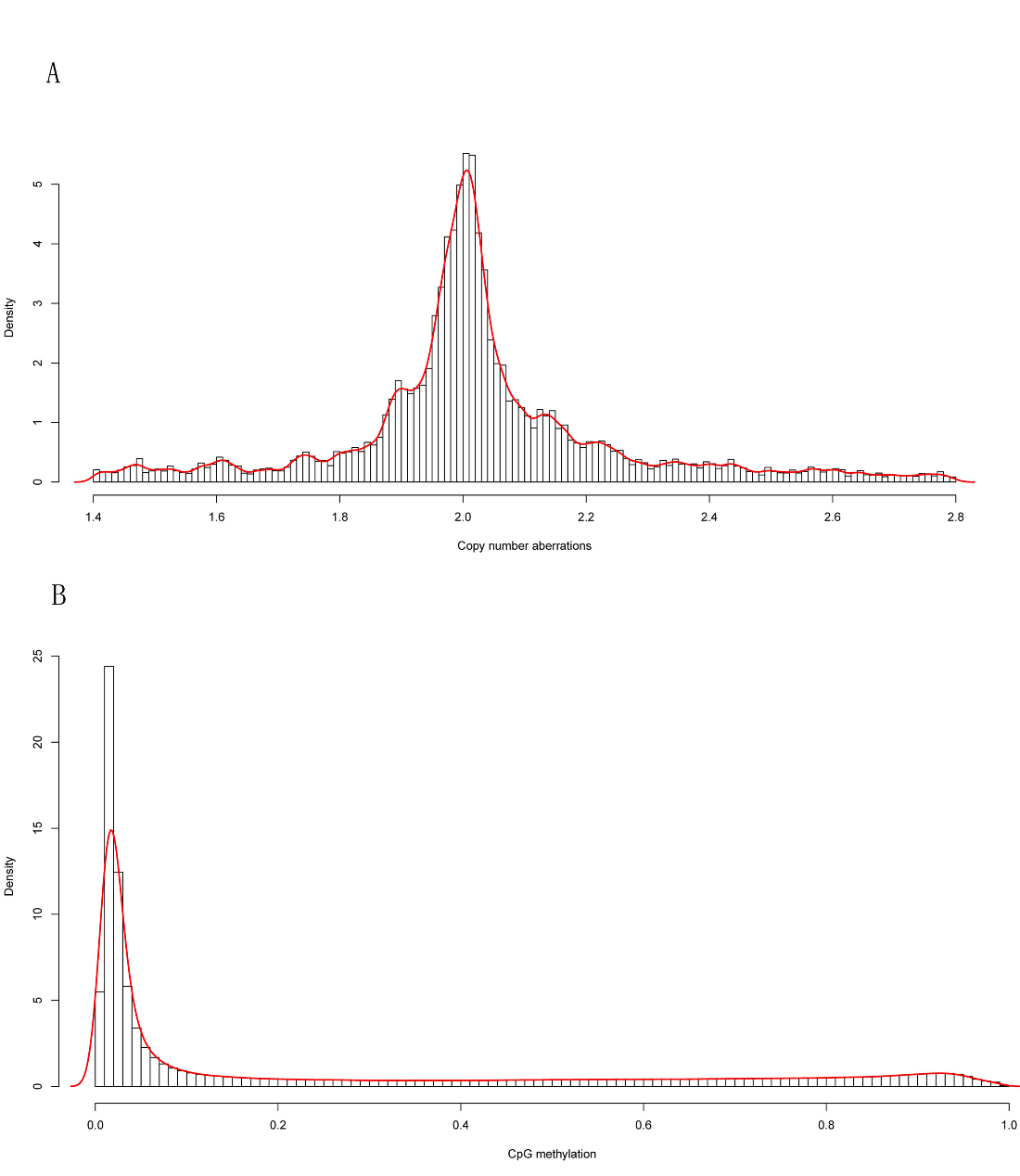


**Figure S10. SNP loci copy number variation and methylation status.** (A) Copy-number variation distribution; (B) CpG methylation distribution in the promoter region.

**Table S1. Clinical and pathologic data of the 130 patients.**

| **Characteristic** | **Sample size** |
| --- | --- |
| **Age at surgery**  ≤60  >60 | 23(17.7%)  107(82.3%) |
| **Sex**  Male  Female | 63(48.5%)  67(51.5%) |
| **T category**  T_1_  T_2_  T_3_  T_4_ | 4(3.1%)  26(20.0%)  88(67.7%)  12(9.2%) |
| **N category**  N_0_  N_1_  N_2_  **TNM category**  Ⅰ  Ⅱ  Ⅲ  Ⅳ | 79(60.8%)  25(19.2%)  26(20.0%)  25(19.2%)  52(40.0%)  34(26.2%)  19(14.6%) |

**Table S2. Significant associations of SNP gene pairs after applying a false discovery rate threshold of less than 0.1.**

| **SNP** | **Chromosome** | **Position** | **Gene** | **t-stat** | ***P-value*** | **R^2^** | **FDR** |
| --- | --- | --- | --- | --- | --- | --- | --- |
| rs2090916 | chr3 | 150307263 | EIF2A | 16.4354 | 2.46E-33 | 0.6760 | 1.24E-26 |
| rs10493821 | chr1 | 89475135 | GBP3 | -14.2253 | 3.96E-28 | 0.6121 | 9.97E-22 |
| rs17433780 | chr1 | 89474818 | GBP3 | -13.8557 | 3.09E-27 | 0.6086 | 5.18E-21 |
| rs6440685 | chr3 | 150327996 | EIF2A | 13.3990 | 3.97E-26 | 0.6010 | 4.99E-20 |
| rs104664 | chr22 | 45711854 | FAM118A | 13.0135 | 3.46E-25 | 0.6146 | 3.48E-19 |
| rs1569413 | chr22 | 45756081 | FAM118A | 12.4769 | 7.18E-24 | 0.5704 | 6.02E-18 |
| rs17434342 | chr1 | 89505742 | GBP3 | -12.2195 | 3.09E-23 | 0.5500 | 2.22E-17 |
| rs6006992 | chr22 | 45742760 | FAM118A | 12.0737 | 7.08E-23 | 0.6376 | 4.45E-17 |
| rs136611 | chr22 | 45794283 | FAM118A | 12.0507 | 8.06E-23 | 0.6107 | 4.51E-17 |
| rs2624 | chr1 | 89518623 | GBP3 | -11.4602 | 2.32E-21 | 0.5047 | 1.17E-15 |
| rs5765272 | chr22 | 45747901 | FAM118A | 10.9929 | 3.33E-20 | 0.5225 | 1.52E-14 |
| rs2056443 | chr13 | 31905942 | B3GALTL | 10.9420 | 4.46E-20 | 0.4962 | 1.87E-14 |
| rs11699044 | chr20 | 34312713 | CPNE1 | 10.9118 | 5.29E-20 | 0.4904 | 2.05E-14 |
| rs8052 | chr3 | 150259942 | EIF2A | 10.8431 | 7.83E-20 | 0.4747 | 2.54E-14 |
| rs4623517 | chr9 | 85602108 | RASEF | 10.8381 | 8.05E-20 | 0.4745 | 2.54E-14 |
| rs1393565 | chr9 | 85605040 | RASEF | 10.8373 | 8.09E-20 | 0.4792 | 2.54E-14 |
| rs939015 | chr11 | 43868651 | HSD17B12 | 10.8022 | 9.88E-20 | 0.4728 | 2.92E-14 |
| rs13080 | chr13 | 31906141 | B3GALTL | 10.7497 | 1.33E-19 | 0.4884 | 3.72E-14 |
| rs4385545 | chr9 | 85601796 | RASEF | 10.6540 | 2.30E-19 | 0.4662 | 5.80E-14 |
| rs1546315 | chr9 | 85641181 | RASEF | 10.6536 | 2.30E-19 | 0.4678 | 5.80E-14 |
| rs6559694 | chr9 | 85613043 | RASEF | 10.6137 | 2.89E-19 | 0.4683 | 6.93E-14 |
| rs4355855 | chr9 | 85601818 | RASEF | 10.5551 | 4.04E-19 | 0.4725 | 9.16E-14 |
| rs4355658 | chr7 | 73225692 | WBSCR27 | -10.5462 | 4.25E-19 | 0.4608 | 9.16E-14 |
| rs11316 | chr7 | 73246555 | WBSCR27 | 10.5355 | 4.52E-19 | 0.4982 | 9.16E-14 |
| rs6060578 | chr20 | 34305445 | CPNE1 | 10.5290 | 4.68E-19 | 0.4885 | 9.16E-14 |
| rs1970357 | chr20 | 34339200 | CPNE1 | 10.5272 | 4.73E-19 | 0.4915 | 9.16E-14 |
| rs10735559 | chr9 | 85596321 | RASEF | 10.4817 | 6.13E-19 | 0.4790 | 1.14E-13 |
| rs11696912 | chr20 | 34497660 | CPNE1 | 10.4752 | 6.36E-19 | 0.4654 | 1.14E-13 |
| rs6060669 | chr20 | 34480557 | CPNE1 | -10.4697 | 6.56E-19 | 0.5000 | 1.14E-13 |
| rs10742690 | chr11 | 43867652 | HSD17B12 | 10.4475 | 7.45E-19 | 0.4560 | 1.25E-13 |
| rs10780563 | chr9 | 85614295 | RASEF | 10.4375 | 7.88E-19 | 0.4650 | 1.28E-13 |
| rs6559695 | chr9 | 85639623 | RASEF | 10.4301 | 8.22E-19 | 0.4596 | 1.29E-13 |
| rs6485455 | chr11 | 43753725 | HSD17B12 | 10.4005 | 9.73E-19 | 0.4547 | 1.48E-13 |
| rs2425071 | chr20 | 34292961 | CPNE1 | -10.3516 | 1.29E-18 | 0.4748 | 1.90E-13 |
| rs1502683 | chr9 | 85599580 | RASEF | 10.3377 | 1.39E-18 | 0.4534 | 2.00E-13 |
| rs1127155 | chr7 | 73246461 | WBSCR27 | 10.3138 | 1.59E-18 | 0.4801 | 2.23E-13 |
| rs10838186 | chr11 | 43869980 | HSD17B12 | 10.2330 | 2.52E-18 | 0.4466 | 3.43E-13 |
| rs6485456 | chr11 | 43766902 | HSD17B12 | 10.1585 | 3.85E-18 | 0.4577 | 5.10E-13 |
| rs136610 | chr22 | 45794212 | FAM118A | 10.1273 | 4.60E-18 | 0.5382 | 5.93E-13 |
| rs871512 | chr9 | 85689581 | RASEF | 10.0655 | 6.52E-18 | 0.4394 | 8.20E-13 |
| rs7019227 | chr9 | 85639259 | RASEF | 10.0604 | 6.72E-18 | 0.4427 | 8.24E-13 |
| rs6058356 | chr20 | 34491530 | CPNE1 | 10.0363 | 7.70E-18 | 0.4583 | 9.22E-13 |
| rs1976455 | chr9 | 85630621 | RASEF | 10.0212 | 8.39E-18 | 0.4490 | 9.81E-13 |
| rs1502682 | chr9 | 85617668 | RASEF | 10.0053 | 9.18E-18 | 0.4466 | 1.05E-12 |
| rs10768983 | chr11 | 43839935 | HSD17B12 | 9.9677 | 1.14E-17 | 0.4566 | 1.27E-12 |
| rs7110437 | chr11 | 43780553 | HSD17B12 | 9.9287 | 1.42E-17 | 0.4932 | 1.55E-12 |
| rs6764672 | chr3 | 150255152 | EIF2A | 9.9186 | 1.50E-17 | 0.4450 | 1.61E-12 |
| rs11907811 | chr20 | 34428078 | CPNE1 | 9.8758 | 1.91E-17 | 0.4690 | 2.00E-12 |
| rs7271036 | chr20 | 34467215 | CPNE1 | 9.8512 | 2.20E-17 | 0.4486 | 2.26E-12 |
| rs3827029 | chr20 | 34332784 | CPNE1 | 9.7810 | 3.27E-17 | 0.4424 | 3.29E-12 |
| rs3740488 | chr10 | 102754033 | MRPL43 | 9.7636 | 3.61E-17 | 0.4271 | 3.56E-12 |
| rs5765327 | chr22 | 45800926 | FAM118A | 9.7540 | 3.81E-17 | 0.4917 | 3.68E-12 |
| rs10780567 | chr9 | 85635135 | RASEF | 9.7403 | 4.11E-17 | 0.4452 | 3.86E-12 |
| rs4717112 | chr7 | 73265417 | WBSCR27 | -9.7388 | 4.15E-17 | 0.4422 | 3.86E-12 |
| rs3824783 | chr10 | 102750851 | MRPL43 | 9.5738 | 1.05E-16 | 0.4236 | 9.58E-12 |
| rs4460464 | chr9 | 85661899 | RASEF | 9.5714 | 1.07E-16 | 0.4127 | 9.58E-12 |
| rs11979680 | chr7 | 73188574 | WBSCR27 | -9.5508 | 1.20E-16 | 0.4398 | 1.06E-11 |
| rs9986447 | chr6 | 42942779 | PEX6 | 9.5237 | 1.40E-16 | 0.4453 | 1.21E-11 |
| rs3827393 | chr22 | 45736153 | FAM118A | 9.5076 | 1.53E-16 | 0.4948 | 1.30E-11 |
| rs6941212 | chr6 | 42915920 | PEX6 | 9.2956 | 5.04E-16 | 0.4364 | 4.22E-11 |
| rs10116216 | chr9 | 85663674 | RASEF | 9.2824 | 5.42E-16 | 0.4116 | 4.47E-11 |
| rs701834 | chr10 | 102761801 | MRPL43 | 9.2075 | 8.25E-16 | 0.3963 | 6.69E-11 |
| rs1721498 | chr7 | 105178819 | RINT1 | -9.1943 | 8.89E-16 | 0.4110 | 7.09E-11 |
| rs1326308 | chr13 | 31935616 | B3GALTL | 9.1512 | 1.13E-15 | 0.3978 | 8.89E-11 |
| rs4919510 | chr10 | 102734778 | MRPL43 | 9.1371 | 1.22E-15 | 0.3960 | 9.47E-11 |
| rs3818554 | chr6 | 42934620 | PEX6 | -9.1145 | 1.39E-15 | 0.3937 | 1.06E-10 |
| rs912603 | chr13 | 31905641 | B3GALTL | 9.0856 | 1.63E-15 | 0.4014 | 1.22E-10 |
| rs1518814 | chr11 | 43824633 | HSD17B12 | 9.0834 | 1.65E-15 | 0.4305 | 1.22E-10 |
| rs6060677 | chr20 | 34499368 | CPNE1 | 9.0516 | 1.97E-15 | 0.4253 | 1.44E-10 |
| rs4717821 | chr7 | 73240993 | WBSCR27 | 9.0436 | 2.06E-15 | 0.4571 | 1.48E-10 |
| rs7854101 | chr9 | 85612533 | RASEF | 9.0207 | 2.35E-15 | 0.4340 | 1.66E-10 |
| rs1582192 | chr1 | 233525193 | KIAA1804 | 8.9940 | 2.72E-15 | 0.3911 | 1.90E-10 |
| rs2425132 | chr20 | 34338390 | CPNE1 | -8.9855 | 2.85E-15 | 0.4510 | 1.97E-10 |
| rs8629 | chr7 | 73245519 | WBSCR27 | -8.9746 | 3.03E-15 | 0.4167 | 2.06E-10 |
| rs6949053 | chr7 | 73269937 | WBSCR27 | 8.9499 | 3.48E-15 | 0.4331 | 2.33E-10 |
| rs3763236 | chr6 | 42902508 | PEX6 | 8.8891 | 4.88E-15 | 0.4079 | 3.23E-10 |
| rs2274517 | chr6 | 42932715 | PEX6 | -8.8851 | 4.99E-15 | 0.3944 | 3.26E-10 |
| rs1121122 | chr13 | 31941210 | B3GALTL | 8.8424 | 6.33E-15 | 0.3833 | 4.08E-10 |
| rs4917916 | chr10 | 102673292 | MRPL43 | 8.7931 | 8.32E-15 | 0.3809 | 5.30E-10 |
| rs324019 | chr12 | 57486647 | STAT6 | -8.7606 | 9.96E-15 | 0.4152 | 6.26E-10 |
| rs9545125 | chr13 | 31911822 | B3GALTL | 8.7109 | 1.31E-14 | 0.3944 | 8.15E-10 |
| rs701835 | chr10 | 102762127 | MRPL43 | 8.6824 | 1.54E-14 | 0.3834 | 9.43E-10 |
| rs9683679 | chr4 | 57278315 | PPAT | -8.6395 | 1.95E-14 | 0.3634 | 1.18E-09 |
| rs9684298 | chr4 | 57278167 | PPAT | -8.5878 | 2.59E-14 | 0.3647 | 1.55E-09 |
| rs7911957 | chr10 | 102692970 | MRPL43 | 8.5831 | 2.66E-14 | 0.3706 | 1.57E-09 |
| rs6120998 | chr20 | 34187313 | CPNE1 | -8.5597 | 3.03E-14 | 0.3656 | 1.77E-09 |
| rs17093026 | chr20 | 34318911 | CPNE1 | -8.5412 | 3.35E-14 | 0.4627 | 1.94E-09 |
| rs3829829 | chr20 | 34537160 | CPNE1 | 8.4919 | 4.40E-14 | 0.4389 | 2.51E-09 |
| rs7683781 | chr4 | 57186686 | PPAT | -8.4486 | 5.58E-14 | 0.3675 | 3.15E-09 |
| rs1355614 | chr7 | 105179176 | RINT1 | -8.3945 | 7.51E-14 | 0.3824 | 4.20E-09 |
| rs7847666 | chr9 | 85609178 | RASEF | 8.3892 | 7.73E-14 | 0.4909 | 4.27E-09 |
| rs4649307 | chr1 | 233513117 | KIAA1804 | 8.3649 | 8.84E-14 | 0.3892 | 4.83E-09 |
| rs3795375 | chr1 | 233514934 | KIAA1804 | 8.3448 | 9.87E-14 | 0.3708 | 5.34E-09 |
| rs4142706 | chr1 | 89475230 | GBP3 | 8.2548 | 1.61E-13 | 0.3765 | 8.64E-09 |
| rs6957192 | chr7 | 105150255 | RINT1 | -8.2082 | 2.08E-13 | 0.3548 | 1.10E-08 |
| rs10111 | chr11 | 118886117 | TRAPPC4 | 8.1811 | 2.41E-13 | 0.3646 | 1.26E-08 |
| rs162131 | chr5 | 7876751 | MTRR | 8.1750 | 2.49E-13 | 0.3423 | 1.29E-08 |
| rs6510588 | chr19 | 37597395 | ZNF420 | 8.1363 | 3.08E-13 | 0.3454 | 1.58E-08 |
| rs2425137 | chr20 | 34345396 | CPNE1 | 8.1013 | 3.72E-13 | 0.3886 | 1.89E-08 |
| rs9543234 | chr13 | 31848383 | B3GALTL | 8.0979 | 3.79E-13 | 0.3391 | 1.91E-08 |
| rs2741159 | chr12 | 53071382 | KRT1 | 8.0609 | 4.64E-13 | 0.3588 | 2.31E-08 |
| rs1409150 | chr1 | 89473175 | GBP3 | 8.0137 | 5.99E-13 | 0.3303 | 2.96E-08 |
| rs1900463 | chr10 | 71272992 | TSPAN15 | 8.0053 | 6.27E-13 | 0.3348 | 3.06E-08 |
| rs4745990 | chr10 | 71273696 | TSPAN15 | 7.9938 | 6.67E-13 | 0.3332 | 3.23E-08 |
| rs1994516 | chr12 | 53058514 | KRT1 | 7.9919 | 6.74E-13 | 0.3312 | 3.23E-08 |
| rs6943132 | chr7 | 105166776 | RINT1 | -7.9800 | 7.19E-13 | 0.3473 | 3.41E-08 |
| rs4865087 | chr4 | 57194929 | PPAT | -7.9684 | 7.66E-13 | 0.3398 | 3.60E-08 |
| rs3827030 | chr20 | 34474947 | CPNE1 | -7.9432 | 8.77E-13 | 0.3545 | 4.09E-08 |
| rs6060526 | chr20 | 34225302 | CPNE1 | -7.9406 | 8.90E-13 | 0.3553 | 4.11E-08 |
| rs8301 | chr11 | 118895495 | TRAPPC4 | 7.9181 | 1.00E-12 | 0.3441 | 4.59E-08 |
| rs11139921 | chr9 | 85748119 | RASEF | 7.9113 | 1.04E-12 | 0.3291 | 4.72E-08 |
| rs1065483 | chr17 | 5284770 | RABEP1 | -7.8719 | 1.29E-12 | 0.3979 | 5.79E-08 |
| rs10922563 | chr1 | 89546803 | GBP3 | -7.8468 | 1.47E-12 | 0.3291 | 6.57E-08 |
| rs6032544 | chr20 | 44422373 | WFDC3 | -7.7555 | 2.41E-12 | 0.4217 | 1.06E-07 |
| rs10787712 | chr10 | 118414855 | PNLIPRP2 | 7.7351 | 2.69E-12 | 0.3178 | 1.17E-07 |
| rs12062 | chr1 | 46830447 | NSUN4 | -7.7351 | 2.69E-12 | 0.3132 | 1.17E-07 |
| rs6060756 | chr20 | 34608479 | CPNE1 | -7.7306 | 2.75E-12 | 0.3389 | 1.18E-07 |
| rs10786611 | chr10 | 102646342 | MRPL43 | 7.7243 | 2.85E-12 | 0.3638 | 1.21E-07 |
| rs6659228 | chr1 | 46811027 | NSUN4 | -7.6879 | 3.46E-12 | 0.3134 | 1.46E-07 |
| rs17361887 | chr1 | 46834775 | NSUN4 | -7.6735 | 3.74E-12 | 0.3097 | 1.57E-07 |
| rs6060681 | chr20 | 34506147 | CPNE1 | 7.6234 | 4.88E-12 | 0.3262 | 2.03E-07 |
| rs836823 | chr5 | 79943714 | DHFR | -7.5483 | 7.27E-12 | 0.3033 | 3.00E-07 |
| rs6428494 | chr1 | 89472196 | GBP3 | 7.5438 | 7.45E-12 | 0.3152 | 3.05E-07 |
| rs2301179 | chr10 | 118404620 | PNLIPRP2 | 7.5328 | 7.90E-12 | 0.3113 | 3.21E-07 |
| rs4938621 | chr11 | 118892277 | TRAPPC4 | 7.5022 | 9.29E-12 | 0.3005 | 3.74E-07 |
| rs10838184 | chr11 | 43869860 | HSD17B12 | 7.4816 | 1.04E-11 | 0.2988 | 4.14E-07 |
| rs3912630 | chr12 | 53000269 | KRT74 | -7.4674 | 1.12E-11 | 0.4445 | 4.42E-07 |
| rs6485443 | chr11 | 43696316 | HSD17B12 | 7.4637 | 1.14E-11 | 0.2978 | 4.44E-07 |
| rs7116641 | chr11 | 43696917 | HSD17B12 | 7.4637 | 1.14E-11 | 0.2978 | 4.44E-07 |
| rs17361805 | chr1 | 46813967 | NSUN4 | -7.4450 | 1.26E-11 | 0.3035 | 4.87E-07 |
| rs12571521 | chr10 | 118412310 | PNLIPRP2 | 7.4070 | 1.54E-11 | 0.3016 | 5.90E-07 |
| rs11697672 | chr20 | 34624622 | CPNE1 | 7.3989 | 1.60E-11 | 0.3207 | 6.11E-07 |
| rs6684274 | chr1 | 46810842 | NSUN4 | -7.3889 | 1.69E-11 | 0.2997 | 6.30E-07 |
| rs1867986 | chr10 | 118414291 | PNLIPRP2 | 7.3885 | 1.69E-11 | 0.3096 | 6.30E-07 |
| rs41534051 | chr1 | 46828734 | NSUN4 | -7.3881 | 1.70E-11 | 0.3002 | 6.30E-07 |
| rs6032532 | chr20 | 44413410 | WFDC3 | -7.3876 | 1.70E-11 | 0.3068 | 6.30E-07 |
| rs1567759 | chr12 | 53091566 | KRT1 | 7.3805 | 1.77E-11 | 0.3075 | 6.49E-07 |
| rs1402468 | chr19 | 37620248 | ZNF420 | 7.3775 | 1.80E-11 | 0.2936 | 6.54E-07 |
| rs12984458 | chr19 | 37629091 | ZNF420 | 7.3538 | 2.03E-11 | 0.3369 | 7.36E-07 |
| rs7998271 | chr13 | 31862334 | B3GALTL | 7.3517 | 2.06E-11 | 0.3129 | 7.36E-07 |
| rs862245 | chr5 | 81585737 | RPS23 | -7.3512 | 2.06E-11 | 0.2964 | 7.36E-07 |
| rs3826729 | chr19 | 17358133 | USHBP1 | 7.3109 | 2.55E-11 | 0.2929 | 9.03E-07 |
| rs2049229 | chr3 | 150307873 | EIF2A | 7.3087 | 2.58E-11 | 0.3141 | 9.07E-07 |
| rs2562599 | chr19 | 37516374 | ZNF420 | 7.3001 | 2.70E-11 | 0.2968 | 9.42E-07 |
| rs6894520 | chr5 | 81594748 | RPS23 | -7.2903 | 2.84E-11 | 0.2927 | 9.85E-07 |
| rs17596617 | chr11 | 43690717 | HSD17B12 | 7.2847 | 2.92E-11 | 0.2876 | 1.01E-06 |
| rs1408343 | chr10 | 102648765 | MRPL43 | 7.2772 | 3.04E-11 | 0.2975 | 1.04E-06 |
| rs1382540 | chr5 | 79952165 | DHFR | -7.2515 | 3.48E-11 | 0.3032 | 1.18E-06 |
| rs1910261 | chr9 | 85747562 | RASEF | 7.2329 | 3.84E-11 | 0.2848 | 1.29E-06 |
| rs6695043 | chr1 | 46844530 | NSUN4 | -7.2279 | 3.94E-11 | 0.2961 | 1.32E-06 |
| rs3745167 | chr19 | 17360864 | USHBP1 | 7.2112 | 4.30E-11 | 0.2870 | 1.43E-06 |
| rs6060710 | chr20 | 34536055 | CPNE1 | -7.2094 | 4.34E-11 | 0.3350 | 1.44E-06 |
| rs327581 | chr5 | 7914554 | MTRR | 7.1999 | 4.56E-11 | 0.2987 | 1.50E-06 |
| rs9436748 | chr1 | 65911672 | LEPROT | -7.1940 | 4.70E-11 | 0.2985 | 1.52E-06 |
| rs7544663 | chr1 | 113089006 | ST7L | 7.1939 | 4.70E-11 | 0.2926 | 1.52E-06 |
| rs9315123 | chr13 | 31893030 | B3GALTL | 7.1931 | 4.72E-11 | 0.2973 | 1.52E-06 |
| rs2385374 | chr19 | 37583017 | ZNF420 | 7.1917 | 4.76E-11 | 0.2888 | 1.52E-06 |
| rs351372 | chr1 | 113059220 | ST7L | 7.1853 | 4.92E-11 | 0.2887 | 1.57E-06 |
| rs12385696 | chr1 | 46818836 | NSUN4 | -7.1828 | 4.98E-11 | 0.2870 | 1.58E-06 |
| rs7248948 | chr19 | 37547219 | ZNF420 | 7.1452 | 6.06E-11 | 0.2869 | 1.91E-06 |
| rs4262227 | chr7 | 73258638 | WBSCR27 | 7.1417 | 6.17E-11 | 0.2895 | 1.93E-06 |
| rs7253637 | chr19 | 52018717 | SIGLEC12 | -7.1395 | 6.24E-11 | 0.2995 | 1.94E-06 |
| rs1650665 | chr5 | 79962578 | DHFR | -7.1321 | 6.49E-11 | 0.2882 | 2.00E-06 |
| rs7519323 | chr1 | 89575088 | GBP3 | -7.1284 | 6.61E-11 | 0.2845 | 2.03E-06 |
| rs4702507 | chr5 | 7924130 | MTRR | 7.0780 | 8.59E-11 | 0.2757 | 2.62E-06 |
| rs10923326 | chr1 | 118049465 | MAN1A2 | 7.0658 | 9.15E-11 | 0.2749 | 2.75E-06 |
| rs4703879 | chr5 | 81553815 | RPS23 | -7.0645 | 9.21E-11 | 0.2755 | 2.75E-06 |
| rs10923295 | chr1 | 117981213 | MAN1A2 | 7.0639 | 9.24E-11 | 0.2749 | 2.75E-06 |
| rs10923312 | chr1 | 118017634 | MAN1A2 | 7.0639 | 9.24E-11 | 0.2749 | 2.75E-06 |
| rs6032530 | chr20 | 44412463 | WFDC3 | -7.0448 | 1.02E-10 | 0.2818 | 3.02E-06 |
| rs7591929 | chr2 | 190643257 | ORMDL1 | 7.0329 | 1.09E-10 | 0.2820 | 3.19E-06 |
| rs17357759 | chr1 | 46832534 | NSUN4 | -7.0313 | 1.09E-10 | 0.3262 | 3.20E-06 |
| rs12385693 | chr1 | 46818921 | NSUN4 | -7.0287 | 1.11E-10 | 0.2962 | 3.22E-06 |
| rs4233336 | chr1 | 89619792 | GBP3 | -7.0233 | 1.14E-10 | 0.2737 | 3.29E-06 |
| rs7245882 | chr19 | 37580415 | ZNF420 | 7.0177 | 1.17E-10 | 0.2763 | 3.37E-06 |
| rs3766942 | chr1 | 89586305 | GBP3 | -6.9942 | 1.33E-10 | 0.2731 | 3.79E-06 |
| rs1058398 | chr17 | 5288760 | RABEP1 | -6.9916 | 1.34E-10 | 0.3456 | 3.82E-06 |
| rs9993277 | chr4 | 57174134 | PPAT | -6.9877 | 1.37E-10 | 0.2831 | 3.87E-06 |
| rs2864104 | chr19 | 52030370 | SIGLEC12 | -6.9746 | 1.47E-10 | 0.2761 | 4.12E-06 |
| rs4806407 | chr19 | 37588751 | ZNF420 | 6.9717 | 1.49E-10 | 0.2856 | 4.16E-06 |
| rs619178 | chr1 | 89615702 | GBP3 | -6.9562 | 1.61E-10 | 0.2714 | 4.48E-06 |
| rs1628627 | chr5 | 79967262 | DHFR | -6.9423 | 1.73E-10 | 0.2770 | 4.78E-06 |
| rs1643650 | chr5 | 79940143 | DHFR | -6.9357 | 1.79E-10 | 0.3030 | 4.92E-06 |
| rs3791767 | chr2 | 190639915 | ORMDL1 | 6.9331 | 1.81E-10 | 0.2733 | 4.96E-06 |
| rs6060625 | chr20 | 34414318 | CPNE1 | 6.9300 | 1.84E-10 | 0.3135 | 5.01E-06 |
| rs8056929 | chr16 | 30570011 | ZNF764 | 6.9279 | 1.86E-10 | 0.2670 | 5.04E-06 |
| rs6685187 | chr1 | 151858888 | THEM4 | 6.9126 | 2.02E-10 | 0.2703 | 5.40E-06 |
| rs699113 | chr5 | 10265076 | CCT5 | -6.9123 | 2.02E-10 | 0.2676 | 5.40E-06 |
| rs1359636 | chr13 | 31940061 | B3GALTL | 6.9065 | 2.08E-10 | 0.3069 | 5.54E-06 |
| rs2897262 | chr5 | 79962676 | DHFR | -6.8928 | 2.23E-10 | 0.2757 | 5.91E-06 |
| rs4167 | chr4 | 57186824 | PPAT | -6.8898 | 2.27E-10 | 0.3207 | 5.97E-06 |
| rs893784 | chr2 | 190536283 | ORMDL1 | 6.8878 | 2.29E-10 | 0.2695 | 6.00E-06 |
| rs6485438 | chr11 | 43686618 | HSD17B12 | 6.8819 | 2.36E-10 | 0.2737 | 6.15E-06 |
| rs11133439 | chr4 | 57254335 | PPAT | -6.8778 | 2.41E-10 | 0.2641 | 6.25E-06 |
| rs11238377 | chr7 | 55776532 | PSPH | 6.8578 | 2.67E-10 | 0.2647 | 6.89E-06 |
| rs17172062 | chr7 | 42494790 | HECW1 | -6.8534 | 2.73E-10 | 0.2636 | 7.01E-06 |
| rs2244964 | chr5 | 10263725 | CCT5 | -6.8452 | 2.85E-10 | 0.2625 | 7.22E-06 |
| rs2662532 | chr5 | 10267090 | CCT5 | -6.8452 | 2.85E-10 | 0.2625 | 7.22E-06 |
| rs575250 | chr18 | 13151879 | CIDEA | -6.8445 | 2.86E-10 | 0.2978 | 7.22E-06 |
| rs2578619 | chr5 | 10248797 | CCT5 | -6.8394 | 2.93E-10 | 0.2619 | 7.38E-06 |
| rs1677626 | chr5 | 79949445 | DHFR | -6.8258 | 3.15E-10 | 0.2992 | 7.87E-06 |
| rs1650653 | chr5 | 79965257 | DHFR | -6.8169 | 3.29E-10 | 0.2974 | 8.20E-06 |
| rs2244960 | chr5 | 10263617 | CCT5 | -6.8029 | 3.53E-10 | 0.2607 | 8.72E-06 |
| rs1677703 | chr5 | 79957737 | DHFR | -6.8027 | 3.54E-10 | 0.2658 | 8.72E-06 |
| rs591357 | chr12 | 53050383 | KRT1 | 6.8003 | 3.58E-10 | 0.2707 | 8.79E-06 |
| rs607131 | chr1 | 89656244 | GBP3 | -6.7882 | 3.81E-10 | 0.2601 | 9.30E-06 |
| rs7928523 | chr11 | 43719101 | HSD17B12 | 6.7861 | 3.85E-10 | 0.2775 | 9.35E-06 |
| rs3807306 | chr7 | 128580680 | IRF5 | 6.7847 | 3.88E-10 | 0.2672 | 9.35E-06 |
| rs4755722 | chr11 | 43636578 | HSD17B12 | 6.7843 | 3.89E-10 | 0.2793 | 9.35E-06 |
| rs2292985 | chr4 | 57189498 | PPAT | -6.7816 | 3.94E-10 | 0.2586 | 9.44E-06 |
| rs2071487 | chr1 | 110233081 | GSTM1 | 6.7762 | 4.05E-10 | 0.3962 | 9.65E-06 |
| rs7693137 | chr4 | 57214772 | PPAT | -6.7685 | 4.21E-10 | 0.2685 | 9.99E-06 |
| rs2590965 | chr20 | 34613120 | CPNE1 | 6.7426 | 4.80E-10 | 0.2648 | 1.13E-05 |
| rs11084544 | chr19 | 58940127 | ZNF584 | -6.7404 | 4.86E-10 | 0.2562 | 1.14E-05 |
| rs4755726 | chr11 | 43642130 | HSD17B12 | 6.7385 | 4.90E-10 | 0.2561 | 1.15E-05 |
| rs17245188 | chr5 | 81469082 | RPS23 | -6.7360 | 4.97E-10 | 0.2698 | 1.16E-05 |
| rs1227969 | chr10 | 71263264 | TSPAN15 | 6.7291 | 5.14E-10 | 0.2555 | 1.19E-05 |
| rs6032545 | chr20 | 44422705 | WFDC3 | -6.7222 | 5.33E-10 | 0.2823 | 1.23E-05 |
| rs2862996 | chr11 | 43653833 | HSD17B12 | 6.7074 | 5.74E-10 | 0.2548 | 1.32E-05 |
| rs10481758 | chr9 | 85749941 | RASEF | 6.7039 | 5.85E-10 | 0.2793 | 1.34E-05 |
| rs738808 | chr22 | 24488461 | SUSD2 | 6.6979 | 6.03E-10 | 0.2537 | 1.37E-05 |
| rs1644666 | chr19 | 37475486 | ZNF420 | 6.6969 | 6.06E-10 | 0.2542 | 1.37E-05 |
| rs9543606 | chr13 | 31860769 | B3GALTL | 6.6890 | 6.30E-10 | 0.2709 | 1.42E-05 |
| rs5743061 | chr2 | 190694266 | ORMDL1 | 6.6850 | 6.43E-10 | 0.2962 | 1.44E-05 |
| rs6888977 | chr5 | 81533735 | RPS23 | -6.6846 | 6.45E-10 | 0.2710 | 1.44E-05 |
| rs176159 | chr22 | 24514352 | SUSD2 | 6.6840 | 6.47E-10 | 0.2575 | 1.44E-05 |
| rs3787166 | chr20 | 34229072 | CPNE1 | -6.6759 | 6.74E-10 | 0.2663 | 1.49E-05 |
| rs1667337 | chr19 | 37467512 | ZNF420 | 6.6712 | 6.90E-10 | 0.2570 | 1.52E-05 |
| rs1984490 | chr1 | 46858734 | NSUN4 | -6.6674 | 7.03E-10 | 0.2664 | 1.54E-05 |
| rs2218584 | chr10 | 118408642 | PNLIPRP2 | -6.6610 | 7.26E-10 | 0.2682 | 1.59E-05 |
| rs2548553 | chr5 | 10247965 | CCT5 | -6.6604 | 7.29E-10 | 0.2583 | 1.59E-05 |
| rs7671868 | chr4 | 57256201 | PPAT | -6.6519 | 7.60E-10 | 0.2680 | 1.65E-05 |
| rs162035 | chr5 | 7883188 | MTRR | 6.6482 | 7.75E-10 | 0.2509 | 1.67E-05 |
| rs2064511 | chr20 | 34448810 | CPNE1 | -6.6259 | 8.67E-10 | 0.2992 | 1.86E-05 |
| rs682830 | chr1 | 89558103 | GBP3 | -6.6198 | 8.94E-10 | 0.2513 | 1.91E-05 |
| rs1667353 | chr19 | 37481153 | ZNF420 | 6.6087 | 9.46E-10 | 0.2515 | 2.02E-05 |
| rs5760201 | chr22 | 24474704 | SUSD2 | 6.5996 | 9.90E-10 | 0.2495 | 2.10E-05 |
| rs2033870 | chr2 | 190603830 | ORMDL1 | 6.5965 | 1.01E-09 | 0.2480 | 2.12E-05 |
| rs2026043 | chr1 | 89575677 | GBP3 | -6.5874 | 1.05E-09 | 0.2516 | 2.21E-05 |
| rs891159 | chr5 | 81491087 | RPS23 | -6.5823 | 1.08E-09 | 0.2572 | 2.26E-05 |
| rs211652 | chr23 | 2834840 | ARSD | 6.5772 | 1.11E-09 | 0.2509 | 2.31E-05 |
| rs324914 | chr5 | 81389950 | RPS23 | -6.5760 | 1.11E-09 | 0.2527 | 2.32E-05 |
| rs2232559 | chr12 | 53040411 | KRT1 | -6.5721 | 1.14E-09 | 0.2535 | 2.35E-05 |
| rs12108833 | chr5 | 81371755 | RPS23 | -6.5709 | 1.14E-09 | 0.2571 | 2.36E-05 |
| rs523979 | chr19 | 37456405 | ZNF420 | 6.5560 | 1.23E-09 | 0.2474 | 2.53E-05 |
| rs1136647 | chr7 | 73280020 | WBSCR27 | -6.5522 | 1.26E-09 | 0.2483 | 2.57E-05 |
| rs4623941 | chr12 | 75767341 | GLIPR1L2 | -6.5392 | 1.34E-09 | 0.2625 | 2.73E-05 |
| rs2001106 | chr22 | 24535229 | SUSD2 | 6.5289 | 1.41E-09 | 0.2679 | 2.86E-05 |
| rs6060692 | chr20 | 34517925 | CPNE1 | 6.5253 | 1.44E-09 | 0.2548 | 2.90E-05 |
| rs676913 | chr1 | 89637578 | GBP3 | -6.5222 | 1.46E-09 | 0.2470 | 2.94E-05 |
| rs17412368 | chr1 | 65939158 | LEPROT | -6.5204 | 1.47E-09 | 0.2453 | 2.95E-05 |
| rs2025805 | chr1 | 65949878 | LEPROT | -6.5194 | 1.48E-09 | 0.2439 | 2.95E-05 |
| rs1236904 | chr10 | 71267849 | TSPAN15 | 6.5184 | 1.49E-09 | 0.2664 | 2.96E-05 |
| rs1437891 | chr2 | 190562708 | ORMDL1 | 6.5155 | 1.51E-09 | 0.2433 | 2.98E-05 |
| rs1806261 | chr17 | 5317008 | RABEP1 | -6.5152 | 1.51E-09 | 0.3156 | 2.98E-05 |
| rs10775621 | chr19 | 16057677 | CYP4F11 | 6.5070 | 1.57E-09 | 0.2644 | 3.09E-05 |
| rs12155080 | chr7 | 128658739 | IRF5 | 6.5058 | 1.58E-09 | 0.2434 | 3.09E-05 |
| rs10971957 | chr9 | 34151463 | NUDT2 | -6.5056 | 1.59E-09 | 0.2818 | 3.09E-05 |
| rs13320 | chr1 | 151846517 | THEM4 | 6.4967 | 1.66E-09 | 0.2585 | 3.22E-05 |
| rs6666579 | chr1 | 113132393 | ST7L | 6.4918 | 1.70E-09 | 0.2599 | 3.29E-05 |
| rs862242 | chr5 | 81587566 | RPS23 | -6.4833 | 1.77E-09 | 0.2643 | 3.42E-05 |
| rs2432540 | chr16 | 56419628 | AMFR | 6.4687 | 1.91E-09 | 0.2408 | 3.66E-05 |
| rs2999544 | chr1 | 151903634 | THEM4 | 6.4521 | 2.07E-09 | 0.2398 | 3.96E-05 |
| rs9529314 | chr13 | 31799646 | B3GALTL | 6.4504 | 2.09E-09 | 0.2462 | 3.98E-05 |
| rs6558089 | chr8 | 28894237 | HMBOX1 | -6.4497 | 2.10E-09 | 0.2685 | 3.98E-05 |
| rs2966205 | chr16 | 56446612 | AMFR | 6.4319 | 2.29E-09 | 0.2384 | 4.30E-05 |
| rs17412723 | chr1 | 65952741 | LEPROT | -6.4314 | 2.30E-09 | 0.2509 | 4.30E-05 |
| rs2587871 | chr16 | 56412526 | AMFR | 6.4303 | 2.31E-09 | 0.2383 | 4.30E-05 |
| rs2587873 | chr16 | 56412734 | AMFR | 6.4303 | 2.31E-09 | 0.2383 | 4.30E-05 |
| rs6499837 | chr16 | 56453981 | AMFR | 6.4303 | 2.31E-09 | 0.2383 | 4.30E-05 |
| rs9937444 | chr16 | 56543558 | AMFR | 6.4262 | 2.36E-09 | 0.2381 | 4.37E-05 |
| rs459955 | chr20 | 44378059 | WFDC3 | -6.4150 | 2.49E-09 | 0.2534 | 4.61E-05 |
| rs1893596 | chr21 | 44236697 | WDR4 | -6.3996 | 2.69E-09 | 0.2448 | 4.95E-05 |
| rs4065552 | chr13 | 31850989 | B3GALTL | 6.3973 | 2.72E-09 | 0.2429 | 4.99E-05 |
| rs17004848 | chr22 | 24538918 | SUSD2 | 6.3922 | 2.79E-09 | 0.2412 | 5.10E-05 |
| rs863214 | chr5 | 79984714 | DHFR | -6.3845 | 2.90E-09 | 0.2609 | 5.28E-05 |
| rs4683294 | chr3 | 46979013 | CCDC12 | 6.3815 | 2.94E-09 | 0.2413 | 5.34E-05 |
| rs9543430 | chr13 | 31853948 | B3GALTL | 6.3803 | 2.96E-09 | 0.2898 | 5.35E-05 |
| rs1667354 | chr19 | 37482151 | ZNF420 | -6.3758 | 3.02E-09 | 0.2477 | 5.45E-05 |
| rs7557519 | chr2 | 190583187 | ORMDL1 | 6.3745 | 3.04E-09 | 0.2350 | 5.47E-05 |
| rs390409 | chr20 | 44381615 | WFDC3 | -6.3663 | 3.17E-09 | 0.2548 | 5.67E-05 |
| rs6088887 | chr20 | 34171324 | CPNE1 | 6.3645 | 3.20E-09 | 0.2411 | 5.70E-05 |
| rs2025803 | chr1 | 65946506 | LEPROT | -6.3629 | 3.22E-09 | 0.2432 | 5.73E-05 |
| rs826305 | chr19 | 37443625 | ZNF420 | 6.3612 | 3.25E-09 | 0.2663 | 5.76E-05 |
| rs586609 | chr1 | 89615979 | GBP3 | -6.3597 | 3.28E-09 | 0.2376 | 5.78E-05 |
| rs6834736 | chr4 | 57194525 | PPAT | -6.3577 | 3.31E-09 | 0.2415 | 5.82E-05 |
| rs2440467 | chr16 | 56417047 | AMFR | 6.3440 | 3.54E-09 | 0.2348 | 6.20E-05 |
| rs3741496 | chr12 | 75891253 | GLIPR1L2 | -6.3384 | 3.64E-09 | 0.2367 | 6.36E-05 |
| rs2999948 | chr9 | 85590972 | RASEF | 6.3371 | 3.66E-09 | 0.2362 | 6.37E-05 |
| rs5743063 | chr2 | 190694449 | ORMDL1 | 6.3350 | 3.70E-09 | 0.2329 | 6.42E-05 |
| rs8140812 | chr22 | 24553535 | SUSD2 | 6.3247 | 3.89E-09 | 0.2457 | 6.73E-05 |
| rs4655904 | chr1 | 89608576 | GBP3 | -6.3139 | 4.11E-09 | 0.2456 | 7.07E-05 |
| rs10161146 | chr12 | 96323420 | AMDHD1 | -6.3133 | 4.12E-09 | 0.2426 | 7.07E-05 |
| rs7486080 | chr12 | 96316796 | AMDHD1 | 6.3116 | 4.15E-09 | 0.2409 | 7.10E-05 |
| rs4755721 | chr11 | 43636378 | HSD17B12 | 6.3102 | 4.18E-09 | 0.2558 | 7.12E-05 |
| rs211653 | chr23 | 2836037 | ARSD | -6.3097 | 4.19E-09 | 0.2341 | 7.12E-05 |
| rs4731541 | chr7 | 128678236 | IRF5 | 6.2902 | 4.62E-09 | 0.2309 | 7.82E-05 |
| rs7969930 | chr12 | 75867565 | GLIPR1L2 | -6.2865 | 4.70E-09 | 0.2316 | 7.93E-05 |
| rs7524834 | chr1 | 65950230 | LEPROT | -6.2727 | 5.03E-09 | 0.2302 | 8.46E-05 |
| rs162445 | chr5 | 7943246 | MTRR | 6.2720 | 5.05E-09 | 0.2291 | 8.46E-05 |
| rs2507909 | chr11 | 115010867 | CADM1 | -6.2580 | 5.41E-09 | 0.2310 | 9.02E-05 |
| rs7263536 | chr20 | 34647457 | CPNE1 | 6.2577 | 5.41E-09 | 0.2411 | 9.02E-05 |
| rs2897554 | chr5 | 81276241 | RPS23 | -6.2569 | 5.43E-09 | 0.2900 | 9.02E-05 |
| rs910697 | chr1 | 113063125 | ST7L | -6.2479 | 5.68E-09 | 0.2332 | 9.40E-05 |
| rs3026109 | chr17 | 5284480 | RABEP1 | -6.2445 | 5.77E-09 | 0.3057 | 9.52E-05 |
| rs426521 | chr4 | 130035755 | C4orf33 | 6.2435 | 5.80E-09 | 0.2275 | 9.54E-05 |
| rs16842071 | chr2 | 201931730 | PPIL3 | -6.2350 | 6.05E-09 | 0.2400 | 9.91E-05 |
| rs2999526 | chr1 | 151951695 | THEM4 | 6.2271 | 6.29E-09 | 0.2265 | 0.0001 |
| rs2309399 | chr17 | 5391690 | RABEP1 | 6.2192 | 6.54E-09 | 0.2594 | 0.0001 |
| rs484361 | chr1 | 89689917 | GBP3 | -6.2148 | 6.68E-09 | 0.2258 | 0.0001 |
| rs1401907 | chr4 | 57187197 | PPAT | -6.2039 | 7.04E-09 | 0.2665 | 0.0001 |
| rs1043476 | chr1 | 118066459 | MAN1A2 | 6.2025 | 7.09E-09 | 0.2355 | 0.0001 |
| rs2548554 | chr5 | 10247010 | CCT5 | -6.2009 | 7.15E-09 | 0.2337 | 0.0001 |
| rs6142495 | chr20 | 34635221 | CPNE1 | 6.1912 | 7.50E-09 | 0.2284 | 0.0001 |
| rs6602130 | chr10 | 16558290 | PTER | 6.1876 | 7.63E-09 | 0.2782 | 0.0001 |
| rs9326953 | chr5 | 114549087 | PGGT1B | -6.1777 | 8.01E-09 | 0.2289 | 0.0001 |
| rs2664529 | chr20 | 44402869 | WFDC3 | -6.1767 | 8.05E-09 | 0.2449 | 0.0001 |
| rs7541758 | chr1 | 233522384 | KIAA1804 | 6.1726 | 8.21E-09 | 0.3103 | 0.0001 |
| rs719125 | chr2 | 202017860 | PPIL3 | -6.1706 | 8.29E-09 | 0.2293 | 0.0001 |
| rs4783941 | chr16 | 56511736 | AMFR | 6.1667 | 8.45E-09 | 0.2245 | 0.0001 |
| rs947403 | chr10 | 1016601 | IDI2 | 6.1591 | 8.77E-09 | 0.2225 | 0.0001 |
| rs2548552 | chr5 | 10248175 | CCT5 | -6.1553 | 8.93E-09 | 0.2343 | 0.0001 |
| rs12523353 | chr5 | 81546918 | RPS23 | 6.1523 | 9.06E-09 | 0.2222 | 0.0001 |
| rs761825 | chr20 | 34194054 | CPNE1 | -6.1466 | 9.31E-09 | 0.2608 | 0.0001 |
| rs6468685 | chr8 | 86918562 | CNGB3 | -6.1445 | 9.41E-09 | 0.3001 | 0.0001 |
| rs1757925 | chr4 | 130020791 | C4orf33 | 6.1403 | 9.60E-09 | 0.2230 | 0.0001 |
| rs1030831 | chr4 | 130025037 | C4orf33 | 6.1403 | 9.60E-09 | 0.2230 | 0.0001 |
| rs474794 | chr4 | 130038674 | C4orf33 | 6.1403 | 9.60E-09 | 0.2230 | 0.0001 |
| rs1685195 | chr20 | 44359847 | WFDC3 | -6.1380 | 9.71E-09 | 0.2631 | 0.0001 |
| rs12337340 | chr9 | 116328950 | C9orf43 | -6.1372 | 9.75E-09 | 0.2371 | 0.0001 |
| rs6088971 | chr20 | 34535373 | CPNE1 | 6.1370 | 9.76E-09 | 0.2336 | 0.0001 |
| rs9436747 | chr1 | 65911607 | LEPROT | 6.1327 | 9.97E-09 | 0.2302 | 0.0002 |
| rs11180548 | chr12 | 75880006 | GLIPR1L2 | -6.1110 | 1.11E-08 | 0.2224 | 0.0002 |
| rs2507904 | chr11 | 115001610 | CADM1 | -6.1067 | 1.13E-08 | 0.2233 | 0.0002 |
| rs10036937 | chr5 | 81555167 | RPS23 | 6.1020 | 1.16E-08 | 0.2207 | 0.0002 |
| rs1864181 | chr5 | 81549280 | RPS23 | 6.0977 | 1.18E-08 | 0.2208 | 0.0002 |
| rs11162072 | chr1 | 76510231 | ST6GALNAC3 | 6.0900 | 1.23E-08 | 0.2302 | 0.0002 |
| rs1316326 | chr1 | 117980755 | MAN1A2 | 6.0869 | 1.24E-08 | 0.2194 | 0.0002 |
| rs6966355 | chr7 | 42464928 | HECW1 | -6.0808 | 1.28E-08 | 0.2186 | 0.0002 |
| rs10065463 | chr5 | 81500280 | RPS23 | 6.0804 | 1.28E-08 | 0.2242 | 0.0002 |
| rs11671591 | chr19 | 58918337 | ZNF584 | -6.0790 | 1.29E-08 | 0.2266 | 0.0002 |
| rs478671 | chr1 | 89559560 | GBP3 | -6.0777 | 1.30E-08 | 0.2303 | 0.0002 |
| rs4565940 | chr12 | 75812296 | GLIPR1L2 | -6.0754 | 1.32E-08 | 0.2306 | 0.0002 |
| rs2741158 | chr12 | 53071560 | KRT1 | -6.0673 | 1.37E-08 | 0.2434 | 0.0002 |
| rs13427243 | chr2 | 69690187 | GFPT1 | -6.0558 | 1.45E-08 | 0.2186 | 0.0002 |
| rs7453 | chr20 | 25207074 | ABHD12 | -6.0502 | 1.49E-08 | 0.2461 | 0.0002 |
| rs4666783 | chr2 | 190612980 | ORMDL1 | 6.0501 | 1.49E-08 | 0.2673 | 0.0002 |
| rs7974011 | chr12 | 75871753 | GLIPR1L2 | -6.0463 | 1.51E-08 | 0.2174 | 0.0002 |
| rs9540737 | chr13 | 31780988 | B3GALTL | 6.0430 | 1.54E-08 | 0.2527 | 0.0002 |
| rs17419290 | chr5 | 60307904 | ERCC8 | 6.0421 | 1.55E-08 | 0.2287 | 0.0002 |
| rs4732893 | chr8 | 28794678 | HMBOX1 | -6.0415 | 1.55E-08 | 0.2460 | 0.0002 |
| rs389376 | chr4 | 130036179 | C4orf33 | 6.0415 | 1.55E-08 | 0.2334 | 0.0002 |
| rs10405214 | chr19 | 52032532 | SIGLEC12 | 6.0398 | 1.56E-08 | 0.2196 | 0.0002 |
| rs4487072 | chr2 | 202008298 | PPIL3 | -6.0379 | 1.58E-08 | 0.2302 | 0.0002 |
| rs1502684 | chr9 | 85601000 | RASEF | 6.0333 | 1.61E-08 | 0.3314 | 0.0002 |
| rs2279502 | chr1 | 151917652 | THEM4 | 6.0293 | 1.64E-08 | 0.2227 | 0.0002 |
| rs11690738 | chr2 | 69530084 | GFPT1 | -6.0280 | 1.65E-08 | 0.2191 | 0.0002 |
| rs7267979 | chr20 | 25298087 | ABHD12 | 6.0276 | 1.66E-08 | 0.2345 | 0.0002 |
| rs1709419 | chr4 | 130038913 | C4orf33 | 6.0249 | 1.68E-08 | 0.2181 | 0.0002 |
| rs6582289 | chr12 | 75820605 | GLIPR1L2 | -6.0235 | 1.69E-08 | 0.2171 | 0.0002 |
| rs11209 | chr17 | 5289580 | RABEP1 | -6.0203 | 1.72E-08 | 0.2625 | 0.0002 |
| rs2693 | chr8 | 28924858 | HMBOX1 | -6.0183 | 1.73E-08 | 0.2455 | 0.0002 |
| rs8141797 | chr22 | 24582041 | SUSD2 | 6.0042 | 1.85E-08 | 0.2270 | 0.0003 |
| rs7852399 | chr9 | 34371788 | NUDT2 | 6.0021 | 1.87E-08 | 0.2141 | 0.0003 |
| rs12286929 | chr11 | 115022404 | CADM1 | -6.0012 | 1.88E-08 | 0.2135 | 0.0003 |
| rs6674384 | chr1 | 114455907 | DCLRE1B | 6.0002 | 1.89E-08 | 0.2214 | 0.0003 |
| rs10167387 | chr2 | 201808618 | PPIL3 | -5.9935 | 1.95E-08 | 0.2208 | 0.0003 |
| rs1909968 | chr12 | 75811011 | GLIPR1L2 | -5.9870 | 2.01E-08 | 0.2331 | 0.0003 |
| rs4394867 | chr12 | 75811827 | GLIPR1L2 | -5.9836 | 2.05E-08 | 0.2145 | 0.0003 |
| rs457570 | chr20 | 44371922 | WFDC3 | -5.9827 | 2.06E-08 | 0.2235 | 0.0003 |
| rs4762254 | chr12 | 96330581 | AMDHD1 | -5.9821 | 2.06E-08 | 0.2163 | 0.0003 |
| rs1487969 | chr8 | 28885661 | HMBOX1 | -5.9776 | 2.11E-08 | 0.2579 | 0.0003 |
| rs11670871 | chr19 | 58940514 | ZNF584 | -5.9766 | 2.12E-08 | 0.2257 | 0.0003 |
| rs459681 | chr20 | 44372005 | WFDC3 | -5.9721 | 2.16E-08 | 0.2317 | 0.0003 |
| rs7513047 | chr1 | 65950340 | LEPROT | -5.9620 | 2.27E-08 | 0.2179 | 0.0003 |
| rs4357190 | chr7 | 837407 | PRKAR1B | -5.9545 | 2.35E-08 | 0.2203 | 0.0003 |
| rs4019400 | chr12 | 75809758 | GLIPR1L2 | -5.9480 | 2.43E-08 | 0.2153 | 0.0003 |
| rs370151 | chr16 | 56561494 | AMFR | 5.9474 | 2.43E-08 | 0.2104 | 0.0003 |
| rs390260 | chr4 | 130036591 | C4orf33 | 5.9431 | 2.49E-08 | 0.2149 | 0.0003 |
| rs17713729 | chr2 | 249092 | SH3YL1 | -5.9341 | 2.60E-08 | 0.2154 | 0.0003 |
| rs711433 | chr7 | 105153191 | RINT1 | -5.9337 | 2.60E-08 | 0.2099 | 0.0003 |
| rs4815412 | chr20 | 25350325 | ABHD12 | 5.9257 | 2.70E-08 | 0.2192 | 0.0004 |
| rs7370877 | chr2 | 69525721 | GFPT1 | -5.9226 | 2.74E-08 | 0.2121 | 0.0004 |
| rs11668252 | chr19 | 16072153 | CYP4F11 | 5.9201 | 2.77E-08 | 0.2088 | 0.0004 |
| rs1019943 | chr19 | 16069161 | CYP4F11 | 5.9143 | 2.85E-08 | 0.2114 | 0.0004 |
| rs509303 | chr11 | 65093190 | DPF2 | -5.9143 | 2.85E-08 | 0.2100 | 0.0004 |
| rs17097933 | chr1 | 76492130 | ST6GALNAC3 | 5.9092 | 2.92E-08 | 0.2180 | 0.0004 |
| rs4592895 | chr2 | 69567984 | GFPT1 | -5.9073 | 2.95E-08 | 0.2081 | 0.0004 |
| rs10879913 | chr12 | 75809070 | GLIPR1L2 | -5.9054 | 2.98E-08 | 0.2176 | 0.0004 |
| rs2999531 | chr1 | 151971497 | THEM4 | 5.9030 | 3.01E-08 | 0.2156 | 0.0004 |
| rs1550388 | chr2 | 190603780 | ORMDL1 | 5.9013 | 3.04E-08 | 0.2299 | 0.0004 |
| rs7258360 | chr19 | 37605917 | ZNF420 | 5.8947 | 3.13E-08 | 0.2584 | 0.0004 |
| rs7135440 | chr12 | 75796721 | GLIPR1L2 | -5.8916 | 3.18E-08 | 0.2094 | 0.0004 |
| rs674478 | chr22 | 21021264 | SNAP29 | -5.8902 | 3.20E-08 | 0.2071 | 0.0004 |
| rs2132173 | chr3 | 47247060 | CCDC12 | -5.8856 | 3.27E-08 | 0.2089 | 0.0004 |
| rs10170036 | chr2 | 69623136 | GFPT1 | -5.8791 | 3.37E-08 | 0.2090 | 0.0004 |
| rs2061197 | chr3 | 47001350 | CCDC12 | -5.8567 | 3.75E-08 | 0.2061 | 0.0005 |
| rs1888999 | chr20 | 25291848 | ABHD12 | 5.8541 | 3.80E-08 | 0.2222 | 0.0005 |
| rs1898560 | chr2 | 190612327 | ORMDL1 | 5.8458 | 3.95E-08 | 0.2275 | 0.0005 |
| rs2474777 | chr20 | 25272633 | ABHD12 | 5.8385 | 4.09E-08 | 0.2437 | 0.0005 |
| rs10474690 | chr5 | 1374544 | IRX4 | 5.8381 | 4.10E-08 | 0.2236 | 0.0005 |
| rs2960839 | chr7 | 1204295 | ZFAND2A | 5.8381 | 4.10E-08 | 0.2232 | 0.0005 |
| rs175057 | chr14 | 75489632 | MLH3 | -5.8354 | 4.15E-08 | 0.2502 | 0.0005 |
| rs1511575 | chr13 | 31816524 | B3GALTL | 5.8339 | 4.18E-08 | 0.2253 | 0.0005 |
| rs9303283 | chr17 | 38192633 | RPL19 | 5.8336 | 4.19E-08 | 0.4174 | 0.0005 |
| rs1051446 | chr12 | 75894114 | GLIPR1L2 | -5.8329 | 4.20E-08 | 0.2215 | 0.0005 |
| rs7125361 | chr11 | 115080042 | CADM1 | -5.8316 | 4.22E-08 | 0.2037 | 0.0005 |
| rs6132819 | chr20 | 25218618 | ABHD12 | 5.8314 | 4.23E-08 | 0.2214 | 0.0005 |
| rs6050573 | chr20 | 25366065 | ABHD12 | 5.8237 | 4.39E-08 | 0.2295 | 0.0005 |
| rs8184820 | chr20 | 25250577 | ABHD12 | 5.8230 | 4.40E-08 | 0.2164 | 0.0005 |
| rs660768 | chr1 | 89701383 | GBP3 | -5.8188 | 4.49E-08 | 0.2139 | 0.0005 |
| rs4882618 | chr12 | 75776705 | GLIPR1L2 | -5.8176 | 4.52E-08 | 0.2071 | 0.0006 |
| rs1715450 | chr11 | 118128445 | MPZL3 | 5.8172 | 4.52E-08 | 0.2153 | 0.0006 |
| rs9514058 | chr13 | 103454369 | BIVM | 5.8163 | 4.54E-08 | 0.2161 | 0.0006 |
| rs11949767 | chr5 | 176740244 | PRELID1 | -5.8117 | 4.64E-08 | 0.2299 | 0.0006 |
| rs2272347 | chr7 | 128619415 | IRF5 | 5.8117 | 4.64E-08 | 0.2143 | 0.0006 |
| rs4984902 | chr16 | 655262 | WFIKKN1 | 5.8112 | 4.65E-08 | 0.2025 | 0.0006 |
| rs9557936 | chr13 | 103473497 | BIVM | 5.8103 | 4.67E-08 | 0.2190 | 0.0006 |
| rs2258719 | chr20 | 25275843 | ABHD12 | 5.8097 | 4.69E-08 | 0.2211 | 0.0006 |
| rs12369556 | chr12 | 75773464 | GLIPR1L2 | -5.8096 | 4.69E-08 | 0.2082 | 0.0006 |
| rs7955412 | chr12 | 6998371 | LRRC23 | 5.8036 | 4.82E-08 | 0.2204 | 0.0006 |
| rs2108978 | chr17 | 19861458 | AKAP10 | 5.8010 | 4.88E-08 | 0.2511 | 0.0006 |
| rs2155574 | chr11 | 96113441 | CCDC82 | 5.7951 | 5.02E-08 | 0.2034 | 0.0006 |
| rs9917851 | chr4 | 130192103 | C4orf33 | 5.7938 | 5.05E-08 | 0.2390 | 0.0006 |
| rs2289226 | chr2 | 190526205 | ORMDL1 | 5.7914 | 5.11E-08 | 0.2150 | 0.0006 |
| rs10879912 | chr12 | 75808957 | GLIPR1L2 | -5.7874 | 5.21E-08 | 0.2020 | 0.0006 |
| rs1329118 | chr1 | 89565519 | GBP3 | -5.7807 | 5.37E-08 | 0.2203 | 0.0006 |
| rs467129 | chr20 | 44371905 | WFDC3 | -5.7758 | 5.50E-08 | 0.2185 | 0.0006 |
| rs10091515 | chr8 | 28958517 | HMBOX1 | -5.7660 | 5.76E-08 | 0.2087 | 0.0007 |
| rs17332824 | chr5 | 60166187 | ERCC8 | 5.7643 | 5.81E-08 | 0.2234 | 0.0007 |
| rs3746337 | chr20 | 25208272 | ABHD12 | -5.7627 | 5.85E-08 | 0.2197 | 0.0007 |
| rs5757187 | chr22 | 39021522 | CBY1 | 5.7572 | 6.00E-08 | 0.2072 | 0.0007 |
| rs2202104 | chr12 | 75807692 | GLIPR1L2 | -5.7568 | 6.01E-08 | 0.2044 | 0.0007 |
| rs5765304 | chr22 | 45771974 | FAM118A | 5.7565 | 6.02E-08 | 0.3635 | 0.0007 |
| rs6050598 | chr20 | 25397257 | ABHD12 | 5.7549 | 6.07E-08 | 0.2213 | 0.0007 |
| rs591163 | chr11 | 126148432 | FOXRED1 | -5.7530 | 6.12E-08 | 0.1993 | 0.0007 |
| rs878129 | chr1 | 114459195 | DCLRE1B | 5.7498 | 6.21E-08 | 0.2005 | 0.0007 |
| rs17247678 | chr5 | 81652773 | RPS23 | -5.7492 | 6.23E-08 | 0.1997 | 0.0007 |
| rs2932590 | chr1 | 151953763 | THEM4 | 5.7491 | 6.23E-08 | 0.2034 | 0.0007 |
| rs6083862 | chr20 | 25411086 | ABHD12 | 5.7462 | 6.32E-08 | 0.2271 | 0.0007 |
| rs2084385 | chr3 | 196553867 | PAK2 | 5.7458 | 6.33E-08 | 0.2237 | 0.0007 |
| rs7535776 | chr1 | 76530627 | ST6GALNAC3 | 5.7417 | 6.46E-08 | 0.1986 | 0.0007 |
| rs6504177 | chr17 | 61812655 | FTSJ3 | -5.7401 | 6.50E-08 | 0.2216 | 0.0007 |
| rs12416136 | chr10 | 88713604 | BMPR1A | -5.7358 | 6.64E-08 | 0.2081 | 0.0008 |
| rs11871517 | chr17 | 5249998 | RABEP1 | 5.7332 | 6.72E-08 | 0.2496 | 0.0008 |
| rs11599792 | chr10 | 1017914 | IDI2 | 5.7330 | 6.72E-08 | 0.2000 | 0.0008 |
| rs7975661 | chr12 | 75696430 | GLIPR1L2 | -5.7302 | 6.81E-08 | 0.2005 | 0.0008 |
| rs1344237 | chr2 | 171984435 | TLK1 | 5.7291 | 6.85E-08 | 0.1979 | 0.0008 |
| rs2297497 | chr20 | 25297909 | ABHD12 | 5.7249 | 6.98E-08 | 0.2134 | 0.0008 |
| rs10171850 | chr2 | 172006741 | TLK1 | 5.7231 | 7.04E-08 | 0.1975 | 0.0008 |
| rs1644673 | chr19 | 37478711 | ZNF420 | 5.7227 | 7.06E-08 | 0.2123 | 0.0008 |
| rs1699387 | chr4 | 130031498 | C4orf33 | 5.7141 | 7.35E-08 | 0.2034 | 0.0008 |
| rs1981726 | chr2 | 202008700 | CFLAR | -5.7139 | 7.35E-08 | 0.2109 | 0.0008 |
| rs9534966 | chr13 | 48711678 | MED4 | -5.7074 | 7.58E-08 | 0.1980 | 0.0008 |
| rs2168443 | chr3 | 46947087 | CCDC12 | 5.7072 | 7.59E-08 | 0.1989 | 0.0008 |
| rs12477649 | chr2 | 171962676 | TLK1 | 5.7053 | 7.66E-08 | 0.1971 | 0.0008 |
| rs223413 | chr4 | 103732866 | MANBA | 5.7050 | 7.67E-08 | 0.2059 | 0.0008 |
| rs4911509 | chr20 | 34087309 | CPNE1 | -5.7029 | 7.74E-08 | 0.1988 | 0.0009 |
| rs153060 | chr16 | 69377553 | TMED6 | -5.7021 | 7.77E-08 | 0.1963 | 0.0009 |
| rs1569492 | chr22 | 38975511 | CBY1 | -5.7016 | 7.79E-08 | 0.2078 | 0.0009 |
| rs12342699 | chr9 | 116282114 | C9orf43 | -5.6973 | 7.95E-08 | 0.1987 | 0.0009 |
| rs5757193 | chr22 | 39034867 | CBY1 | 5.6936 | 8.09E-08 | 0.1972 | 0.0009 |
| rs6883052 | chr5 | 88674689 | MEF2C | 5.6936 | 8.09E-08 | 0.2001 | 0.0009 |
| rs1401904 | chr4 | 57187665 | PPAT | -5.6930 | 8.11E-08 | 0.2111 | 0.0009 |
| rs7853609 | chr9 | 34320195 | NUDT2 | 5.6911 | 8.18E-08 | 0.1967 | 0.0009 |
| rs7099607 | chr10 | 1048343 | IDI2 | 5.6865 | 8.36E-08 | 0.2009 | 0.0009 |
| rs9304718 | chr19 | 52011930 | SIGLEC12 | -5.6865 | 8.36E-08 | 0.1974 | 0.0009 |
| rs6546505 | chr2 | 69586293 | GFPT1 | -5.6858 | 8.39E-08 | 0.2024 | 0.0009 |
| rs1806263 | chr17 | 5317492 | RABEP1 | -5.6841 | 8.45E-08 | 0.2352 | 0.0009 |
| rs638766 | chr11 | 126134698 | FOXRED1 | -5.6831 | 8.49E-08 | 0.1952 | 0.0009 |
| rs2285897 | chr19 | 987571 | WDR18 | 5.6828 | 8.50E-08 | 0.1953 | 0.0009 |
| rs2050865 | chr9 | 116277388 | C9orf43 | 5.6812 | 8.57E-08 | 0.1958 | 0.0009 |
| rs17037333 | chr1 | 118032157 | MAN1A2 | 5.6775 | 8.72E-08 | 0.2329 | 0.0009 |
| rs5750672 | chr22 | 39104168 | CBY1 | 5.6757 | 8.79E-08 | 0.1998 | 0.0009 |
| rs4802807 | chr19 | 52030448 | SIGLEC12 | -5.6744 | 8.84E-08 | 0.1952 | 0.0009 |
| rs1981726 | chr2 | 202008700 | PPIL3 | -5.6741 | 8.86E-08 | 0.2205 | 0.0009 |
| rs457153 | chr20 | 44361491 | WFDC3 | -5.6725 | 8.92E-08 | 0.2115 | 0.0009 |
| rs6433276 | chr2 | 171867383 | TLK1 | 5.6677 | 9.13E-08 | 0.1944 | 0.0010 |
| rs1025240 | chr2 | 171958801 | TLK1 | 5.6677 | 9.13E-08 | 0.1944 | 0.0010 |
| rs7485085 | chr12 | 96355432 | AMDHD1 | -5.6675 | 9.13E-08 | 0.1946 | 0.0010 |
| rs587891 | chr11 | 126081336 | FOXRED1 | -5.6670 | 9.16E-08 | 0.1966 | 0.0010 |
| rs203457 | chr17 | 19818338 | AKAP10 | 5.6649 | 9.25E-08 | 0.2100 | 0.0010 |
| rs7577689 | chr2 | 69686286 | GFPT1 | -5.6610 | 9.42E-08 | 0.1987 | 0.0010 |
| rs11581176 | chr1 | 92982418 | EVI5 | 5.6599 | 9.46E-08 | 0.1962 | 0.0010 |
| rs2260997 | chr20 | 25256266 | ABHD12 | 5.6598 | 9.47E-08 | 0.2172 | 0.0010 |
| rs12581394 | chr12 | 75705581 | GLIPR1L2 | -5.6560 | 9.64E-08 | 0.1972 | 0.0010 |
| rs13029229 | chr2 | 61030742 | PAPOLG | 5.6526 | 9.79E-08 | 0.2002 | 0.0010 |
| rs4078089 | chr2 | 69605552 | GFPT1 | -5.6519 | 9.82E-08 | 0.1952 | 0.0010 |
| rs6442055 | chr3 | 47110722 | CCDC12 | -5.6506 | 9.88E-08 | 0.1965 | 0.0010 |
| rs6767907 | chr3 | 47162661 | CCDC12 | -5.6506 | 9.88E-08 | 0.1965 | 0.0010 |
| rs4878560 | chr9 | 34282388 | NUDT2 | 5.6485 | 9.98E-08 | 0.1936 | 0.0010 |
| rs2904980 | chr11 | 65001679 | DPF2 | -5.6455 | 1.01E-07 | 0.1946 | 0.0010 |
| rs6724567 | chr2 | 69585176 | GFPT1 | -5.6445 | 1.02E-07 | 0.1937 | 0.0010 |
| rs10776755 | chr1 | 113082974 | ST7L | 5.6404 | 1.04E-07 | 0.1986 | 0.0011 |
| rs1946990 | chr22 | 38912771 | CBY1 | -5.6386 | 1.05E-07 | 0.1954 | 0.0011 |
| rs9293290 | chr5 | 81474225 | RPS23 | 5.6356 | 1.06E-07 | 0.2093 | 0.0011 |
| rs8039354 | chr15 | 45551756 | SLC28A2 | -5.6349 | 1.06E-07 | 0.2017 | 0.0011 |
| rs573556 | chr17 | 37082081 | RPL19 | 5.6336 | 1.07E-07 | 0.2122 | 0.0011 |
| rs4484568 | chr7 | 73187651 | WBSCR27 | -5.6281 | 1.10E-07 | 0.2113 | 0.0011 |
| rs659551 | chr11 | 126076824 | FOXRED1 | -5.6281 | 1.10E-07 | 0.1954 | 0.0011 |
| rs6620640 | chr23 | 78915004 | TBX22 | 5.6235 | 1.12E-07 | 0.2086 | 0.0011 |
| rs11020505 | chr11 | 93478133 | C11orf54 | -5.6181 | 1.15E-07 | 0.2115 | 0.0012 |
| rs7580642 | chr2 | 69625638 | GFPT1 | -5.6127 | 1.18E-07 | 0.1988 | 0.0012 |
| rs10948723 | chr6 | 52666405 | GSTA3 | -5.6097 | 1.20E-07 | 0.1922 | 0.0012 |
| rs1612652 | chr19 | 37477640 | ZNF420 | 5.6073 | 1.21E-07 | 0.2154 | 0.0012 |
| rs611910 | chr11 | 65049278 | DPF2 | -5.6022 | 1.24E-07 | 0.1906 | 0.0012 |
| rs2665831 | chr17 | 61912392 | FTSJ3 | -5.6016 | 1.24E-07 | 0.1956 | 0.0012 |
| rs7314535 | chr12 | 7009208 | LRRC23 | 5.5977 | 1.26E-07 | 0.2352 | 0.0013 |
| rs6141601 | chr20 | 34716048 | CPNE1 | 5.5951 | 1.28E-07 | 0.1998 | 0.0013 |
| rs4149360 | chr6 | 3006807 | NQO2 | -5.5928 | 1.29E-07 | 0.2241 | 0.0013 |
| rs598816 | chr1 | 89642150 | GBP3 | -5.5926 | 1.29E-07 | 0.2173 | 0.0013 |
| rs2749029 | chr6 | 52631188 | GSTA3 | -5.5921 | 1.30E-07 | 0.1905 | 0.0013 |
| rs1005824 | chr13 | 31816016 | B3GALTL | 5.5898 | 1.31E-07 | 0.1909 | 0.0013 |
| rs3845960 | chr3 | 126246635 | CHST13 | -5.5869 | 1.33E-07 | 0.1954 | 0.0013 |
| rs8135371 | chr22 | 40757228 | DNAJB7 | 5.5799 | 1.37E-07 | 0.1998 | 0.0013 |
| rs4838959 | chr1 | 113085721 | ST7L | 5.5789 | 1.38E-07 | 0.1912 | 0.0013 |
| rs12612871 | chr2 | 171810732 | TLK1 | 5.5765 | 1.39E-07 | 0.1897 | 0.0014 |
| rs7605824 | chr2 | 280819 | SH3YL1 | -5.5754 | 1.40E-07 | 0.1936 | 0.0014 |
| rs6060673 | chr20 | 34482396 | CPNE1 | 5.5730 | 1.42E-07 | 0.1987 | 0.0014 |
| rs10515177 | chr17 | 58167485 | HEATR6 | 5.5718 | 1.42E-07 | 0.1964 | 0.0014 |
| rs2244468 | chr9 | 130215279 | RPL12 | 5.5711 | 1.43E-07 | 0.1939 | 0.0014 |
| rs6880798 | chr5 | 176704457 | PRELID1 | -5.5704 | 1.43E-07 | 0.2147 | 0.0014 |
| rs2009362 | chr19 | 52002729 | SIGLEC12 | -5.5690 | 1.44E-07 | 0.1953 | 0.0014 |
| rs10183486 | chr2 | 171990971 | TLK1 | 5.5689 | 1.44E-07 | 0.1887 | 0.0014 |
| rs2960841 | chr7 | 1204894 | ZFAND2A | 5.5669 | 1.46E-07 | 0.1889 | 0.0014 |
| rs2015353 | chr17 | 15873275 | ZSWIM7 | 5.5640 | 1.48E-07 | 0.2192 | 0.0014 |
| rs7559850 | chr2 | 69648520 | GFPT1 | -5.5638 | 1.48E-07 | 0.2131 | 0.0014 |
| rs2278963 | chr3 | 47054869 | CCDC12 | -5.5583 | 1.52E-07 | 0.1926 | 0.0014 |
| rs7875342 | chr9 | 34329110 | NUDT2 | 5.5581 | 1.52E-07 | 0.1929 | 0.0014 |
| rs12428 | chr20 | 25433821 | ABHD12 | 5.5565 | 1.53E-07 | 0.2306 | 0.0015 |
| rs7212681 | chr17 | 5191527 | RABEP1 | -5.5556 | 1.53E-07 | 0.2560 | 0.0015 |
| rs4487072 | chr2 | 202008298 | CFLAR | -5.5550 | 1.54E-07 | 0.1962 | 0.0015 |
| rs7808907 | chr7 | 128584084 | IRF5 | 5.5548 | 1.54E-07 | 0.1912 | 0.0015 |
| rs749511 | chr3 | 47035735 | CCDC12 | -5.5483 | 1.59E-07 | 0.1924 | 0.0015 |
| rs2271792 | chr17 | 75201529 | SEC14L1 | 5.5479 | 1.59E-07 | 0.1889 | 0.0015 |
| rs12086549 | chr1 | 119614337 | WARS2 | 5.5475 | 1.59E-07 | 0.1910 | 0.0015 |
| rs279543 | chr3 | 9935263 | JAGN1 | 5.5443 | 1.62E-07 | 0.1900 | 0.0015 |
| rs1467110 | chr11 | 64951427 | DPF2 | -5.5431 | 1.63E-07 | 0.1940 | 0.0015 |
| rs6001173 | chr22 | 39015302 | CBY1 | -5.5429 | 1.63E-07 | 0.1891 | 0.0015 |
| rs17790303 | chr5 | 81690042 | RPS23 | -5.5406 | 1.64E-07 | 0.2023 | 0.0015 |
| rs9655470 | chr7 | 1151713 | ZFAND2A | -5.5403 | 1.65E-07 | 0.1879 | 0.0015 |
| rs9655467 | chr7 | 1150542 | ZFAND2A | -5.5380 | 1.66E-07 | 0.1927 | 0.0015 |
| rs433352 | chr20 | 25467992 | ABHD12 | 5.5376 | 1.67E-07 | 0.2143 | 0.0015 |
| rs6032449 | chr20 | 44336716 | WFDC3 | -5.5361 | 1.68E-07 | 0.1870 | 0.0016 |
| rs6050565 | chr20 | 25347563 | ABHD12 | 5.5312 | 1.72E-07 | 0.2146 | 0.0016 |
| rs3785074 | chr16 | 69406986 | SNTB2 | 5.5308 | 1.72E-07 | 0.1976 | 0.0016 |
| rs399672 | chr20 | 44438299 | WFDC3 | -5.5284 | 1.74E-07 | 0.1912 | 0.0016 |
| rs162029 | chr5 | 7865527 | MTRR | 5.5254 | 1.76E-07 | 0.2445 | 0.0016 |
| rs2729371 | chr11 | 57321322 | TIMM10 | 5.5236 | 1.78E-07 | 0.1913 | 0.0016 |
| rs4732896 | chr8 | 28825474 | HMBOX1 | -5.5174 | 1.83E-07 | 0.2080 | 0.0017 |
| rs6958252 | chr7 | 105144984 | RINT1 | -5.5163 | 1.84E-07 | 0.1861 | 0.0017 |
| rs138703 | chr22 | 39132748 | CBY1 | -5.5159 | 1.84E-07 | 0.1871 | 0.0017 |
| rs9837343 | chr3 | 47177388 | CCDC12 | -5.5153 | 1.85E-07 | 0.1904 | 0.0017 |
| rs1375476 | chr19 | 37465175 | ZNF420 | 5.5152 | 1.85E-07 | 0.2675 | 0.0017 |
| rs10923767 | chr1 | 119732347 | WARS2 | 5.5140 | 1.86E-07 | 0.1909 | 0.0017 |
| rs2563335 | chr5 | 140044292 | ZMAT2 | -5.5133 | 1.86E-07 | 0.2183 | 0.0017 |
| rs630055 | chr11 | 65087533 | DPF2 | -5.5084 | 1.91E-07 | 0.1853 | 0.0017 |
| rs11214577 | chr11 | 113204266 | TTC12 | 5.5070 | 1.92E-07 | 0.1925 | 0.0017 |
| rs686305 | chr11 | 126187769 | FOXRED1 | -5.5030 | 1.95E-07 | 0.1892 | 0.0018 |
| rs376511 | chr3 | 9962816 | JAGN1 | -5.5015 | 1.97E-07 | 0.1959 | 0.0018 |
| rs10461018 | chr3 | 46995242 | CCDC12 | -5.4991 | 1.99E-07 | 0.2039 | 0.0018 |
| rs6537830 | chr1 | 110107044 | GNAI3 | -5.4969 | 2.01E-07 | 0.1949 | 0.0018 |
| rs3791220 | chr2 | 227066 | SH3YL1 | -5.4964 | 2.01E-07 | 0.1927 | 0.0018 |
| rs17713396 | chr2 | 227201 | SH3YL1 | -5.4964 | 2.01E-07 | 0.1927 | 0.0018 |
| rs7132019 | chr12 | 6992122 | LRRC23 | 5.4952 | 2.03E-07 | 0.2240 | 0.0018 |
| rs6491711 | chr13 | 103478475 | BIVM | 5.4946 | 2.03E-07 | 0.1926 | 0.0018 |
| rs12023963 | chr1 | 110084827 | GNAI3 | -5.4904 | 2.07E-07 | 0.1947 | 0.0018 |
| rs4815411 | chr20 | 25350223 | ABHD12 | 5.4827 | 2.15E-07 | 0.1990 | 0.0019 |
| rs10849538 | chr12 | 7014607 | LRRC23 | 5.4817 | 2.16E-07 | 0.2078 | 0.0019 |
| rs12151356 | chr19 | 36181046 | U2AF1L4 | -5.4815 | 2.16E-07 | 0.2245 | 0.0019 |
| rs2779212 | chr17 | 15876655 | ZSWIM7 | 5.4792 | 2.18E-07 | 0.2097 | 0.0019 |
| rs1995969 | chr13 | 31874444 | B3GALTL | 5.4772 | 2.20E-07 | 0.2379 | 0.0019 |
| rs6919 | chr17 | 61909485 | FTSJ3 | -5.4769 | 2.20E-07 | 0.1885 | 0.0019 |
| rs10221457 | chr19 | 52028950 | SIGLEC12 | 5.4749 | 2.22E-07 | 0.1859 | 0.0019 |
| rs7024612 | chr9 | 116321695 | C9orf43 | -5.4733 | 2.24E-07 | 0.2088 | 0.0020 |
| rs950546 | chr9 | 34310083 | NUDT2 | 5.4685 | 2.29E-07 | 0.1873 | 0.0020 |
| rs628506 | chr5 | 176534423 | PRELID1 | -5.4657 | 2.32E-07 | 0.1869 | 0.0020 |
| rs10008927 | chr4 | 129729239 | C4orf33 | 5.4655 | 2.32E-07 | 0.1896 | 0.0020 |
| rs7642 | chr2 | 69547236 | GFPT1 | -5.4654 | 2.32E-07 | 0.1875 | 0.0020 |
| rs6554348 | chr4 | 57219592 | PPAT | -5.4645 | 2.33E-07 | 0.2230 | 0.0020 |
| rs12044778 | chr1 | 110084408 | GNAI3 | -5.4592 | 2.39E-07 | 0.1928 | 0.0021 |
| rs3743669 | chr16 | 69398353 | SNTB2 | 5.4572 | 2.41E-07 | 0.1939 | 0.0021 |
| rs150543 | chr5 | 95037003 | SPATA9 | -5.4486 | 2.51E-07 | 0.1830 | 0.0022 |
| rs3746410 | chr20 | 34190870 | CPNE1 | 5.4463 | 2.53E-07 | 0.1865 | 0.0022 |
| rs239253 | chr11 | 64984743 | DPF2 | -5.4448 | 2.55E-07 | 0.1864 | 0.0022 |
| rs7264396 | chr20 | 34154741 | CPNE1 | 5.4402 | 2.61E-07 | 0.2035 | 0.0022 |
| rs2794311 | chr1 | 119595949 | WARS2 | 5.4357 | 2.66E-07 | 0.1846 | 0.0023 |
| rs10422359 | chr19 | 52047077 | SIGLEC12 | -5.4340 | 2.68E-07 | 0.1811 | 0.0023 |
| rs4941618 | chr13 | 48517289 | MED4 | -5.4340 | 2.68E-07 | 0.1811 | 0.0023 |
| rs7028327 | chr9 | 116264616 | C9orf43 | -5.4333 | 2.69E-07 | 0.1811 | 0.0023 |
| rs7872283 | chr9 | 116265088 | C9orf43 | -5.4333 | 2.69E-07 | 0.1811 | 0.0023 |
| rs10981803 | chr9 | 116277963 | C9orf43 | -5.4333 | 2.69E-07 | 0.1811 | 0.0023 |
| rs7863159 | chr9 | 116336574 | C9orf43 | -5.4333 | 2.69E-07 | 0.1811 | 0.0023 |
| rs2275997 | chr11 | 115100186 | CADM1 | -5.4330 | 2.69E-07 | 0.1862 | 0.0023 |
| rs719125 | chr2 | 202017860 | CFLAR | -5.4305 | 2.72E-07 | 0.1811 | 0.0023 |
| rs4814749 | chr20 | 18418332 | DTD1 | -5.4304 | 2.72E-07 | 0.1949 | 0.0023 |
| rs2267390 | chr22 | 38889657 | CBY1 | 5.4295 | 2.74E-07 | 0.1936 | 0.0023 |
| rs2407156 | chr5 | 81636368 | RPS23 | -5.4287 | 2.75E-07 | 0.1854 | 0.0023 |
| rs2282475 | chr22 | 24438047 | SUSD2 | 5.4281 | 2.75E-07 | 0.1810 | 0.0023 |
| rs7578692 | chr2 | 69593314 | GFPT1 | -5.4273 | 2.76E-07 | 0.1807 | 0.0023 |
| rs6519120 | chr22 | 38991393 | CBY1 | 5.4255 | 2.79E-07 | 0.1875 | 0.0023 |
| rs4715340 | chr6 | 52688621 | GSTA3 | -5.4252 | 2.79E-07 | 0.1852 | 0.0023 |
| rs8110452 | chr19 | 998126 | WDR18 | 5.4233 | 2.81E-07 | 0.2108 | 0.0023 |
| rs6004668 | chr22 | 25874502 | CRYBB2 | 5.4225 | 2.82E-07 | 0.1939 | 0.0024 |
| rs4075937 | chr7 | 1198743 | ZFAND2A | 5.4194 | 2.86E-07 | 0.1829 | 0.0024 |
| rs1890992 | chr9 | 116319660 | C9orf43 | -5.4181 | 2.88E-07 | 0.1870 | 0.0024 |
| rs2305634 | chr3 | 47043538 | CCDC12 | -5.4101 | 2.99E-07 | 0.1996 | 0.0025 |
| rs3755124 | chr2 | 171784024 | TLK1 | 5.4088 | 3.00E-07 | 0.1797 | 0.0025 |
| rs3748872 | chr2 | 234130 | SH3YL1 | 5.4083 | 3.01E-07 | 0.1893 | 0.0025 |
| rs4473430 | chr2 | 69582895 | GFPT1 | -5.4076 | 3.02E-07 | 0.1843 | 0.0025 |
| rs6725340 | chr2 | 69613926 | GFPT1 | -5.4050 | 3.06E-07 | 0.1836 | 0.0025 |
| rs10972063 | chr9 | 34336342 | NUDT2 | 5.4029 | 3.09E-07 | 0.1797 | 0.0025 |
| rs6478776 | chr9 | 130198866 | RPL12 | 5.4020 | 3.10E-07 | 0.1801 | 0.0025 |
| rs7869023 | chr9 | 130201678 | RPL12 | 5.4020 | 3.10E-07 | 0.1801 | 0.0025 |
| rs2247573 | chr9 | 130210946 | RPL12 | 5.4020 | 3.10E-07 | 0.1801 | 0.0025 |
| rs7859457 | chr9 | 130202596 | RPL12 | 5.4000 | 3.13E-07 | 0.1868 | 0.0026 |
| rs17689863 | chr2 | 75882399 | MRPL19 | -5.3997 | 3.13E-07 | 0.1803 | 0.0026 |
| rs6973278 | chr7 | 866469 | PRKAR1B | -5.3962 | 3.18E-07 | 0.2053 | 0.0026 |
| rs7869823 | chr9 | 130202089 | RPL12 | 5.3962 | 3.18E-07 | 0.1798 | 0.0026 |
| rs6004673 | chr22 | 25885353 | CRYBB2 | 5.3957 | 3.19E-07 | 0.1949 | 0.0026 |
| rs12702080 | chr7 | 1141245 | ZFAND2A | -5.3950 | 3.20E-07 | 0.1951 | 0.0026 |
| rs175435 | chr14 | 75612025 | MLH3 | -5.3927 | 3.23E-07 | 0.2871 | 0.0026 |
| rs158914 | chr5 | 60246409 | ERCC8 | 5.3911 | 3.26E-07 | 0.1919 | 0.0026 |
| rs6050422 | chr20 | 25177920 | ABHD12 | -5.3905 | 3.27E-07 | 0.2106 | 0.0026 |
| rs2319444 | chr14 | 21203950 | RNASE1 | -5.3894 | 3.28E-07 | 0.1953 | 0.0026 |
| rs6509544 | chr19 | 52003331 | SIGLEC12 | 5.3860 | 3.33E-07 | 0.1784 | 0.0027 |
| rs11639420 | chr15 | 45559268 | SLC28A2 | -5.3840 | 3.36E-07 | 0.1987 | 0.0027 |
| rs9645664 | chr11 | 118873987 | TRAPPC4 | 5.3829 | 3.38E-07 | 0.1782 | 0.0027 |
| rs11114978 | chr12 | 77074015 | BBS10 | -5.3821 | 3.39E-07 | 0.1830 | 0.0027 |
| rs2665797 | chr17 | 61922485 | FTSJ3 | -5.3814 | 3.40E-07 | 0.1817 | 0.0027 |
| rs10165899 | chr2 | 75883276 | MRPL19 | -5.3789 | 3.44E-07 | 0.1811 | 0.0027 |
| rs4722034 | chr7 | 912755 | PRKAR1B | -5.3789 | 3.44E-07 | 0.1781 | 0.0027 |
| rs138711 | chr22 | 39139913 | CBY1 | -5.3726 | 3.54E-07 | 0.1865 | 0.0028 |
| rs801167 | chr5 | 140081423 | ZMAT2 | -5.3722 | 3.55E-07 | 0.2139 | 0.0028 |
| rs10519018 | chr15 | 45549265 | SLC28A2 | -5.3664 | 3.64E-07 | 0.1877 | 0.0029 |
| rs2267064 | chr22 | 24544632 | SUSD2 | 5.3644 | 3.67E-07 | 0.1787 | 0.0029 |
| rs595782 | chr11 | 118985151 | DPAGT1 | 5.3622 | 3.71E-07 | 0.2103 | 0.0029 |
| rs11136002 | chr8 | 22217082 | POLR3D | -5.3522 | 3.88E-07 | 0.1873 | 0.0031 |
| rs4147615 | chr6 | 52657321 | GSTA3 | -5.3517 | 3.89E-07 | 0.1853 | 0.0031 |
| rs3810427 | chr19 | 16046650 | CYP4F11 | 5.3507 | 3.91E-07 | 0.2125 | 0.0031 |
| rs426384 | chr20 | 44374806 | WFDC3 | -5.3494 | 3.93E-07 | 0.1897 | 0.0031 |
| rs2268879 | chr20 | 25177805 | ABHD12 | -5.3491 | 3.94E-07 | 0.2081 | 0.0031 |
| rs9876658 | chr3 | 134179453 | ANAPC13 | -5.3491 | 3.94E-07 | 0.1845 | 0.0031 |
| rs4617711 | chr13 | 31809940 | B3GALTL | 5.3485 | 3.95E-07 | 0.1854 | 0.0031 |
| rs3785073 | chr16 | 69401937 | SNTB2 | 5.3473 | 3.97E-07 | 0.1762 | 0.0031 |
| rs1929638 | chr9 | 34272258 | NUDT2 | 5.3467 | 3.98E-07 | 0.1767 | 0.0031 |
| rs7259554 | chr19 | 41125177 | SHKBP1 | 5.3446 | 4.02E-07 | 0.1760 | 0.0031 |
| rs4728142 | chr7 | 128573967 | IRF5 | 5.3434 | 4.04E-07 | 0.2006 | 0.0031 |
| rs7220650 | chr17 | 37487168 | CASC3 | 5.3434 | 4.04E-07 | 0.3460 | 0.0031 |
| rs2072795 | chr22 | 39064209 | CBY1 | 5.3401 | 4.10E-07 | 0.1768 | 0.0032 |
| rs10948725 | chr6 | 52688601 | GSTA3 | -5.3400 | 4.10E-07 | 0.1864 | 0.0032 |
| rs7909832 | chr10 | 16556710 | PTER | 5.3380 | 4.14E-07 | 0.2488 | 0.0032 |
| rs17023171 | chr1 | 119593268 | WARS2 | 5.3363 | 4.17E-07 | 0.1757 | 0.0032 |
| rs4821807 | chr22 | 39065582 | CBY1 | 5.3362 | 4.17E-07 | 0.1792 | 0.0032 |
| rs6606731 | chr12 | 109982578 | UBE3B | -5.3362 | 4.17E-07 | 0.1756 | 0.0032 |
| rs7836759 | chr8 | 28977112 | HMBOX1 | -5.3361 | 4.18E-07 | 0.2043 | 0.0032 |
| rs291449 | chr2 | 191177467 | HIBCH | -5.3357 | 4.18E-07 | 0.1762 | 0.0032 |
| rs2665795 | chr17 | 61925272 | FTSJ3 | -5.3331 | 4.23E-07 | 0.1817 | 0.0032 |
| rs17690224 | chr2 | 75892111 | MRPL19 | -5.3313 | 4.27E-07 | 0.1753 | 0.0033 |
| rs4853169 | chr2 | 75906972 | MRPL19 | -5.3313 | 4.27E-07 | 0.1753 | 0.0033 |
| rs6675409 | chr1 | 90134392 | LRRC8C | 5.3301 | 4.29E-07 | 0.1803 | 0.0033 |
| rs170563 | chr4 | 103606672 | MANBA | 5.3299 | 4.29E-07 | 0.1805 | 0.0033 |
| rs10817476 | chr9 | 116066958 | WDR31 | -5.3297 | 4.30E-07 | 0.1904 | 0.0033 |
| rs9309330 | chr2 | 60977984 | PAPOLG | 5.3293 | 4.30E-07 | 0.1752 | 0.0033 |
| rs2057130 | chr14 | 64961479 | ZBTB1 | 5.3288 | 4.32E-07 | 0.1852 | 0.0033 |
| rs2425125 | chr20 | 34330954 | CPNE1 | -5.3282 | 4.33E-07 | 0.1937 | 0.0033 |
| rs1467616 | chr10 | 102641691 | MRPL43 | 5.3278 | 4.34E-07 | 0.1765 | 0.0033 |
| rs1984728 | chr6 | 52677591 | GSTA3 | -5.3269 | 4.35E-07 | 0.1853 | 0.0033 |
| rs5757231 | chr22 | 39071658 | CBY1 | 5.3208 | 4.47E-07 | 0.1786 | 0.0034 |
| rs13329053 | chr14 | 64975679 | ZBTB1 | 5.3206 | 4.48E-07 | 0.1799 | 0.0034 |
| rs2626 | chr3 | 150303667 | EIF2A | -5.3199 | 4.49E-07 | 0.1823 | 0.0034 |
| rs34616 | chr5 | 60476776 | ERCC8 | 5.3187 | 4.51E-07 | 0.1816 | 0.0034 |
| rs7681614 | chr4 | 140295651 | RAB33B | -5.3155 | 4.58E-07 | 0.1764 | 0.0034 |
| rs449370 | chr20 | 25451180 | ABHD12 | 5.3131 | 4.63E-07 | 0.1856 | 0.0035 |
| rs2390794 | chr1 | 90125566 | LRRC8C | 5.3128 | 4.64E-07 | 0.1791 | 0.0035 |
| rs10210517 | chr2 | 60969547 | PAPOLG | 5.3108 | 4.68E-07 | 0.1762 | 0.0035 |
| rs6004667 | chr22 | 25874464 | CRYBB2 | 5.3083 | 4.73E-07 | 0.1836 | 0.0035 |
| rs2769707 | chr9 | 34167721 | NUDT2 | -5.3082 | 4.74E-07 | 0.1740 | 0.0035 |
| rs7862080 | chr9 | 34303922 | NUDT2 | -5.3082 | 4.74E-07 | 0.1740 | 0.0035 |
| rs6119625 | chr20 | 34160455 | CPNE1 | 5.3073 | 4.75E-07 | 0.1925 | 0.0035 |
| rs7263437 | chr20 | 44410887 | WFDC3 | -5.3051 | 4.80E-07 | 0.1867 | 0.0035 |
| rs5750630 | chr22 | 38985818 | CBY1 | 5.3047 | 4.81E-07 | 0.1826 | 0.0035 |
| rs11917361 | chr3 | 47070497 | CCDC12 | -5.3023 | 4.86E-07 | 0.1739 | 0.0036 |
| rs5750627 | chr22 | 38982321 | CBY1 | -5.3011 | 4.89E-07 | 0.1821 | 0.0036 |
| rs2649663 | chr11 | 57357305 | TIMM10 | 5.2969 | 4.98E-07 | 0.1734 | 0.0037 |
| rs5757133 | chr22 | 38947835 | CBY1 | 5.2965 | 4.99E-07 | 0.1776 | 0.0037 |
| rs9420407 | chr10 | 88721035 | BMPR1A | 5.2945 | 5.03E-07 | 0.1775 | 0.0037 |
| rs34638 | chr5 | 60350487 | ERCC8 | 5.2930 | 5.07E-07 | 0.1892 | 0.0037 |
| rs2960840 | chr7 | 1204700 | ZFAND2A | 5.2919 | 5.09E-07 | 0.1786 | 0.0037 |
| rs12338697 | chr9 | 116329986 | C9orf43 | -5.2909 | 5.12E-07 | 0.1740 | 0.0037 |
| rs4822469 | chr22 | 24425114 | SUSD2 | 5.2896 | 5.15E-07 | 0.1737 | 0.0037 |
| rs203466 | chr17 | 19809953 | AKAP10 | 5.2867 | 5.21E-07 | 0.2001 | 0.0038 |
| rs162984 | chr6 | 155632938 | TFB1M | 5.2862 | 5.23E-07 | 0.1728 | 0.0038 |
| rs4560233 | chr22 | 38982442 | CBY1 | 5.2837 | 5.28E-07 | 0.1803 | 0.0038 |
| rs6785027 | chr3 | 134211649 | ANAPC13 | -5.2824 | 5.32E-07 | 0.1734 | 0.0038 |
| rs9394931 | chr6 | 42873245 | PEX6 | -5.2822 | 5.32E-07 | 0.1857 | 0.0038 |
| rs4815417 | chr20 | 25379526 | ABHD12 | 5.2784 | 5.41E-07 | 0.2148 | 0.0039 |
| rs153047 | chr16 | 69310136 | TMED6 | -5.2770 | 5.45E-07 | 0.1723 | 0.0039 |
| rs9371371 | chr6 | 155625104 | TFB1M | 5.2747 | 5.50E-07 | 0.1858 | 0.0040 |
| rs1386504 | chr20 | 44351515 | WFDC3 | -5.2736 | 5.53E-07 | 0.2493 | 0.0040 |
| rs6956265 | chr7 | 1204504 | ZFAND2A | 5.2696 | 5.63E-07 | 0.1741 | 0.0040 |
| rs6060741 | chr20 | 34571484 | CPNE1 | 5.2686 | 5.65E-07 | 0.1849 | 0.0040 |
| rs8180104 | chr3 | 150244772 | EIF2A | 5.2686 | 5.65E-07 | 0.1834 | 0.0040 |
| rs4732657 | chr8 | 28755988 | HMBOX1 | -5.2680 | 5.67E-07 | 0.1970 | 0.0040 |
| rs12620435 | chr2 | 201995779 | CFLAR | -5.2644 | 5.76E-07 | 0.1716 | 0.0041 |
| rs801176 | chr5 | 140087010 | ZMAT2 | -5.2640 | 5.77E-07 | 0.1814 | 0.0041 |
| rs446649 | chr20 | 25467359 | ABHD12 | 5.2635 | 5.79E-07 | 0.2034 | 0.0041 |
| rs13419008 | chr2 | 61027891 | PAPOLG | 5.2607 | 5.86E-07 | 0.1722 | 0.0042 |
| rs8140617 | chr22 | 38970439 | CBY1 | 5.2600 | 5.87E-07 | 0.1903 | 0.0042 |
| rs7853047 | chr9 | 130198100 | RPL12 | 5.2595 | 5.89E-07 | 0.1713 | 0.0042 |
| rs4976682 | chr5 | 176726002 | PRELID1 | -5.2575 | 5.94E-07 | 0.1879 | 0.0042 |
| rs11811946 | chr1 | 65952428 | LEPROT | 5.2556 | 5.99E-07 | 0.1729 | 0.0042 |
| rs11180460 | chr12 | 75716278 | GLIPR1L2 | -5.2555 | 5.99E-07 | 0.1751 | 0.0042 |
| rs2815171 | chr9 | 85590820 | RASEF | 5.2553 | 6.00E-07 | 0.1727 | 0.0042 |
| rs279571 | chr3 | 9952788 | JAGN1 | -5.2536 | 6.05E-07 | 0.1743 | 0.0043 |
| rs10888650 | chr1 | 39507161 | NDUFS5 | -5.2492 | 6.17E-07 | 0.1997 | 0.0043 |
| rs153045 | chr16 | 69397393 | TMED6 | -5.2447 | 6.29E-07 | 0.1748 | 0.0044 |
| rs34624 | chr5 | 60459716 | ERCC8 | 5.2433 | 6.33E-07 | 0.1707 | 0.0044 |
| rs4700407 | chr5 | 60366528 | ERCC8 | 5.2398 | 6.43E-07 | 0.1927 | 0.0045 |
| rs267303 | chr5 | 114586950 | PGGT1B | 5.2344 | 6.59E-07 | 0.1765 | 0.0046 |
| rs1047799 | chr17 | 17095487 | DRG2 | 5.2329 | 6.63E-07 | 0.2577 | 0.0046 |
| rs3741384 | chr11 | 65211505 | DPF2 | -5.2296 | 6.73E-07 | 0.1702 | 0.0047 |
| rs2238751 | chr22 | 19057790 | DGCR14 | 5.2282 | 6.77E-07 | 0.1744 | 0.0047 |
| rs232262 | chr20 | 44350931 | WFDC3 | -5.2264 | 6.83E-07 | 0.2083 | 0.0047 |
| rs699517 | chr18 | 673016 | ENOSF1 | 5.2242 | 6.89E-07 | 0.2154 | 0.0048 |
| rs7808838 | chr7 | 42472505 | HECW1 | -5.2236 | 6.91E-07 | 0.1772 | 0.0048 |
| rs11084083 | chr19 | 52009560 | SIGLEC12 | 5.2221 | 6.96E-07 | 0.1700 | 0.0048 |
| rs6693717 | chr1 | 90132480 | LRRC8C | 5.2206 | 7.00E-07 | 0.1767 | 0.0048 |
| rs4784669 | chr16 | 56481282 | AMFR | 5.2176 | 7.10E-07 | 0.1701 | 0.0049 |
| rs11868785 | chr17 | 34139218 | RDM1 | -5.2171 | 7.11E-07 | 0.1689 | 0.0049 |
| rs295559 | chr5 | 60401222 | ERCC8 | 5.2156 | 7.16E-07 | 0.1748 | 0.0049 |
| rs11180435 | chr12 | 75664046 | GLIPR1L2 | -5.2153 | 7.17E-07 | 0.1688 | 0.0049 |
| rs2380927 | chr9 | 34282429 | NUDT2 | -5.2136 | 7.22E-07 | 0.1703 | 0.0050 |
| rs6037097 | chr20 | 25347221 | ABHD12 | 5.2091 | 7.37E-07 | 0.1981 | 0.0051 |
| rs11915964 | chr3 | 134348930 | ANAPC13 | 5.2085 | 7.39E-07 | 0.1694 | 0.0051 |
| rs7809922 | chr7 | 55823581 | PSPH | 5.2072 | 7.44E-07 | 0.1688 | 0.0051 |
| rs10516495 | chr4 | 103900090 | MANBA | 5.2059 | 7.48E-07 | 0.1791 | 0.0051 |
| rs1382914 | chr5 | 60266666 | ERCC8 | 5.2053 | 7.50E-07 | 0.1709 | 0.0051 |
| rs7301561 | chr12 | 75669635 | GLIPR1L2 | -5.2037 | 7.55E-07 | 0.1729 | 0.0051 |
| rs6142474 | chr20 | 34574914 | CPNE1 | -5.2026 | 7.59E-07 | 0.1865 | 0.0052 |
| rs10922690 | chr1 | 90132069 | LRRC8C | 5.2023 | 7.60E-07 | 0.1743 | 0.0052 |
| rs175048 | chr14 | 75479894 | MLH3 | -5.1996 | 7.69E-07 | 0.1890 | 0.0052 |
| rs4820335 | chr22 | 38935927 | CBY1 | -5.1965 | 7.80E-07 | 0.1773 | 0.0053 |
| rs9531124 | chr13 | 31927523 | B3GALTL | 5.1928 | 7.93E-07 | 0.2214 | 0.0054 |
| rs4878564 | chr9 | 34375450 | NUDT2 | 5.1906 | 8.00E-07 | 0.1697 | 0.0054 |
| rs2267062 | chr22 | 24544482 | SUSD2 | 5.1882 | 8.09E-07 | 0.1693 | 0.0055 |
| rs7773095 | chr6 | 109727077 | SMPD2 | 5.1869 | 8.14E-07 | 0.1680 | 0.0055 |
| rs12528822 | chr6 | 109661541 | SMPD2 | 5.1868 | 8.14E-07 | 0.1696 | 0.0055 |
| rs7250643 | chr19 | 16815476 | OR10H3 | 5.1847 | 8.21E-07 | 0.1682 | 0.0055 |
| rs2243906 | chr9 | 130219669 | RPL12 | 5.1841 | 8.24E-07 | 0.1759 | 0.0055 |
| rs7141239 | chr14 | 92988495 | LGMN | 5.1835 | 8.26E-07 | 0.1756 | 0.0055 |
| rs11066737 | chr12 | 109904792 | UBE3B | -5.1811 | 8.35E-07 | 0.1669 | 0.0056 |
| rs9436746 | chr1 | 65908473 | LEPROT | 5.1806 | 8.37E-07 | 0.1713 | 0.0056 |
| rs2749010 | chr6 | 52626572 | GSTA3 | -5.1793 | 8.42E-07 | 0.1692 | 0.0056 |
| rs1410116 | chr13 | 48685916 | MED4 | -5.1762 | 8.53E-07 | 0.1683 | 0.0057 |
| rs12600578 | chr17 | 5299950 | RABEP1 | -5.1760 | 8.54E-07 | 0.2062 | 0.0057 |
| rs8180040 | chr3 | 47388947 | CCDC12 | -5.1685 | 8.83E-07 | 0.1664 | 0.0059 |
| rs1567757 | chr12 | 53082253 | KRT1 | -5.1683 | 8.84E-07 | 0.1673 | 0.0059 |
| rs1915943 | chr3 | 150316954 | EIF2A | 5.1671 | 8.88E-07 | 0.1664 | 0.0059 |
| rs1289663 | chr1 | 117643482 | TRIM45 | 5.1640 | 9.00E-07 | 0.1663 | 0.0060 |
| rs7991892 | chr13 | 48516757 | MED4 | -5.1606 | 9.14E-07 | 0.1672 | 0.0060 |
| rs10818618 | chr9 | 124799240 | NDUFA8 | -5.1605 | 9.14E-07 | 0.1658 | 0.0060 |
| rs7531110 | chr1 | 65937065 | LEPROT | 5.1598 | 9.17E-07 | 0.1657 | 0.0060 |
| rs15872 | chr18 | 668258 | ENOSF1 | 5.1590 | 9.20E-07 | 0.1701 | 0.0061 |
| rs1558139 | chr19 | 15997564 | CYP4F11 | 5.1585 | 9.22E-07 | 0.1687 | 0.0061 |
| rs411817 | chr5 | 94977654 | SPATA9 | 5.1579 | 9.25E-07 | 0.2104 | 0.0061 |
| rs10205561 | chr2 | 201809704 | PPIL3 | -5.1564 | 9.31E-07 | 0.1655 | 0.0061 |
| rs7857918 | chr9 | 85717473 | RASEF | -5.1543 | 9.40E-07 | 0.1806 | 0.0062 |
| rs4762652 | chr12 | 96351097 | AMDHD1 | 5.1542 | 9.40E-07 | 0.1705 | 0.0062 |
| rs13088298 | chr3 | 179115501 | MFN1 | 5.1505 | 9.56E-07 | 0.1667 | 0.0062 |
| rs1057077 | chr12 | 7022085 | LRRC23 | 5.1492 | 9.61E-07 | 0.2010 | 0.0063 |
| rs12655209 | chr5 | 60306052 | ERCC8 | 5.1474 | 9.69E-07 | 0.1772 | 0.0063 |
| rs16871660 | chr5 | 73619435 | FOXD1 | 5.1451 | 9.79E-07 | 0.1649 | 0.0064 |
| rs11954279 | chr5 | 73629001 | FOXD1 | 5.1451 | 9.79E-07 | 0.1649 | 0.0064 |
| rs11950285 | chr5 | 73629223 | FOXD1 | 5.1451 | 9.79E-07 | 0.1649 | 0.0064 |
| rs10923759 | chr1 | 119692194 | WARS2 | 5.1442 | 9.83E-07 | 0.1679 | 0.0064 |
| rs12658729 | chr5 | 31553968 | C5orf22 | 5.1399 | 1.00E-06 | 0.1653 | 0.0065 |
| rs2257991 | chr20 | 25270339 | ABHD12 | 5.1397 | 1.00E-06 | 0.1957 | 0.0065 |
| rs2999542 | chr1 | 151899559 | THEM4 | 5.1385 | 1.01E-06 | 0.1719 | 0.0065 |
| rs883025 | chr9 | 116325499 | C9orf43 | -5.1380 | 1.01E-06 | 0.1707 | 0.0065 |
| rs726824 | chr5 | 60332816 | ERCC8 | 5.1377 | 1.01E-06 | 0.1923 | 0.0065 |
| rs6050611 | chr20 | 25414419 | ABHD12 | 5.1374 | 1.01E-06 | 0.1981 | 0.0065 |
| rs11653282 | chr17 | 34236746 | RDM1 | -5.1366 | 1.02E-06 | 0.1652 | 0.0065 |
| rs11153019 | chr6 | 107090614 | QRSL1 | 5.1345 | 1.03E-06 | 0.1650 | 0.0066 |
| rs619250 | chr11 | 118064582 | MPZL3 | 5.1335 | 1.03E-06 | 0.1659 | 0.0066 |
| rs801168 | chr5 | 140080298 | ZMAT2 | -5.1287 | 1.05E-06 | 0.1682 | 0.0067 |
| rs7220650 | chr17 | 37487168 | STARD3 | 5.1286 | 1.05E-06 | 0.3767 | 0.0067 |
| rs138702 | chr22 | 39132601 | CBY1 | -5.1278 | 1.06E-06 | 0.1646 | 0.0067 |
| rs4975274 | chr4 | 129778076 | C4orf33 | 5.1275 | 1.06E-06 | 0.1653 | 0.0067 |
| rs2162126 | chr4 | 129779076 | C4orf33 | 5.1275 | 1.06E-06 | 0.1653 | 0.0067 |
| rs1354536 | chr7 | 42792422 | HECW1 | -5.1264 | 1.06E-06 | 0.1641 | 0.0068 |
| rs3732304 | chr2 | 75882855 | MRPL19 | -5.1261 | 1.06E-06 | 0.1658 | 0.0068 |
| rs295561 | chr5 | 60400217 | ERCC8 | 5.1242 | 1.07E-06 | 0.1661 | 0.0068 |
| rs2442781 | chr3 | 11483769 | ATG7 | 5.1236 | 1.08E-06 | 0.1662 | 0.0068 |
| rs2590970 | chr20 | 34654944 | CPNE1 | 5.1206 | 1.09E-06 | 0.1777 | 0.0069 |
| rs6681392 | chr1 | 154796712 | TPM3 | -5.1192 | 1.10E-06 | 0.1674 | 0.0069 |
| rs13004273 | chr2 | 171871997 | TLK1 | 5.1185 | 1.10E-06 | 0.1773 | 0.0069 |
| rs224823 | chr5 | 81605353 | RPS23 | 5.1172 | 1.11E-06 | 0.1675 | 0.0070 |
| rs3789442 | chr1 | 94581456 | ALG14 | 5.1166 | 1.11E-06 | 0.1698 | 0.0070 |
| rs1026619 | chr6 | 107114361 | QRSL1 | 5.1147 | 1.12E-06 | 0.1745 | 0.0070 |
| rs12459634 | chr19 | 36230174 | U2AF1L4 | 5.1128 | 1.13E-06 | 0.1653 | 0.0071 |
| rs873833 | chr22 | 24427878 | SUSD2 | 5.1106 | 1.14E-06 | 0.1655 | 0.0071 |
| rs12347895 | chr9 | 116345471 | C9orf43 | -5.1104 | 1.14E-06 | 0.1669 | 0.0071 |
| rs4968661 | chr17 | 61796306 | FTSJ3 | -5.1103 | 1.14E-06 | 0.1752 | 0.0071 |
| rs6857659 | chr4 | 129763370 | C4orf33 | 5.1077 | 1.15E-06 | 0.1644 | 0.0072 |
| rs9968172 | chr3 | 134231251 | ANAPC13 | -5.1052 | 1.17E-06 | 0.1635 | 0.0073 |
| rs590580 | chr11 | 118066104 | MPZL3 | 5.1030 | 1.18E-06 | 0.1720 | 0.0073 |
| rs119076 | chr14 | 75572419 | MLH3 | -5.1002 | 1.19E-06 | 0.1707 | 0.0074 |
| rs7048825 | chr9 | 71595965 | PIP5K1B | -5.0996 | 1.20E-06 | 0.1628 | 0.0074 |
| rs1846568 | chr11 | 57357714 | TIMM10 | 5.0990 | 1.20E-06 | 0.1714 | 0.0074 |
| rs227284 | chr4 | 103607635 | MANBA | 5.0984 | 1.20E-06 | 0.1631 | 0.0075 |
| rs175490 | chr14 | 75545685 | MLH3 | -5.0974 | 1.21E-06 | 0.1820 | 0.0075 |
| rs2062278 | chr3 | 47416761 | CCDC12 | -5.0968 | 1.21E-06 | 0.1641 | 0.0075 |
| rs9379855 | chr6 | 26364930 | BTN3A2 | -5.0959 | 1.22E-06 | 0.1626 | 0.0075 |
| rs2025804 | chr1 | 65946121 | LEPROT | 5.0956 | 1.22E-06 | 0.1640 | 0.0075 |
| rs6716127 | chr2 | 171983470 | TLK1 | 5.0936 | 1.23E-06 | 0.1700 | 0.0076 |
| rs1984490 | chr1 | 46858734 | FAAH | -5.0932 | 1.23E-06 | 0.1650 | 0.0076 |
| rs6818935 | chr4 | 84810363 | NKX6-1 | -5.0928 | 1.23E-06 | 0.1621 | 0.0076 |
| rs12548248 | chr8 | 120932321 | COLEC10 | -5.0922 | 1.24E-06 | 0.2462 | 0.0076 |
| rs2235230 | chr22 | 39124467 | CBY1 | 5.0917 | 1.24E-06 | 0.1693 | 0.0076 |
| rs251796 | chr16 | 69395434 | TMED6 | -5.0908 | 1.24E-06 | 0.1619 | 0.0076 |
| rs13415061 | chr2 | 60968665 | PAPOLG | 5.0900 | 1.25E-06 | 0.1618 | 0.0076 |
| rs8107014 | chr19 | 41127465 | SHKBP1 | 5.0899 | 1.25E-06 | 0.1627 | 0.0076 |
| rs4447635 | chr2 | 265970 | SH3YL1 | 5.0898 | 1.25E-06 | 0.1628 | 0.0076 |
| rs1058115 | chr17 | 5323269 | RABEP1 | -5.0897 | 1.25E-06 | 0.2033 | 0.0076 |
| rs1127460 | chr7 | 878767 | PRKAR1B | -5.0886 | 1.25E-06 | 0.1618 | 0.0076 |
| rs12551572 | chr9 | 124864385 | NDUFA8 | 5.0882 | 1.26E-06 | 0.1617 | 0.0076 |
| rs9857878 | chr3 | 52109668 | SEMA3G | 5.0875 | 1.26E-06 | 0.1626 | 0.0077 |
| rs4719500 | chr7 | 871456 | PRKAR1B | -5.0849 | 1.28E-06 | 0.1838 | 0.0077 |
| rs2649662 | chr11 | 57352562 | TIMM10 | 5.0833 | 1.28E-06 | 0.1658 | 0.0078 |
| rs175492 | chr14 | 75545220 | MLH3 | -5.0801 | 1.30E-06 | 0.1681 | 0.0079 |
| rs7867807 | chr9 | 124737687 | NDUFA8 | -5.0784 | 1.31E-06 | 0.1628 | 0.0079 |
| rs4722018 | chr7 | 911507 | PRKAR1B | -5.0753 | 1.33E-06 | 0.1611 | 0.0080 |
| rs2243767 | chr9 | 130221388 | RPL12 | 5.0752 | 1.33E-06 | 0.1714 | 0.0080 |
| rs4235483 | chr5 | 60188222 | ERCC8 | 5.0744 | 1.34E-06 | 0.1827 | 0.0080 |
| rs6504178 | chr17 | 61812733 | FTSJ3 | -5.0739 | 1.34E-06 | 0.1719 | 0.0081 |
| rs11637838 | chr15 | 45550657 | SLC28A2 | -5.0736 | 1.34E-06 | 0.1743 | 0.0081 |
| rs12636851 | chr3 | 47463567 | CCDC12 | -5.0665 | 1.38E-06 | 0.1707 | 0.0083 |
| rs6589372 | chr11 | 113212553 | TTC12 | 5.0640 | 1.40E-06 | 0.1664 | 0.0084 |
| rs2564944 | chr3 | 53148455 | ITIH3 | 5.0630 | 1.40E-06 | 0.2017 | 0.0084 |
| rs6050559 | chr20 | 25329915 | ABHD12 | 5.0622 | 1.41E-06 | 0.1955 | 0.0084 |
| rs10006474 | chr4 | 103928047 | MANBA | 5.0620 | 1.41E-06 | 0.1751 | 0.0084 |
| rs12385696 | chr1 | 46818836 | FAAH | -5.0619 | 1.41E-06 | 0.1635 | 0.0084 |
| rs6060435 | chr20 | 34069370 | CPNE1 | -5.0603 | 1.42E-06 | 0.1837 | 0.0085 |
| rs1609407 | chr7 | 33101108 | FKBP9 | -5.0580 | 1.43E-06 | 0.1725 | 0.0085 |
| rs10919039 | chr1 | 162245622 | NOS1AP | 5.0578 | 1.44E-06 | 0.1609 | 0.0085 |
| rs158572 | chr5 | 60239443 | ERCC8 | 5.0564 | 1.44E-06 | 0.1705 | 0.0086 |
| rs7856501 | chr9 | 131969556 | CRAT | -5.0555 | 1.45E-06 | 0.1636 | 0.0086 |
| rs9554903 | chr13 | 103542220 | BIVM | 5.0546 | 1.46E-06 | 0.1749 | 0.0086 |
| rs12108712 | chr5 | 73606197 | FOXD1 | 5.0535 | 1.46E-06 | 0.1667 | 0.0087 |
| rs11764413 | chr7 | 102102164 | POLR2J | 5.0530 | 1.47E-06 | 0.1636 | 0.0087 |
| rs7778360 | chr7 | 902450 | PRKAR1B | -5.0526 | 1.47E-06 | 0.1695 | 0.0087 |
| rs7326381 | chr13 | 48696214 | MED4 | -5.0511 | 1.48E-06 | 0.1599 | 0.0087 |
| rs1460961 | chr5 | 60411832 | ERCC8 | 5.0508 | 1.48E-06 | 0.1695 | 0.0087 |
| rs10518544 | chr4 | 130029206 | C4orf33 | 5.0490 | 1.49E-06 | 0.1596 | 0.0088 |
| rs7965888 | chr12 | 96317224 | AMDHD1 | -5.0488 | 1.49E-06 | 0.1813 | 0.0088 |
| rs1889470 | chr1 | 110141421 | GNAI3 | -5.0460 | 1.51E-06 | 0.1688 | 0.0089 |
| rs2932574 | chr1 | 151982020 | THEM4 | 5.0450 | 1.52E-06 | 0.1639 | 0.0089 |
| rs11956498 | chr5 | 73588752 | FOXD1 | 5.0438 | 1.53E-06 | 0.1609 | 0.0089 |
| rs2257233 | chr20 | 25265984 | ABHD12 | 5.0436 | 1.53E-06 | 0.1894 | 0.0089 |
| rs5969970 | chr23 | 149908236 | MTMR1 | 5.0387 | 1.56E-06 | 0.1810 | 0.0091 |
| rs1057077 | chr12 | 7022085 | SPSB2 | 5.0346 | 1.59E-06 | 0.1723 | 0.0093 |
| rs7870227 | chr9 | 116313582 | C9orf43 | -5.0344 | 1.59E-06 | 0.1648 | 0.0093 |
| rs11934740 | chr4 | 129882748 | C4orf33 | 5.0342 | 1.59E-06 | 0.1638 | 0.0093 |
| rs1793169 | chr11 | 118070264 | MPZL3 | 5.0324 | 1.60E-06 | 0.1609 | 0.0093 |
| rs6574631 | chr14 | 81675772 | GTF2A1 | 5.0294 | 1.62E-06 | 0.1597 | 0.0094 |
| rs6060688 | chr20 | 34516731 | CPNE1 | -5.0291 | 1.63E-06 | 0.2072 | 0.0094 |
| rs11623413 | chr14 | 75588824 | MLH3 | -5.0274 | 1.64E-06 | 0.1717 | 0.0095 |
| rs2904978 | chr11 | 65000478 | DPF2 | -5.0262 | 1.65E-06 | 0.1693 | 0.0095 |
| rs6493144 | chr15 | 45573489 | SLC28A2 | -5.0243 | 1.66E-06 | 0.1676 | 0.0096 |
| rs4839257 | chr1 | 113219388 | ST7L | 5.0233 | 1.67E-06 | 0.1624 | 0.0096 |
| rs13292569 | chr9 | 124767989 | NDUFA8 | -5.0204 | 1.69E-06 | 0.1601 | 0.0097 |
| rs2915756 | chr10 | 118362940 | PNLIPRP2 | 5.0203 | 1.69E-06 | 0.1639 | 0.0097 |
| rs2073529 | chr6 | 26375159 | BTN3A2 | -5.0179 | 1.71E-06 | 0.1581 | 0.0098 |
| rs6976860 | chr7 | 77452146 | TMEM60 | -5.0176 | 1.71E-06 | 0.1668 | 0.0098 |
| rs968719 | chr17 | 61908271 | FTSJ3 | -5.0144 | 1.73E-06 | 0.1645 | 0.0099 |
| rs3856650 | chr3 | 126247198 | CHST13 | -5.0144 | 1.73E-06 | 0.1579 | 0.0099 |
| rs163275 | chr16 | 20672175 | ACSM1 | 5.0126 | 1.75E-06 | 0.1658 | 0.0100 |
| rs10754288 | chr1 | 90143520 | LRRC8C | 5.0117 | 1.75E-06 | 0.1853 | 0.0100 |
| rs162971 | chr6 | 155619023 | TFB1M | 5.0110 | 1.76E-06 | 0.1584 | 0.0101 |
| rs7045680 | chr9 | 34281390 | NUDT2 | -5.0095 | 1.77E-06 | 0.1605 | 0.0101 |
| rs3093156 | chr19 | 16000609 | CYP4F11 | 5.0092 | 1.77E-06 | 0.1589 | 0.0101 |
| rs6800271 | chr3 | 47470787 | CCDC12 | -5.0091 | 1.78E-06 | 0.1574 | 0.0101 |
| rs5751939 | chr22 | 21139242 | SNAP29 | -5.0076 | 1.79E-06 | 0.1892 | 0.0102 |
| rs10182643 | chr2 | 20884586 | C2orf43 | -5.0053 | 1.80E-06 | 0.1592 | 0.0103 |
| rs9645660 | chr11 | 115081563 | CADM1 | -5.0033 | 1.82E-06 | 0.1612 | 0.0103 |
| rs761312 | chr20 | 34687809 | CPNE1 | -5.0021 | 1.83E-06 | 0.1602 | 0.0104 |
| rs9480957 | chr6 | 109751601 | SMPD2 | 5.0017 | 1.83E-06 | 0.1655 | 0.0104 |
| rs7246072 | chr19 | 40025197 | LGALS4 | -5.0007 | 1.84E-06 | 0.1632 | 0.0104 |
| rs2608631 | chr6 | 52626269 | GSTA3 | -5.0000 | 1.85E-06 | 0.1731 | 0.0104 |
| rs4369907 | chr2 | 69571124 | GFPT1 | -4.9991 | 1.85E-06 | 0.1755 | 0.0105 |
| rs227283 | chr4 | 103607253 | MANBA | 4.9969 | 1.87E-06 | 0.1609 | 0.0106 |
| rs12338788 | chr9 | 116214058 | C9orf43 | -4.9954 | 1.88E-06 | 0.1645 | 0.0106 |
| rs759974 | chr17 | 400959 | VPS53 | 4.9954 | 1.88E-06 | 0.2319 | 0.0106 |
| rs8078068 | chr17 | 58168984 | HEATR6 | 4.9939 | 1.90E-06 | 0.1661 | 0.0107 |
| rs4147615 | chr6 | 52657321 | GSTA5 | -4.9931 | 1.90E-06 | 0.1579 | 0.0107 |
| rs12315364 | chr12 | 7009270 | SPSB2 | 4.9914 | 1.92E-06 | 0.1884 | 0.0107 |
| rs17361887 | chr1 | 46834775 | FAAH | -4.9911 | 1.92E-06 | 0.1564 | 0.0107 |
| rs507217 | chr13 | 28208517 | POLR1D | 4.9899 | 1.93E-06 | 0.2330 | 0.0108 |
| rs6574103 | chr14 | 73350291 | NUMB | -4.9891 | 1.94E-06 | 0.1847 | 0.0108 |
| rs7610636 | chr3 | 47064436 | CCDC12 | -4.9889 | 1.94E-06 | 0.1654 | 0.0108 |
| rs6850107 | chr4 | 44633866 | GUF1 | 4.9888 | 1.94E-06 | 0.1587 | 0.0108 |
| rs617026 | chr23 | 71546737 | PIN4 | -4.9883 | 1.94E-06 | 0.1650 | 0.0108 |
| rs11845639 | chr14 | 75570293 | MLH3 | -4.9882 | 1.94E-06 | 0.1709 | 0.0108 |
| rs6979031 | chr7 | 73198616 | WBSCR27 | -4.9880 | 1.95E-06 | 0.1577 | 0.0108 |
| rs10410468 | chr19 | 52026600 | SIGLEC12 | -4.9878 | 1.95E-06 | 0.1567 | 0.0108 |
| rs4766377 | chr12 | 1038742 | RAD52 | -4.9837 | 1.98E-06 | 0.1560 | 0.0110 |
| rs6425939 | chr1 | 35628789 | C1orf216 | -4.9832 | 1.99E-06 | 0.1640 | 0.0110 |
| rs12379823 | chr9 | 71595828 | PIP5K1B | -4.9810 | 2.01E-06 | 0.1577 | 0.0111 |
| rs2012627 | chr14 | 75443103 | MLH3 | -4.9808 | 2.01E-06 | 0.1721 | 0.0111 |
| rs1361906 | chr1 | 119484644 | WARS2 | 4.9799 | 2.01E-06 | 0.1562 | 0.0111 |
| rs7712332 | chr5 | 79957835 | DHFR | 4.9780 | 2.03E-06 | 0.1680 | 0.0112 |
| rs1411078 | chr9 | 131958533 | CRAT | -4.9762 | 2.05E-06 | 0.1556 | 0.0113 |
| rs16871628 | chr5 | 73589260 | FOXD1 | 4.9757 | 2.05E-06 | 0.1556 | 0.0113 |
| rs9525744 | chr13 | 43684498 | DNAJC15 | -4.9743 | 2.06E-06 | 0.1636 | 0.0113 |
| rs13222939 | chr7 | 42488522 | HECW1 | -4.9737 | 2.07E-06 | 0.1567 | 0.0114 |
| rs4715340 | chr6 | 52688621 | GSTA5 | -4.9735 | 2.07E-06 | 0.1590 | 0.0114 |
| rs12315364 | chr12 | 7009270 | LRRC23 | 4.9717 | 2.09E-06 | 0.1839 | 0.0114 |
| rs9915612 | chr17 | 71965954 | DNAI2 | 4.9713 | 2.09E-06 | 0.1555 | 0.0114 |
| rs4700397 | chr5 | 60135962 | ERCC8 | 4.9707 | 2.10E-06 | 0.1628 | 0.0115 |
| rs4251719 | chr17 | 34142362 | RDM1 | -4.9705 | 2.10E-06 | 0.1587 | 0.0115 |
| rs9809820 | chr3 | 150317636 | EIF2A | 4.9698 | 2.10E-06 | 0.1574 | 0.0115 |
| rs11130137 | chr3 | 47578838 | CCDC12 | -4.9690 | 2.11E-06 | 0.1552 | 0.0115 |
| rs1142627 | chr4 | 140373081 | RAB33B | -4.9679 | 2.12E-06 | 0.1652 | 0.0115 |
| rs2253070 | chr9 | 133621504 | GPR107 | 4.9663 | 2.14E-06 | 0.1843 | 0.0116 |
| rs10948723 | chr6 | 52666405 | GSTA5 | -4.9659 | 2.14E-06 | 0.1565 | 0.0116 |
| rs2236582 | chr6 | 109765818 | SMPD2 | 4.9654 | 2.15E-06 | 0.1600 | 0.0116 |
| rs2285583 | chr17 | 15968143 | ZSWIM7 | -4.9646 | 2.15E-06 | 0.1736 | 0.0117 |
| rs1477117 | chr12 | 109888779 | UBE3B | -4.9640 | 2.16E-06 | 0.1560 | 0.0117 |
| rs1289952 | chr1 | 117913965 | MAN1A2 | 4.9633 | 2.16E-06 | 0.1598 | 0.0117 |
| rs11238381 | chr7 | 55891901 | PSPH | 4.9610 | 2.19E-06 | 0.1551 | 0.0118 |
| rs1866207 | chr2 | 60972786 | PAPOLG | 4.9604 | 2.19E-06 | 0.1553 | 0.0118 |
| rs3733784 | chr5 | 7862926 | MTRR | 4.9582 | 2.21E-06 | 0.1564 | 0.0119 |
| rs6627801 | chr23 | 149907532 | MTMR1 | 4.9566 | 2.23E-06 | 0.1688 | 0.0120 |
| rs1045327 | chr2 | 171849612 | TLK1 | 4.9565 | 2.23E-06 | 0.1544 | 0.0120 |
| rs2545475 | chr5 | 60409077 | ERCC8 | -4.9564 | 2.23E-06 | 0.1705 | 0.0120 |
| rs1698231 | chr16 | 467585 | DECR2 | -4.9545 | 2.25E-06 | 0.1762 | 0.0121 |
| rs2949192 | chr7 | 1203841 | ZFAND2A | 4.9544 | 2.25E-06 | 0.1546 | 0.0121 |
| rs133335 | chr22 | 42416056 | CYP2D6 | -4.9540 | 2.25E-06 | 0.1686 | 0.0121 |
| rs1637776 | chr23 | 2837291 | ARSD | -4.9539 | 2.25E-06 | 0.1584 | 0.0121 |
| rs655623 | chr5 | 176534516 | PRELID1 | -4.9526 | 2.27E-06 | 0.1671 | 0.0121 |
| rs869945 | chr1 | 224321637 | FBXO28 | -4.9518 | 2.27E-06 | 0.1704 | 0.0121 |
| rs7332363 | chr13 | 48666761 | MED4 | -4.9496 | 2.30E-06 | 0.1550 | 0.0122 |
| rs6761759 | chr2 | 171944915 | TLK1 | 4.9492 | 2.30E-06 | 0.1541 | 0.0122 |
| rs1035826 | chr2 | 171966000 | TLK1 | 4.9492 | 2.30E-06 | 0.1541 | 0.0122 |
| rs1012729 | chr12 | 987482 | RAD52 | -4.9485 | 2.31E-06 | 0.1663 | 0.0123 |
| rs17565659 | chr13 | 27411229 | CDK8 | -4.9483 | 2.31E-06 | 0.1539 | 0.0123 |
| rs6659228 | chr1 | 46811027 | FAAH | -4.9481 | 2.31E-06 | 0.1601 | 0.0123 |
| rs17311495 | chr12 | 96309015 | NTN4 | -4.9477 | 2.31E-06 | 0.1542 | 0.0123 |
| rs11465413 | chr19 | 7805951 | CLEC4M | -4.9461 | 2.33E-06 | 0.1668 | 0.0123 |
| rs6953327 | chr7 | 105144059 | RINT1 | -4.9444 | 2.35E-06 | 0.1673 | 0.0124 |
| rs12831412 | chr12 | 76781215 | BBS10 | 4.9426 | 2.37E-06 | 0.1543 | 0.0125 |
| rs12465115 | chr2 | 171926763 | TLK1 | 4.9426 | 2.37E-06 | 0.1537 | 0.0125 |
| rs801399 | chr5 | 140034844 | ZMAT2 | -4.9413 | 2.38E-06 | 0.1590 | 0.0125 |
| rs295442 | chr3 | 47335881 | CCDC12 | -4.9408 | 2.38E-06 | 0.1537 | 0.0126 |
| rs9446964 | chr6 | 74342696 | SLC17A5 | 4.9402 | 2.39E-06 | 0.1537 | 0.0126 |
| rs4234465 | chr3 | 47026885 | CCDC12 | -4.9392 | 2.40E-06 | 0.1792 | 0.0126 |
| rs2584608 | chr17 | 61936710 | FTSJ3 | -4.9383 | 2.41E-06 | 0.1645 | 0.0126 |
| rs11870608 | chr17 | 16025670 | ZSWIM7 | 4.9383 | 2.41E-06 | 0.1671 | 0.0126 |
| rs16871648 | chr5 | 73609473 | FOXD1 | 4.9376 | 2.42E-06 | 0.1535 | 0.0127 |
| rs17555250 | chr8 | 29427765 | RBPMS | -4.9363 | 2.43E-06 | 0.1836 | 0.0127 |
| rs1215508 | chr1 | 90088697 | LRRC8C | 4.9363 | 2.43E-06 | 0.1601 | 0.0127 |
| rs4863646 | chr4 | 140225440 | RAB33B | -4.9360 | 2.43E-06 | 0.1621 | 0.0127 |
| rs2267063 | chr22 | 24544607 | SUSD2 | 4.9357 | 2.44E-06 | 0.1578 | 0.0127 |
| rs17121871 | chr11 | 118073344 | MPZL3 | 4.9323 | 2.47E-06 | 0.1531 | 0.0129 |
| rs1805 | chr11 | 118076069 | MPZL3 | 4.9323 | 2.47E-06 | 0.1531 | 0.0129 |
| rs17121879 | chr11 | 118080133 | MPZL3 | 4.9323 | 2.47E-06 | 0.1531 | 0.0129 |
| rs6768031 | chr3 | 130951147 | NEK11 | 4.9315 | 2.48E-06 | 0.2114 | 0.0129 |
| rs6833421 | chr4 | 44673101 | GUF1 | 4.9294 | 2.50E-06 | 0.1612 | 0.0130 |
| rs6001209 | chr22 | 39121252 | CBY1 | -4.9293 | 2.51E-06 | 0.1681 | 0.0130 |
| rs9885646 | chr6 | 109742460 | SMPD2 | 4.9265 | 2.54E-06 | 0.1684 | 0.0131 |
| rs4693655 | chr4 | 84820059 | NKX6-1 | -4.9264 | 2.54E-06 | 0.1548 | 0.0131 |
| rs11135995 | chr8 | 22200148 | POLR3D | -4.9264 | 2.54E-06 | 0.1899 | 0.0131 |
| rs4801753 | chr19 | 48764695 | PPP1R15A | 4.9253 | 2.55E-06 | 0.1596 | 0.0132 |
| rs11632920 | chr15 | 45551264 | SLC28A2 | -4.9250 | 2.55E-06 | 0.1616 | 0.0132 |
| rs493083 | chr11 | 126065861 | FOXRED1 | -4.9230 | 2.57E-06 | 0.1578 | 0.0133 |
| rs10985529 | chr9 | 124869062 | NDUFA8 | -4.9212 | 2.59E-06 | 0.1610 | 0.0134 |
| rs6684274 | chr1 | 46810842 | FAAH | -4.9194 | 2.61E-06 | 0.1539 | 0.0134 |
| rs4541106 | chr17 | 37072192 | RPL19 | 4.9173 | 2.64E-06 | 0.1546 | 0.0136 |
| rs740646 | chr17 | 58142190 | HEATR6 | 4.9125 | 2.69E-06 | 0.1581 | 0.0138 |
| rs4315641 | chr3 | 122068875 | CCDC58 | 4.9124 | 2.69E-06 | 0.1623 | 0.0138 |
| rs9808365 | chr2 | 232343 | SH3YL1 | 4.9110 | 2.71E-06 | 0.1608 | 0.0139 |
| rs163274 | chr16 | 20672335 | ACSM1 | 4.9085 | 2.74E-06 | 0.1520 | 0.0140 |
| rs10136480 | chr14 | 77465460 | ADCK1 | -4.9074 | 2.75E-06 | 0.1688 | 0.0141 |
| rs1886696 | chr20 | 34055706 | CPNE1 | -4.9063 | 2.77E-06 | 0.1702 | 0.0141 |
| rs6968982 | chr7 | 54862831 | SEC61G | -4.9061 | 2.77E-06 | 0.1522 | 0.0141 |
| rs11172047 | chr12 | 57298080 | STAT6 | -4.9050 | 2.78E-06 | 0.1546 | 0.0142 |
| rs12164334 | chr23 | 100974000 | ARMCX6 | -4.9034 | 2.80E-06 | 0.1516 | 0.0143 |
| rs10127731 | chr1 | 65956938 | LEPROT | 4.9005 | 2.84E-06 | 0.1562 | 0.0144 |
| rs12997252 | chr2 | 171870710 | TLK1 | 4.8987 | 2.86E-06 | 0.1518 | 0.0145 |
| rs2886297 | chr19 | 15983971 | CYP4F11 | 4.8973 | 2.87E-06 | 0.1539 | 0.0146 |
| rs6050477 | chr20 | 25221547 | ABHD12 | 4.8965 | 2.88E-06 | 0.2002 | 0.0146 |
| rs279588 | chr3 | 9944937 | JAGN1 | 4.8961 | 2.89E-06 | 0.1607 | 0.0146 |
| rs877534 | chr16 | 69367996 | SNTB2 | 4.8958 | 2.89E-06 | 0.1552 | 0.0146 |
| rs11208654 | chr1 | 65965566 | LEPROT | 4.8958 | 2.89E-06 | 0.1535 | 0.0146 |
| rs918107 | chr12 | 109904198 | UBE3B | -4.8946 | 2.91E-06 | 0.1536 | 0.0147 |
| rs10507401 | chr13 | 31806884 | B3GALTL | 4.8925 | 2.93E-06 | 0.1577 | 0.0148 |
| rs10998845 | chr10 | 71294224 | TSPAN15 | -4.8919 | 2.94E-06 | 0.1573 | 0.0148 |
| rs6115214 | chr20 | 25557426 | ABHD12 | 4.8904 | 2.96E-06 | 0.1768 | 0.0149 |
| rs567348 | chr20 | 44410933 | WFDC3 | -4.8886 | 2.98E-06 | 0.1692 | 0.0150 |
| rs227285 | chr4 | 103607806 | MANBA | 4.8869 | 3.01E-06 | 0.1535 | 0.0151 |
| rs4361425 | chr4 | 44745936 | GUF1 | 4.8867 | 3.01E-06 | 0.1509 | 0.0151 |
| rs17361805 | chr1 | 46813967 | FAAH | -4.8858 | 3.02E-06 | 0.1599 | 0.0151 |
| rs364734 | chr6 | 74309401 | SLC17A5 | 4.8856 | 3.02E-06 | 0.1512 | 0.0151 |
| rs3850093 | chr15 | 75318482 | PPCDC | 4.8847 | 3.03E-06 | 0.1565 | 0.0151 |
| rs7132019 | chr12 | 6992122 | SPSB2 | 4.8847 | 3.03E-06 | 0.1545 | 0.0151 |
| rs7628747 | chr3 | 47001990 | CCDC12 | -4.8844 | 3.04E-06 | 0.1683 | 0.0151 |
| rs10879949 | chr12 | 76036414 | GLIPR1L2 | -4.8844 | 3.04E-06 | 0.1522 | 0.0151 |
| rs4715353 | chr6 | 52703766 | GSTA3 | -4.8844 | 3.04E-06 | 0.1719 | 0.0151 |
| rs175071 | chr14 | 75498177 | MLH3 | -4.8839 | 3.04E-06 | 0.1601 | 0.0152 |
| rs17488270 | chr4 | 16508853 | FBXL5 | 4.8825 | 3.06E-06 | 0.1528 | 0.0152 |
| rs1474052 | chr2 | 224192 | SH3YL1 | 4.8822 | 3.07E-06 | 0.1616 | 0.0152 |
| rs932580 | chr20 | 18424826 | DTD1 | -4.8794 | 3.10E-06 | 0.1610 | 0.0154 |
| rs4902293 | chr14 | 65031572 | ZBTB1 | 4.8747 | 3.17E-06 | 0.1704 | 0.0157 |
| rs6917325 | chr6 | 52666273 | GSTA3 | -4.8741 | 3.17E-06 | 0.1659 | 0.0157 |
| rs801183 | chr5 | 140091115 | ZMAT2 | -4.8734 | 3.18E-06 | 0.1594 | 0.0158 |
| rs11874836 | chr18 | 47360028 | CXXC1 | -4.8712 | 3.21E-06 | 0.8898 | 0.0159 |
| rs2364295 | chr3 | 60800699 | FHIT | 4.8671 | 3.27E-06 | 0.1497 | 0.0162 |
| rs2287192 | chr12 | 109931594 | UBE3B | -4.8664 | 3.28E-06 | 0.1562 | 0.0162 |
| rs178826 | chr17 | 16083684 | ZSWIM7 | 4.8656 | 3.29E-06 | 0.3366 | 0.0162 |
| rs17342414 | chr23 | 101837733 | ARMCX6 | -4.8651 | 3.30E-06 | 0.1501 | 0.0163 |
| rs12092807 | chr1 | 119750644 | WARS2 | 4.8604 | 3.37E-06 | 0.1615 | 0.0166 |
| rs11724396 | chr4 | 44725334 | GUF1 | 4.8590 | 3.39E-06 | 0.1577 | 0.0166 |
| rs3996993 | chr6 | 52725521 | GSTA3 | -4.8590 | 3.39E-06 | 0.1500 | 0.0166 |
| rs6719224 | chr2 | 190634271 | ORMDL1 | 4.8578 | 3.40E-06 | 0.2569 | 0.0167 |
| rs4943293 | chr13 | 31838516 | B3GALTL | 4.8569 | 3.42E-06 | 0.1626 | 0.0167 |
| rs12514827 | chr5 | 178090100 | CLK4 | 4.8564 | 3.42E-06 | 0.1550 | 0.0168 |
| rs2098297 | chr14 | 75619118 | MLH3 | -4.8550 | 3.44E-06 | 0.1629 | 0.0168 |
| rs10801774 | chr1 | 90238155 | LRRC8C | 4.8546 | 3.45E-06 | 0.1526 | 0.0169 |
| rs1056610 | chr22 | 39080078 | CBY1 | 4.8538 | 3.46E-06 | 0.1547 | 0.0169 |
| rs6438726 | chr3 | 122110921 | CCDC58 | 4.8532 | 3.47E-06 | 0.1497 | 0.0169 |
| rs10404460 | chr19 | 33960396 | PEPD | 4.8526 | 3.48E-06 | 0.1564 | 0.0170 |
| rs3819101 | chr18 | 677240 | ENOSF1 | 4.8498 | 3.52E-06 | 0.1565 | 0.0171 |
| rs12581416 | chr12 | 75705541 | GLIPR1L2 | -4.8498 | 3.52E-06 | 0.1521 | 0.0171 |
| rs2649667 | chr11 | 57270509 | TIMM10 | 4.8490 | 3.53E-06 | 0.1486 | 0.0172 |
| rs2158502 | chr12 | 936468 | RAD52 | -4.8483 | 3.54E-06 | 0.1608 | 0.0172 |
| rs7587244 | chr2 | 171976294 | TLK1 | 4.8467 | 3.57E-06 | 0.1485 | 0.0173 |
| rs10188763 | chr2 | 260677 | SH3YL1 | 4.8467 | 3.57E-06 | 0.1550 | 0.0173 |
| rs7045782 | chr9 | 124818049 | NDUFA8 | -4.8464 | 3.57E-06 | 0.1567 | 0.0173 |
| rs2195448 | chr5 | 81541238 | RPS23 | 4.8440 | 3.61E-06 | 0.1495 | 0.0175 |
| rs6107060 | chr20 | 25592476 | ABHD12 | 4.8434 | 3.62E-06 | 0.1728 | 0.0175 |
| rs11346 | chr13 | 43683020 | DNAJC15 | -4.8410 | 3.66E-06 | 0.1626 | 0.0176 |
| rs6440677 | chr3 | 150188234 | WWTR1 | 4.8406 | 3.66E-06 | 0.1494 | 0.0177 |
| rs4438157 | chr13 | 28302011 | MTIF3 | -4.8397 | 3.68E-06 | 0.4421 | 0.0177 |
| rs13384245 | chr2 | 201760937 | PPIL3 | -4.8388 | 3.69E-06 | 0.1531 | 0.0178 |
| rs6528055 | chr23 | 21857591 | MBTPS2 | 4.8360 | 3.73E-06 | 0.1518 | 0.0180 |
| rs6076347 | chr20 | 25398790 | ABHD12 | 4.8354 | 3.74E-06 | 0.1798 | 0.0180 |
| rs11587159 | chr1 | 65987205 | LEPROT | -4.8341 | 3.76E-06 | 0.1494 | 0.0181 |
| rs6058288 | chr20 | 34228680 | CPNE1 | -4.8329 | 3.78E-06 | 0.1959 | 0.0181 |
| rs9397236 | chr6 | 155585233 | TFB1M | 4.8320 | 3.80E-06 | 0.1515 | 0.0182 |
| rs291472 | chr2 | 191190605 | HIBCH | -4.8309 | 3.82E-06 | 0.1569 | 0.0183 |
| rs12343129 | chr9 | 116345565 | C9orf43 | -4.8296 | 3.84E-06 | 0.1475 | 0.0183 |
| rs9927979 | chr16 | 20628688 | ACSM1 | 4.8295 | 3.84E-06 | 0.1475 | 0.0183 |
| rs11647815 | chr16 | 3155006 | ZNF213 | 4.8272 | 3.88E-06 | 0.1477 | 0.0185 |
| rs11067030 | chr12 | 109955317 | UBE3B | -4.8269 | 3.88E-06 | 0.1550 | 0.0185 |
| rs6060582 | chr20 | 34313297 | CPNE1 | 4.8265 | 3.89E-06 | 0.1577 | 0.0185 |
| rs17600797 | chr4 | 44626806 | GUF1 | 4.8254 | 3.91E-06 | 0.1492 | 0.0186 |
| rs4810476 | chr20 | 44522594 | WFDC3 | -4.8238 | 3.93E-06 | 0.1545 | 0.0187 |
| rs6028335 | chr20 | 37845477 | DHX35 | -4.8237 | 3.93E-06 | 0.1526 | 0.0187 |
| rs10183261 | chr2 | 201797676 | PPIL3 | -4.8236 | 3.94E-06 | 0.1529 | 0.0187 |
| rs6462446 | chr7 | 33053281 | FKBP9 | -4.8231 | 3.94E-06 | 0.1485 | 0.0187 |
| rs12831657 | chr12 | 109979267 | UBE3B | -4.8225 | 3.95E-06 | 0.1471 | 0.0187 |
| rs17343349 | chr7 | 105143730 | RINT1 | -4.8217 | 3.97E-06 | 0.1471 | 0.0188 |
| rs1904435 | chr5 | 180322735 | BTNL8 | -4.8208 | 3.98E-06 | 0.1502 | 0.0188 |
| rs6916579 | chr6 | 109851638 | SMPD2 | 4.8192 | 4.01E-06 | 0.1582 | 0.0189 |
| rs162242 | chr5 | 60299072 | ERCC8 | 4.8179 | 4.03E-06 | 0.1614 | 0.0190 |
| rs12062 | chr1 | 46830447 | FAAH | -4.8156 | 4.07E-06 | 0.1546 | 0.0192 |
| rs6494748 | chr15 | 68789191 | ITGA11 | 4.8141 | 4.10E-06 | 0.1774 | 0.0193 |
| rs924175 | chr4 | 44842825 | GUF1 | 4.8141 | 4.10E-06 | 0.1486 | 0.0193 |
| rs10224389 | chr7 | 37455281 | ELMO1 | 4.8140 | 4.10E-06 | 0.1466 | 0.0193 |
| rs1580610 | chr4 | 44775015 | GUF1 | 4.8133 | 4.11E-06 | 0.1468 | 0.0193 |
| rs4693656 | chr4 | 84820334 | NKX6-1 | -4.8129 | 4.12E-06 | 0.1495 | 0.0193 |
| rs10934779 | chr3 | 126273469 | CHST13 | -4.8123 | 4.13E-06 | 0.1493 | 0.0194 |
| rs11086974 | chr20 | 44311598 | WFDC3 | -4.8120 | 4.13E-06 | 0.1509 | 0.0194 |
| rs10950832 | chr7 | 906628 | PRKAR1B | -4.8112 | 4.15E-06 | 0.1664 | 0.0194 |
| rs6735524 | chr2 | 88436001 | THNSL2 | -4.8108 | 4.16E-06 | 0.1628 | 0.0194 |
| rs7861248 | chr9 | 116228925 | C9orf43 | -4.8105 | 4.16E-06 | 0.1554 | 0.0194 |
| rs4943605 | chr13 | 39400169 | COG6 | 4.8074 | 4.21E-06 | 0.1624 | 0.0197 |
| rs3848662 | chr19 | 36216056 | U2AF1L4 | 4.8061 | 4.24E-06 | 0.1516 | 0.0198 |
| rs462202 | chr20 | 44371446 | WFDC3 | -4.8059 | 4.24E-06 | 0.1931 | 0.0198 |
| rs10873174 | chr14 | 65032561 | ZBTB1 | 4.8054 | 4.25E-06 | 0.1843 | 0.0198 |
| rs4917737 | chr10 | 98168154 | SLIT1 | 4.8033 | 4.29E-06 | 0.1463 | 0.0199 |
| rs12739928 | chr1 | 116206951 | ATP1A1 | -4.8030 | 4.29E-06 | 0.1596 | 0.0199 |
| rs3757108 | chr6 | 74365178 | SLC17A5 | 4.8020 | 4.31E-06 | 0.1460 | 0.0200 |
| rs1570841 | chr20 | 34048161 | CPNE1 | -4.8009 | 4.33E-06 | 0.1527 | 0.0201 |
| rs4407834 | chr8 | 28841821 | HMBOX1 | -4.8006 | 4.34E-06 | 0.1802 | 0.0201 |
| rs10282309 | chr7 | 37454710 | ELMO1 | 4.8004 | 4.34E-06 | 0.1493 | 0.0201 |
| rs6695043 | chr1 | 46844530 | FAAH | -4.8003 | 4.34E-06 | 0.1529 | 0.0201 |
| rs592525 | chr11 | 119383853 | HMBS | -4.8003 | 4.34E-06 | 0.1478 | 0.0201 |
| rs9358932 | chr6 | 26362705 | BTN3A2 | -4.7996 | 4.36E-06 | 0.1462 | 0.0201 |
| rs11862597 | chr16 | 30490854 | ZNF764 | 4.7994 | 4.36E-06 | 0.1474 | 0.0201 |
| rs483517 | chr1 | 90233068 | LRRC8C | 4.7984 | 4.38E-06 | 0.1523 | 0.0202 |
| rs6885410 | chr5 | 176740996 | PRELID1 | -4.7979 | 4.39E-06 | 0.1769 | 0.0202 |
| rs10958480 | chr8 | 57278717 | PENK | 4.7977 | 4.39E-06 | 0.1552 | 0.0202 |
| rs4853334 | chr2 | 70360950 | FAM136A | -4.7975 | 4.40E-06 | 0.1496 | 0.0202 |
| rs4858888 | chr3 | 47405305 | CCDC12 | -4.7959 | 4.43E-06 | 0.1550 | 0.0203 |
| rs10971956 | chr9 | 34151280 | NUDT2 | -4.7953 | 4.44E-06 | 0.1569 | 0.0203 |
| rs6747253 | chr2 | 201748415 | PPIL3 | -4.7952 | 4.44E-06 | 0.1497 | 0.0203 |
| rs7323702 | chr13 | 48647827 | MED4 | -4.7951 | 4.44E-06 | 0.1523 | 0.0203 |
| rs7250833 | chr19 | 33937277 | PEPD | 4.7946 | 4.45E-06 | 0.1463 | 0.0203 |
| rs12321987 | chr12 | 57288449 | STAT6 | -4.7934 | 4.47E-06 | 0.1493 | 0.0204 |
| rs11626895 | chr14 | 73313472 | ZFYVE1 | 4.7925 | 4.49E-06 | 0.1498 | 0.0205 |
| rs10801772 | chr1 | 90194899 | LRRC8C | 4.7924 | 4.49E-06 | 0.1500 | 0.0205 |
| rs11604251 | chr11 | 68820429 | MTL5 | 4.7910 | 4.52E-06 | 0.1655 | 0.0206 |
| rs1044707 | chr11 | 491334 | PTDSS2 | -4.7899 | 4.54E-06 | 0.1453 | 0.0207 |
| rs7856689 | chr9 | 116264875 | C9orf43 | -4.7893 | 4.55E-06 | 0.1453 | 0.0207 |
| rs1215507 | chr1 | 90088895 | LRRC8C | 4.7890 | 4.56E-06 | 0.1503 | 0.0207 |
| rs13112128 | chr4 | 103609793 | MANBA | 4.7887 | 4.56E-06 | 0.1455 | 0.0207 |
| rs10102758 | chr8 | 6614606 | DEFA4 | 4.7878 | 4.58E-06 | 0.1521 | 0.0208 |
| rs16837531 | chr3 | 126227185 | CHST13 | -4.7878 | 4.58E-06 | 0.1536 | 0.0208 |
| rs2645290 | chr1 | 119471908 | WARS2 | 4.7874 | 4.59E-06 | 0.1549 | 0.0208 |
| rs2665850 | chr17 | 61954112 | FTSJ3 | -4.7856 | 4.62E-06 | 0.1568 | 0.0209 |
| rs7628631 | chr3 | 47560863 | CCDC12 | -4.7851 | 4.63E-06 | 0.1480 | 0.0209 |
| rs1544252 | chr18 | 57560606 | SEC11C | -4.7835 | 4.66E-06 | 0.3598 | 0.0211 |
| rs2425707 | chr20 | 44224978 | WFDC3 | -4.7830 | 4.67E-06 | 0.1519 | 0.0211 |
| rs2012461 | chr16 | 20500449 | ACSM1 | 4.7824 | 4.68E-06 | 0.1456 | 0.0211 |
| rs10938367 | chr4 | 44832274 | GUF1 | 4.7818 | 4.70E-06 | 0.1449 | 0.0211 |
| rs7248933 | chr19 | 40066565 | LGALS4 | -4.7812 | 4.71E-06 | 0.1532 | 0.0212 |
| rs3756982 | chr6 | 52670444 | GSTA3 | -4.7796 | 4.74E-06 | 0.1615 | 0.0213 |
| rs10502173 | chr11 | 113203023 | TTC12 | 4.7795 | 4.74E-06 | 0.1452 | 0.0213 |
| rs7972490 | chr12 | 1009056 | RAD52 | -4.7789 | 4.76E-06 | 0.1471 | 0.0213 |
| rs170843 | chr6 | 155613732 | TFB1M | 4.7783 | 4.77E-06 | 0.1455 | 0.0214 |
| rs11127466 | chr2 | 278752 | SH3YL1 | 4.7783 | 4.77E-06 | 0.1569 | 0.0214 |
| rs2226955 | chr12 | 6965194 | LRRC23 | 4.7769 | 4.80E-06 | 0.1741 | 0.0214 |
| rs17084735 | chr13 | 27411121 | CDK8 | -4.7768 | 4.80E-06 | 0.1459 | 0.0214 |
| rs3748916 | chr2 | 113984033 | PAX8 | -4.7764 | 4.80E-06 | 0.1468 | 0.0215 |
| rs4720097 | chr7 | 33075109 | FKBP9 | -4.7747 | 4.84E-06 | 0.1513 | 0.0216 |
| rs6627802 | chr23 | 149907961 | MTMR1 | 4.7719 | 4.90E-06 | 0.1500 | 0.0218 |
| rs11597031 | chr10 | 118414835 | PNLIPRP2 | 4.7709 | 4.92E-06 | 0.1944 | 0.0219 |
| rs2864105 | chr19 | 52030490 | SIGLEC12 | 4.7700 | 4.94E-06 | 0.1524 | 0.0220 |
| rs41534051 | chr1 | 46828734 | FAAH | -4.7694 | 4.95E-06 | 0.1486 | 0.0220 |
| rs1405655 | chr19 | 50882619 | NAPSA | -4.7678 | 4.98E-06 | 0.1473 | 0.0221 |
| rs4761423 | chr12 | 76786349 | BBS10 | 4.7678 | 4.98E-06 | 0.1475 | 0.0221 |
| rs1439365 | chr4 | 44616723 | GUF1 | 4.7669 | 5.00E-06 | 0.1662 | 0.0222 |
| rs10032000 | chr4 | 129863157 | C4orf33 | 4.7661 | 5.02E-06 | 0.1511 | 0.0222 |
| rs10818564 | chr9 | 124284413 | STOM | -4.7661 | 5.02E-06 | 0.1472 | 0.0222 |
| rs16968676 | chr19 | 34374910 | LSM14A | -4.7658 | 5.03E-06 | 0.1440 | 0.0222 |
| rs926803 | chr14 | 64977739 | ZBTB1 | -4.7656 | 5.03E-06 | 0.1697 | 0.0222 |
| rs2645295 | chr1 | 119582448 | WARS2 | 4.7648 | 5.05E-06 | 0.1616 | 0.0223 |
| rs524588 | chr11 | 133877553 | B3GAT1 | 4.7621 | 5.10E-06 | 0.1439 | 0.0225 |
| rs4145140 | chr12 | 75668394 | GLIPR1L2 | -4.7617 | 5.11E-06 | 0.1439 | 0.0225 |
| rs107878 | chr19 | 36250259 | U2AF1L4 | -4.7606 | 5.14E-06 | 0.1467 | 0.0226 |
| rs4438157 | chr13 | 28302011 | LNX2 | -4.7600 | 5.15E-06 | 0.3530 | 0.0227 |
| rs1491322 | chr4 | 44663652 | GUF1 | 4.7599 | 5.15E-06 | 0.1439 | 0.0227 |
| rs159358 | chr5 | 60469912 | ERCC8 | 4.7586 | 5.18E-06 | 0.1579 | 0.0228 |
| rs2235229 | chr22 | 39124912 | CBY1 | -4.7574 | 5.20E-06 | 0.1456 | 0.0228 |
| rs7175720 | chr15 | 75348962 | PPCDC | 4.7569 | 5.22E-06 | 0.1488 | 0.0229 |
| rs11156644 | chr14 | 21201589 | RNASE1 | -4.7565 | 5.23E-06 | 0.1569 | 0.0229 |
| rs778597 | chr5 | 140040665 | ZMAT2 | -4.7551 | 5.26E-06 | 0.1551 | 0.0230 |
| rs11183856 | chr12 | 47724158 | SENP1 | -4.7551 | 5.26E-06 | 0.1435 | 0.0230 |
| rs6561150 | chr13 | 44460470 | CCDC122 | 4.7550 | 5.26E-06 | 0.1513 | 0.0230 |
| rs11249756 | chr5 | 180455372 | BTNL8 | -4.7550 | 5.26E-06 | 0.1471 | 0.0230 |
| rs4854302 | chr2 | 305803 | SH3YL1 | 4.7541 | 5.28E-06 | 0.1503 | 0.0230 |
| rs2633339 | chr19 | 16802009 | OR10H3 | 4.7531 | 5.30E-06 | 0.1434 | 0.0231 |
| rs2175605 | chr12 | 76885875 | BBS10 | -4.7524 | 5.32E-06 | 0.1604 | 0.0232 |
| rs6663547 | chr1 | 110114229 | GNAI3 | -4.7502 | 5.37E-06 | 0.1438 | 0.0233 |
| rs12437697 | chr15 | 66978811 | MEGF11 | 4.7502 | 5.37E-06 | 0.1440 | 0.0233 |
| rs9379851 | chr6 | 26354780 | BTN3A2 | -4.7497 | 5.38E-06 | 0.1432 | 0.0234 |
| rs3752135 | chr19 | 52000624 | SIGLEC12 | 4.7478 | 5.42E-06 | 0.1489 | 0.0235 |
| rs11696064 | chr2 | 171920221 | TLK1 | 4.7471 | 5.43E-06 | 0.1444 | 0.0236 |
| rs1358998 | chr6 | 109701218 | SMPD2 | 4.7461 | 5.46E-06 | 0.1656 | 0.0236 |
| rs643864 | chr5 | 140576241 | PCDHB8 | -4.7458 | 5.46E-06 | 0.1500 | 0.0237 |
| rs2881892 | chr4 | 44662307 | GUF1 | 4.7453 | 5.48E-06 | 0.1447 | 0.0237 |
| rs7311488 | chr12 | 109930905 | UBE3B | -4.7450 | 5.48E-06 | 0.1516 | 0.0237 |
| rs7682495 | chr4 | 44665223 | GUF1 | 4.7423 | 5.55E-06 | 0.1491 | 0.0239 |
| rs1002777 | chr11 | 125444604 | PUS3 | 4.7417 | 5.56E-06 | 0.1429 | 0.0240 |
| rs1741895 | chr6 | 74316107 | SLC17A5 | 4.7408 | 5.58E-06 | 0.1452 | 0.0241 |
| rs7955412 | chr12 | 6998371 | SPSB2 | 4.7396 | 5.61E-06 | 0.1463 | 0.0242 |
| rs6546572 | chr2 | 70373642 | FAM136A | -4.7391 | 5.62E-06 | 0.1475 | 0.0242 |
| rs4541106 | chr17 | 37072192 | NEUROD2 | 4.7389 | 5.63E-06 | 0.1432 | 0.0242 |
| rs6823503 | chr4 | 84801998 | NKX6-1 | -4.7386 | 5.63E-06 | 0.1427 | 0.0242 |
| rs740452 | chr17 | 66312238 | ABCA9 | -4.7385 | 5.64E-06 | 0.1443 | 0.0242 |
| rs12091291 | chr1 | 119478828 | WARS2 | 4.7363 | 5.69E-06 | 0.1464 | 0.0244 |
| rs11631021 | chr15 | 45556829 | SLC28A2 | -4.7362 | 5.69E-06 | 0.1527 | 0.0244 |
| rs1646268 | chr16 | 58777196 | KATNB1 | -4.7356 | 5.70E-06 | 0.1473 | 0.0244 |
| rs36450 | chr14 | 72339221 | RGS6 | 4.7339 | 5.74E-06 | 0.1500 | 0.0246 |
| rs1044931 | chr12 | 57449810 | STAT6 | -4.7324 | 5.78E-06 | 0.1485 | 0.0247 |
| rs1984728 | chr6 | 52677591 | GSTA5 | -4.7314 | 5.81E-06 | 0.1489 | 0.0248 |
| rs8058523 | chr16 | 69343361 | SNTB2 | 4.7297 | 5.85E-06 | 0.1642 | 0.0249 |
| rs4803260 | chr19 | 40063077 | LGALS4 | -4.7284 | 5.88E-06 | 0.1469 | 0.0251 |
| rs10254763 | chr7 | 37463968 | ELMO1 | 4.7280 | 5.89E-06 | 0.1427 | 0.0251 |
| rs2854214 | chr17 | 61945449 | FTSJ3 | -4.7254 | 5.95E-06 | 0.1535 | 0.0253 |
| rs462202 | chr20 | 44371446 | WFDC10B | 4.7254 | 5.95E-06 | 0.1752 | 0.0253 |
| rs2298583 | chr18 | 677302 | ENOSF1 | 4.7253 | 5.96E-06 | 0.1527 | 0.0253 |
| rs3827356 | chr22 | 39064806 | CBY1 | 4.7244 | 5.98E-06 | 0.1469 | 0.0254 |
| rs7855325 | chr9 | 116288473 | C9orf43 | 4.7235 | 6.00E-06 | 0.1480 | 0.0254 |
| rs2749026 | chr6 | 52630635 | GSTA3 | -4.7208 | 6.07E-06 | 0.1497 | 0.0257 |
| rs4272003 | chr4 | 84822518 | NKX6-1 | -4.7203 | 6.08E-06 | 0.1470 | 0.0258 |
| rs9912506 | chr17 | 5199038 | RABEP1 | -4.7195 | 6.10E-06 | 0.1743 | 0.0258 |
| rs4968070 | chr17 | 446162 | VPS53 | 4.7164 | 6.18E-06 | 0.1970 | 0.0261 |
| rs2136569 | chr2 | 191167414 | HIBCH | -4.7156 | 6.20E-06 | 0.1518 | 0.0262 |
| rs2749029 | chr6 | 52631188 | GSTA5 | -4.7148 | 6.22E-06 | 0.1422 | 0.0263 |
| rs9814567 | chr3 | 134218557 | ANAPC13 | -4.7139 | 6.25E-06 | 0.1479 | 0.0263 |
| rs1352792 | chr4 | 44665342 | GUF1 | 4.7127 | 6.28E-06 | 0.1424 | 0.0265 |
| rs12105811 | chr2 | 202016245 | PPIL3 | -4.7120 | 6.30E-06 | 0.1506 | 0.0265 |
| rs10876951 | chr12 | 57306430 | STAT6 | -4.7112 | 6.32E-06 | 0.1523 | 0.0266 |
| rs9990351 | chr3 | 134239191 | ANAPC13 | -4.7106 | 6.33E-06 | 0.1451 | 0.0266 |
| rs5996945 | chr22 | 25875573 | CRYBB2 | 4.7104 | 6.34E-06 | 0.1709 | 0.0266 |
| rs11625861 | chr14 | 81735001 | GTF2A1 | 4.7101 | 6.35E-06 | 0.1490 | 0.0266 |
| rs2002341 | chr16 | 3164699 | ZNF213 | 4.7097 | 6.36E-06 | 0.1410 | 0.0266 |
| rs10981835 | chr9 | 116349298 | C9orf43 | -4.7095 | 6.36E-06 | 0.1433 | 0.0266 |
| rs1547525 | chr4 | 44710346 | GUF1 | 4.7094 | 6.37E-06 | 0.1453 | 0.0266 |
| rs2276182 | chr18 | 51798047 | POLI | -4.7078 | 6.41E-06 | 0.1666 | 0.0268 |
| rs9529119 | chr13 | 31783977 | B3GALTL | 4.7076 | 6.41E-06 | 0.1452 | 0.0268 |
| rs3822360 | chr5 | 31542666 | C5orf22 | -4.7060 | 6.46E-06 | 0.1515 | 0.0270 |
| rs646358 | chr11 | 126204161 | FOXRED1 | 4.7039 | 6.51E-06 | 0.1419 | 0.0272 |
| rs7198570 | chr16 | 20618468 | ACSM1 | 4.7034 | 6.53E-06 | 0.1407 | 0.0272 |
| rs163235 | chr16 | 20651378 | ACSM1 | 4.7034 | 6.53E-06 | 0.1407 | 0.0272 |
| rs9395432 | chr6 | 48930865 | CRISP1 | 4.7021 | 6.56E-06 | 0.1406 | 0.0273 |
| rs7580629 | chr2 | 171925228 | TLK1 | 4.6988 | 6.65E-06 | 0.1421 | 0.0276 |
| rs7096909 | chr10 | 82284512 | TSPAN14 | 4.6988 | 6.65E-06 | 0.1405 | 0.0276 |
| rs12797951 | chr11 | 71143266 | NADSYN1 | 4.6987 | 6.66E-06 | 0.1404 | 0.0276 |
| rs462145 | chr20 | 44378211 | WFDC3 | -4.6974 | 6.69E-06 | 0.1482 | 0.0277 |
| rs2569163 | chr5 | 139975466 | ZMAT2 | -4.6973 | 6.70E-06 | 0.1498 | 0.0277 |
| rs4715353 | chr6 | 52703766 | GSTA5 | -4.6972 | 6.70E-06 | 0.1518 | 0.0277 |
| rs7404783 | chr16 | 20618004 | ACSM1 | 4.6970 | 6.70E-06 | 0.1406 | 0.0277 |
| rs8017745 | chr14 | 66720496 | FUT8 | 4.6965 | 6.72E-06 | 0.1403 | 0.0278 |
| rs17259565 | chr12 | 77033239 | BBS10 | 4.6962 | 6.73E-06 | 0.1435 | 0.0278 |
| rs1991370 | chr2 | 171916120 | TLK1 | 4.6941 | 6.79E-06 | 0.1426 | 0.0280 |
| rs10252 | chr1 | 46830430 | NSUN4 | -4.6938 | 6.79E-06 | 0.1435 | 0.0280 |
| rs11615637 | chr12 | 110023689 | UBE3B | -4.6934 | 6.81E-06 | 0.1402 | 0.0280 |
| rs530587 | chr11 | 126064439 | FOXRED1 | -4.6932 | 6.81E-06 | 0.1428 | 0.0280 |
| rs956275 | chr4 | 57267406 | PPAT | 4.6930 | 6.82E-06 | 0.1519 | 0.0280 |
| rs4853319 | chr2 | 70344090 | FAM136A | -4.6918 | 6.85E-06 | 0.1521 | 0.0282 |
| rs2085852 | chr2 | 240630 | SH3YL1 | 4.6910 | 6.87E-06 | 0.1409 | 0.0282 |
| rs1564131 | chr11 | 6254397 | APBB1 | 4.6904 | 6.89E-06 | 0.1402 | 0.0283 |
| rs6035046 | chr20 | 18437799 | DTD1 | -4.6874 | 6.98E-06 | 0.1748 | 0.0286 |
| rs11655099 | chr17 | 2056647 | PITPNA | -4.6862 | 7.01E-06 | 0.1494 | 0.0287 |
| rs11020506 | chr11 | 93478342 | C11orf54 | -4.6862 | 7.01E-06 | 0.1397 | 0.0287 |
| rs3808838 | chr9 | 130157934 | DNM1 | 4.6853 | 7.04E-06 | 0.1426 | 0.0288 |
| rs3087711 | chr12 | 76738542 | BBS10 | 4.6846 | 7.06E-06 | 0.1452 | 0.0288 |
| rs11906429 | chr20 | 44432193 | WFDC3 | -4.6840 | 7.08E-06 | 0.1529 | 0.0289 |
| rs9315986 | chr13 | 43656443 | DNAJC15 | -4.6831 | 7.10E-06 | 0.1487 | 0.0290 |
| rs10893398 | chr11 | 125416163 | PUS3 | 4.6823 | 7.13E-06 | 0.1396 | 0.0291 |
| rs7900536 | chr10 | 82253701 | TSPAN14 | 4.6821 | 7.13E-06 | 0.1397 | 0.0291 |
| rs7553422 | chr1 | 119540719 | WARS2 | 4.6816 | 7.15E-06 | 0.1413 | 0.0291 |
| rs17013645 | chr1 | 119484849 | WARS2 | 4.6813 | 7.16E-06 | 0.1447 | 0.0291 |
| rs595760 | chr11 | 126179662 | FOXRED1 | -4.6808 | 7.17E-06 | 0.1588 | 0.0291 |
| rs10513389 | chr9 | 124821226 | NDUFA8 | -4.6769 | 7.29E-06 | 0.1482 | 0.0296 |
| rs2886168 | chr14 | 78316132 | ADCK1 | 4.6751 | 7.34E-06 | 0.1511 | 0.0298 |
| rs290506 | chr5 | 60298360 | ERCC8 | 4.6747 | 7.36E-06 | 0.1534 | 0.0298 |
| rs17286731 | chr5 | 140083744 | ZMAT2 | 4.6721 | 7.44E-06 | 0.1403 | 0.0301 |
| rs7038589 | chr9 | 116081449 | WDR31 | -4.6718 | 7.45E-06 | 0.1396 | 0.0301 |
| rs223397 | chr4 | 103742651 | MANBA | 4.6710 | 7.47E-06 | 0.1463 | 0.0302 |
| rs12385693 | chr1 | 46818921 | FAAH | -4.6700 | 7.50E-06 | 0.1482 | 0.0303 |
| rs4662730 | chr2 | 128191096 | PROC | -4.6694 | 7.52E-06 | 0.1389 | 0.0304 |
| rs10802075 | chr1 | 119627227 | WARS2 | 4.6676 | 7.58E-06 | 0.1699 | 0.0306 |
| rs9367341 | chr6 | 48984757 | CRISP1 | 4.6667 | 7.60E-06 | 0.1393 | 0.0306 |
| rs476838 | chr6 | 74317842 | SLC17A5 | 4.6658 | 7.63E-06 | 0.1405 | 0.0307 |
| rs8003506 | chr14 | 75586288 | MLH3 | -4.6642 | 7.68E-06 | 0.1463 | 0.0309 |
| rs11844669 | chr14 | 65111195 | ZBTB1 | -4.6639 | 7.70E-06 | 0.1498 | 0.0309 |
| rs2043608 | chr12 | 96354455 | AMDHD1 | 4.6632 | 7.72E-06 | 0.1389 | 0.0310 |
| rs7619451 | chr3 | 134233238 | ANAPC13 | -4.6620 | 7.76E-06 | 0.1401 | 0.0311 |
| rs2009152 | chr4 | 88826972 | SPP1 | 4.6610 | 7.79E-06 | 0.1418 | 0.0312 |
| rs2709296 | chr7 | 54831941 | SEC61G | -4.6587 | 7.86E-06 | 0.1383 | 0.0315 |
| rs719548 | chr2 | 172015052 | TLK1 | 4.6584 | 7.87E-06 | 0.1389 | 0.0315 |
| rs10026088 | chr4 | 44744987 | GUF1 | 4.6576 | 7.90E-06 | 0.1450 | 0.0316 |
| rs11845639 | chr14 | 75570293 | EIF2B2 | -4.6566 | 7.93E-06 | 0.1518 | 0.0317 |
| rs133340 | chr22 | 42417687 | CYP2D6 | -4.6538 | 8.03E-06 | 0.1404 | 0.0321 |
| rs9535573 | chr13 | 51655576 | GUCY1B2 | -4.6534 | 8.04E-06 | 0.1490 | 0.0321 |
| rs3735369 | chr7 | 105138947 | RINT1 | -4.6530 | 8.05E-06 | 0.1394 | 0.0321 |
| rs2251155 | chr17 | 5199100 | RABEP1 | 4.6526 | 8.06E-06 | 0.2224 | 0.0321 |
| rs1481279 | chr4 | 103977938 | MANBA | 4.6525 | 8.07E-06 | 0.1388 | 0.0321 |
| rs6968988 | chr7 | 1169619 | ZFAND2A | -4.6505 | 8.13E-06 | 0.1421 | 0.0323 |
| rs6587632 | chr1 | 151911254 | THEM4 | -4.6505 | 8.14E-06 | 0.1507 | 0.0323 |
| rs9831225 | chr3 | 196274965 | TFRC | 4.6505 | 8.14E-06 | 0.1447 | 0.0323 |
| rs11263830 | chr1 | 36345798 | C1orf216 | -4.6493 | 8.18E-06 | 0.1418 | 0.0325 |
| rs4384823 | chr2 | 69437475 | GFPT1 | -4.6489 | 8.19E-06 | 0.1418 | 0.0325 |
| rs742034 | chr20 | 44522005 | WFDC3 | -4.6481 | 8.22E-06 | 0.1466 | 0.0326 |
| rs12791333 | chr11 | 96077772 | CCDC82 | 4.6478 | 8.23E-06 | 0.1519 | 0.0326 |
| rs7801986 | chr7 | 33083972 | FKBP9 | -4.6477 | 8.23E-06 | 0.1418 | 0.0326 |
| rs4520874 | chr17 | 41415522 | NBR2 | -4.6470 | 8.25E-06 | 0.1520 | 0.0326 |
| rs2281494 | chr20 | 3235494 | ITPA | -4.6465 | 8.27E-06 | 0.1429 | 0.0327 |
| rs4658318 | chr1 | 90232180 | LRRC8C | 4.6464 | 8.27E-06 | 0.1413 | 0.0327 |
| rs2915753 | chr10 | 118364842 | PNLIPRP2 | 4.6459 | 8.29E-06 | 0.1376 | 0.0327 |
| rs9405188 | chr6 | 2997544 | NQO2 | -4.6459 | 8.29E-06 | 0.1447 | 0.0327 |
| rs17377462 | chr8 | 27281912 | PNMA2 | 4.6453 | 8.31E-06 | 0.1682 | 0.0327 |
| rs1113098 | chr4 | 44652187 | GUF1 | 4.6451 | 8.32E-06 | 0.1422 | 0.0327 |
| rs163234 | chr16 | 20652023 | ACSM1 | 4.6441 | 8.35E-06 | 0.1379 | 0.0328 |
| rs12097652 | chr1 | 65085942 | PGM1 | -4.6430 | 8.39E-06 | 0.1378 | 0.0330 |
| rs163238 | chr16 | 20647887 | ACSM1 | 4.6428 | 8.40E-06 | 0.1397 | 0.0330 |
| rs9313209 | chr5 | 7963460 | MTRR | 4.6416 | 8.44E-06 | 0.1397 | 0.0331 |
| rs3818554 | chr6 | 42934620 | GNMT | 4.6409 | 8.46E-06 | 0.1412 | 0.0332 |
| rs3021146 | chr4 | 2958186 | GRK4 | -4.6402 | 8.49E-06 | 0.1738 | 0.0332 |
| rs10056179 | chr5 | 110442137 | STARD4 | -4.6401 | 8.49E-06 | 0.1430 | 0.0332 |
| rs7314535 | chr12 | 7009208 | SPSB2 | 4.6399 | 8.50E-06 | 0.1498 | 0.0332 |
| rs5913035 | chr23 | 78960170 | TBX22 | 4.6395 | 8.51E-06 | 0.1421 | 0.0333 |
| rs2236160 | chr20 | 34101821 | CPNE1 | -4.6367 | 8.61E-06 | 0.1499 | 0.0336 |
| rs7205409 | chr16 | 642610 | WFIKKN1 | -4.6358 | 8.65E-06 | 0.1540 | 0.0337 |
| rs876402 | chr14 | 75601862 | MLH3 | -4.6354 | 8.66E-06 | 0.1509 | 0.0338 |
| rs4305794 | chr7 | 1172588 | ZFAND2A | -4.6346 | 8.69E-06 | 0.1442 | 0.0339 |
| rs9939607 | chr16 | 9216739 | C16orf72 | 4.6341 | 8.70E-06 | 0.1380 | 0.0339 |
| rs3807304 | chr7 | 128644533 | IRF5 | -4.6328 | 8.75E-06 | 0.1403 | 0.0340 |
| rs2241621 | chr14 | 81737076 | GTF2A1 | 4.6325 | 8.76E-06 | 0.1419 | 0.0340 |
| rs17241140 | chr16 | 58532703 | SETD6 | 4.6324 | 8.77E-06 | 0.1388 | 0.0340 |
| rs7597199 | chr2 | 70341575 | FAM136A | -4.6323 | 8.77E-06 | 0.1422 | 0.0340 |
| rs9788945 | chr16 | 58531841 | SETD6 | 4.6320 | 8.78E-06 | 0.1369 | 0.0341 |
| rs223454 | chr4 | 103710930 | MANBA | 4.6316 | 8.80E-06 | 0.1394 | 0.0341 |
| rs12124528 | chr1 | 165126541 | RXRG | 4.6301 | 8.85E-06 | 0.1432 | 0.0343 |
| rs3211995 | chr16 | 2089006 | SLC9A3R2 | -4.6300 | 8.85E-06 | 0.1392 | 0.0343 |
| rs2267546 | chr12 | 4745313 | AKAP3 | 4.6292 | 8.88E-06 | 0.1534 | 0.0343 |
| rs246718 | chr5 | 140522425 | PCDHB8 | -4.6288 | 8.90E-06 | 0.1433 | 0.0344 |
| rs3865350 | chr17 | 5381867 | RABEP1 | 4.6278 | 8.94E-06 | 0.1895 | 0.0345 |
| rs6519301 | chr22 | 42385967 | CYP2D6 | -4.6276 | 8.94E-06 | 0.1374 | 0.0345 |
| rs1779438 | chr1 | 119468316 | WARS2 | 4.6268 | 8.97E-06 | 0.1441 | 0.0346 |
| rs7313450 | chr12 | 104778154 | GLT8D2 | 4.6265 | 8.98E-06 | 0.2066 | 0.0346 |
| rs10774466 | chr12 | 939302 | RAD52 | -4.6254 | 9.02E-06 | 0.1415 | 0.0347 |
| rs9393708 | chr6 | 26362643 | BTN3A2 | -4.6249 | 9.04E-06 | 0.1379 | 0.0348 |
| rs11766651 | chr7 | 1161646 | ZFAND2A | 4.6240 | 9.08E-06 | 0.1444 | 0.0349 |
| rs552085 | chr11 | 125747617 | PUS3 | 4.6237 | 9.09E-06 | 0.1364 | 0.0349 |
| rs13417864 | chr2 | 172042739 | TLK1 | 4.6223 | 9.14E-06 | 0.1451 | 0.0351 |
| rs4319185 | chr9 | 34210872 | NUDT2 | -4.6215 | 9.17E-06 | 0.1395 | 0.0352 |
| rs1650736 | chr5 | 80001990 | DHFR | -4.6205 | 9.21E-06 | 0.1399 | 0.0353 |
| rs11205902 | chr1 | 52348769 | RAB3B | 4.6202 | 9.22E-06 | 0.1397 | 0.0353 |
| rs3809869 | chr17 | 45904163 | MRPL10 | -4.6201 | 9.23E-06 | 0.1427 | 0.0353 |
| rs6853582 | chr4 | 6409954 | CRMP1 | 4.6192 | 9.26E-06 | 0.1362 | 0.0354 |
| rs7487781 | chr12 | 96355376 | AMDHD1 | 4.6192 | 9.26E-06 | 0.1417 | 0.0354 |
| rs10799505 | chr1 | 224351023 | FBXO28 | -4.6180 | 9.30E-06 | 0.1608 | 0.0355 |
| rs10206508 | chr2 | 70829413 | PCYOX1 | 4.6178 | 9.31E-06 | 0.1433 | 0.0355 |
| rs10903667 | chr10 | 2126016 | WDR37 | 4.6175 | 9.32E-06 | 0.1361 | 0.0355 |
| rs4145971 | chr4 | 44843214 | GUF1 | 4.6174 | 9.33E-06 | 0.1380 | 0.0355 |
| rs3789439 | chr1 | 94579426 | ALG14 | 4.6170 | 9.34E-06 | 0.1490 | 0.0355 |
| rs7145159 | chr14 | 75583268 | MLH3 | -4.6163 | 9.37E-06 | 0.1452 | 0.0356 |
| rs3987966 | chr7 | 102358320 | POLR2J | -4.6152 | 9.41E-06 | 0.1377 | 0.0358 |
| rs7843057 | chr8 | 28776909 | HMBOX1 | -4.6146 | 9.44E-06 | 0.1427 | 0.0358 |
| rs13246825 | chr7 | 116789048 | CAV2 | 4.6142 | 9.45E-06 | 0.1359 | 0.0358 |
| rs2818423 | chr10 | 134002148 | DPYSL4 | 4.6138 | 9.47E-06 | 0.1362 | 0.0359 |
| rs1602914 | chr17 | 5190108 | RABEP1 | -4.6127 | 9.51E-06 | 0.2471 | 0.0360 |
| rs12884911 | chr14 | 65027871 | ZBTB1 | 4.6124 | 9.52E-06 | 0.1575 | 0.0360 |
| rs873925 | chr20 | 34687450 | CPNE1 | 4.6122 | 9.53E-06 | 0.1430 | 0.0360 |
| rs11950880 | chr5 | 81189879 | RPS23 | -4.6110 | 9.58E-06 | 0.1377 | 0.0362 |
| rs3924308 | chr9 | 130185385 | RPL12 | -4.6109 | 9.58E-06 | 0.1428 | 0.0362 |
| rs129857 | chr22 | 42399686 | CYP2D6 | -4.6094 | 9.64E-06 | 0.1382 | 0.0364 |
| rs8112732 | chr19 | 16017617 | CYP4F11 | 4.6094 | 9.64E-06 | 0.1397 | 0.0364 |
| rs6769837 | chr3 | 122197984 | CCDC58 | 4.6090 | 9.66E-06 | 0.1356 | 0.0364 |
| rs4923955 | chr15 | 43295088 | PLA2G4E | -4.6064 | 9.76E-06 | 0.1378 | 0.0367 |
| rs12151780 | chr2 | 128199867 | PROC | -4.6046 | 9.83E-06 | 0.1365 | 0.0370 |
| rs1077454 | chr10 | 71517180 | ADAMTS14 | 4.6037 | 9.87E-06 | 0.1383 | 0.0371 |
| rs1284352 | chr11 | 125443473 | PUS3 | 4.6018 | 9.95E-06 | 0.1549 | 0.0374 |
| rs1294201 | chr1 | 233524701 | KIAA1804 | -4.6013 | 9.97E-06 | 0.1376 | 0.0374 |
| rs10948725 | chr6 | 52688601 | GSTA5 | -4.5999 | 1.00E-05 | 0.1481 | 0.0376 |
| rs1777670 | chr13 | 36154966 | SOHLH2 | 4.5998 | 1.00E-05 | 0.1355 | 0.0376 |
| rs267769 | chr5 | 36064642 | SKP2 | -4.5985 | 1.01E-05 | 0.1400 | 0.0377 |
| rs1859710 | chr2 | 75862932 | MRPL19 | -4.5984 | 1.01E-05 | 0.1351 | 0.0377 |
| rs11209478 | chr1 | 69965181 | HHLA3 | -4.5984 | 1.01E-05 | 0.1362 | 0.0377 |
| rs3816779 | chr3 | 47543389 | CCDC12 | -4.5984 | 1.01E-05 | 0.1375 | 0.0377 |
| rs1115053 | chr4 | 44756502 | GUF1 | 4.5980 | 1.01E-05 | 0.1423 | 0.0377 |
| rs1433106 | chr19 | 40073865 | LGALS4 | -4.5966 | 1.02E-05 | 0.1476 | 0.0379 |
| rs1480000 | chr8 | 145702007 | LRRC14 | -4.5962 | 1.02E-05 | 0.1360 | 0.0380 |
| rs5765273 | chr22 | 45747952 | RIBC2 | 4.5959 | 1.02E-05 | 0.1450 | 0.0380 |
| rs4613913 | chr7 | 105153242 | RINT1 | -4.5950 | 1.02E-05 | 0.1394 | 0.0381 |
| rs6503727 | chr17 | 41312859 | NBR2 | -4.5948 | 1.02E-05 | 0.1910 | 0.0381 |
| rs17004059 | chr23 | 112065240 | TRPC5 | 4.5938 | 1.03E-05 | 0.1348 | 0.0382 |
| rs7545737 | chr1 | 119533823 | WARS2 | 4.5927 | 1.03E-05 | 0.1359 | 0.0384 |
| rs581459 | chr1 | 36375110 | C1orf216 | -4.5913 | 1.04E-05 | 0.1399 | 0.0386 |
| rs10890238 | chr1 | 38445654 | SF3A3 | -4.5905 | 1.04E-05 | 0.1347 | 0.0387 |
| rs6460052 | chr7 | 73151644 | WBSCR27 | 4.5886 | 1.05E-05 | 0.1385 | 0.0389 |
| rs3760327 | chr17 | 34163387 | RDM1 | -4.5883 | 1.05E-05 | 0.1424 | 0.0390 |
| rs3794970 | chr19 | 58986260 | ZNF584 | -4.5878 | 1.05E-05 | 0.1357 | 0.0390 |
| rs2076204 | chr23 | 128879716 | SMARCA1 | -4.5870 | 1.06E-05 | 0.1462 | 0.0391 |
| rs3218920 | chr2 | 102634005 | IL1R2 | 4.5854 | 1.06E-05 | 0.1345 | 0.0393 |
| rs4766613 | chr12 | 110016559 | UBE3B | -4.5852 | 1.07E-05 | 0.1398 | 0.0393 |
| rs10133111 | chr14 | 103377321 | MARK3 | 4.5848 | 1.07E-05 | 0.1357 | 0.0394 |
| rs1615326 | chr16 | 58781052 | KATNB1 | 4.5831 | 1.07E-05 | 0.1347 | 0.0396 |
| rs2146340 | chr1 | 90185106 | LRRC8C | 4.5813 | 1.08E-05 | 0.1381 | 0.0399 |
| rs4984666 | chr16 | 446362 | DECR2 | -4.5807 | 1.09E-05 | 0.1429 | 0.0400 |
| rs4937055 | chr11 | 125706938 | PUS3 | 4.5799 | 1.09E-05 | 0.1341 | 0.0400 |
| rs376742 | chr20 | 25487417 | ABHD12 | 4.5799 | 1.09E-05 | 0.1508 | 0.0400 |
| rs223508 | chr4 | 103632963 | MANBA | 4.5799 | 1.09E-05 | 0.1354 | 0.0400 |
| rs3750997 | chr11 | 71158841 | NADSYN1 | 4.5790 | 1.09E-05 | 0.1438 | 0.0401 |
| rs12229020 | chr12 | 10117683 | CLEC12A | 4.5768 | 1.10E-05 | 0.1345 | 0.0405 |
| rs11622168 | chr14 | 65027753 | ZBTB1 | 4.5759 | 1.11E-05 | 0.1428 | 0.0405 |
| rs11183885 | chr12 | 47737273 | SENP1 | -4.5758 | 1.11E-05 | 0.1357 | 0.0405 |
| rs674708 | chr6 | 74217278 | SLC17A5 | 4.5758 | 1.11E-05 | 0.1339 | 0.0405 |
| rs654973 | chr6 | 74270132 | SLC17A5 | 4.5758 | 1.11E-05 | 0.1339 | 0.0405 |
| rs16879543 | chr5 | 7953365 | MTRR | 4.5741 | 1.11E-05 | 0.1400 | 0.0408 |
| rs10904771 | chr10 | 16591221 | PTER | -4.5737 | 1.12E-05 | 0.1338 | 0.0408 |
| rs351367 | chr1 | 113049862 | ST7L | -4.5726 | 1.12E-05 | 0.1394 | 0.0409 |
| rs6537742 | chr1 | 113085197 | ST7L | 4.5719 | 1.12E-05 | 0.1416 | 0.0410 |
| rs2557794 | chr2 | 171868342 | TLK1 | 4.5712 | 1.13E-05 | 0.1458 | 0.0411 |
| rs1983974 | chr20 | 25530619 | ABHD12 | 4.5709 | 1.13E-05 | 0.1506 | 0.0411 |
| rs2071487 | chr1 | 110233081 | GSTM5 | 4.5706 | 1.13E-05 | 0.2045 | 0.0411 |
| rs6584583 | chr10 | 105726338 | GSTO2 | -4.5706 | 1.13E-05 | 0.1586 | 0.0411 |
| rs9895863 | chr17 | 46007029 | MRPL10 | -4.5705 | 1.13E-05 | 0.1410 | 0.0411 |
| rs2165796 | chr17 | 2445904 | WDR81 | -4.5702 | 1.13E-05 | 0.1454 | 0.0411 |
| rs1383227 | chr18 | 445039 | TYMS | 4.5696 | 1.14E-05 | 0.1419 | 0.0412 |
| rs17284935 | chr23 | 101413759 | ARMCX6 | -4.5696 | 1.14E-05 | 0.1339 | 0.0412 |
| rs6083977 | chr20 | 25858431 | ABHD12 | -4.5695 | 1.14E-05 | 0.1562 | 0.0412 |
| rs9861286 | chr3 | 11568144 | ATG7 | 4.5692 | 1.14E-05 | 0.1453 | 0.0412 |
| rs1475642 | chr10 | 104546183 | C10orf32 | -4.5687 | 1.14E-05 | 0.1346 | 0.0412 |
| rs136575 | chr22 | 45769245 | RIBC2 | 4.5684 | 1.14E-05 | 0.1387 | 0.0413 |
| rs9773817 | chr8 | 27299277 | PNMA2 | 4.5679 | 1.14E-05 | 0.1474 | 0.0413 |
| rs2107613 | chr12 | 888428 | RAD52 | -4.5662 | 1.15E-05 | 0.1333 | 0.0416 |
| rs17051957 | chr3 | 52099441 | SEMA3G | 4.5655 | 1.15E-05 | 0.1351 | 0.0417 |
| rs4668368 | chr2 | 171926162 | TLK1 | 4.5651 | 1.16E-05 | 0.1339 | 0.0417 |
| rs12642145 | chr4 | 44834738 | GUF1 | 4.5630 | 1.17E-05 | 0.1394 | 0.0420 |
| rs4416731 | chr7 | 893637 | PRKAR1B | -4.5621 | 1.17E-05 | 0.1816 | 0.0422 |
| rs35703899 | chr5 | 113409641 | TSSK1B | 4.5616 | 1.17E-05 | 0.1340 | 0.0422 |
| rs2183557 | chr21 | 19568520 | CHODL | 4.5613 | 1.17E-05 | 0.1510 | 0.0422 |
| rs10952916 | chr7 | 87859956 | SRI | 4.5597 | 1.18E-05 | 0.1443 | 0.0425 |
| rs4303811 | chr22 | 39157755 | CBY1 | 4.5583 | 1.19E-05 | 0.1329 | 0.0427 |
| rs1706767 | chr15 | 45569099 | SLC28A2 | -4.5580 | 1.19E-05 | 0.1716 | 0.0427 |
| rs6535504 | chr4 | 84809102 | NKX6-1 | -4.5563 | 1.20E-05 | 0.1440 | 0.0430 |
| rs1833456 | chr12 | 47738848 | SENP1 | -4.5552 | 1.20E-05 | 0.1331 | 0.0432 |
| rs10408199 | chr19 | 37547659 | ZNF420 | -4.5544 | 1.21E-05 | 0.1333 | 0.0433 |
| rs10483788 | chr14 | 66757631 | FUT8 | 4.5541 | 1.21E-05 | 0.1357 | 0.0433 |
| rs7682529 | chr4 | 185477454 | ACSL1 | -4.5539 | 1.21E-05 | 0.1327 | 0.0433 |
| rs4090488 | chr17 | 66371563 | FAM20A | -4.5538 | 1.21E-05 | 0.1455 | 0.0433 |
| rs2507905 | chr11 | 115008605 | CADM1 | -4.5536 | 1.21E-05 | 0.1343 | 0.0433 |
| rs2246011 | chr9 | 130248885 | RPL12 | -4.5535 | 1.21E-05 | 0.1346 | 0.0433 |
| rs1053328 | chr16 | 85711860 | IRF8 | 4.5527 | 1.22E-05 | 0.1341 | 0.0434 |
| rs12628295 | chr22 | 37062530 | GGA1 | -4.5525 | 1.22E-05 | 0.1559 | 0.0434 |
| rs246710 | chr5 | 140528720 | PCDHB8 | -4.5522 | 1.22E-05 | 0.1367 | 0.0434 |
| rs10415555 | chr19 | 34010189 | PEPD | 4.5520 | 1.22E-05 | 0.1754 | 0.0434 |
| rs1728647 | chr7 | 105179487 | RINT1 | -4.5519 | 1.22E-05 | 0.1326 | 0.0434 |
| rs2504806 | chr6 | 39824567 | MOCS1 | -4.5508 | 1.23E-05 | 0.1390 | 0.0436 |
| rs366330 | chr2 | 178356 | SH3YL1 | -4.5507 | 1.23E-05 | 0.1325 | 0.0436 |
| rs7529194 | chr1 | 150622620 | HORMAD1 | 4.5497 | 1.23E-05 | 0.1337 | 0.0437 |
| rs7311423 | chr12 | 964670 | RAD52 | -4.5495 | 1.23E-05 | 0.1331 | 0.0437 |
| rs1071181 | chr5 | 81619915 | RPS23 | 4.5491 | 1.24E-05 | 0.1580 | 0.0437 |
| rs2300601 | chr14 | 75639571 | MLH3 | -4.5480 | 1.24E-05 | 0.1569 | 0.0439 |
| rs5912953 | chr23 | 78773099 | TBX22 | 4.5475 | 1.24E-05 | 0.1357 | 0.0440 |
| rs6498426 | chr16 | 9182577 | C16orf72 | 4.5470 | 1.25E-05 | 0.1344 | 0.0440 |
| rs2280078 | chr1 | 150600181 | HORMAD1 | -4.5470 | 1.25E-05 | 0.1323 | 0.0440 |
| rs9601084 | chr13 | 79160879 | POU4F1 | 4.5461 | 1.25E-05 | 0.1347 | 0.0441 |
| rs17064028 | chr3 | 60793400 | FHIT | 4.5456 | 1.25E-05 | 0.1348 | 0.0442 |
| rs4285396 | chr7 | 1123738 | ZFAND2A | 4.5450 | 1.26E-05 | 0.1326 | 0.0443 |
| rs2727330 | chr17 | 61925498 | FTSJ3 | -4.5443 | 1.26E-05 | 0.1422 | 0.0444 |
| rs6577142 | chr1 | 100351635 | SASS6 | -4.5432 | 1.27E-05 | 0.1338 | 0.0445 |
| rs12294378 | chr11 | 34434761 | ABTB2 | -4.5425 | 1.27E-05 | 0.1323 | 0.0446 |
| rs7145159 | chr14 | 75583268 | EIF2B2 | -4.5423 | 1.27E-05 | 0.1440 | 0.0446 |
| rs2384974 | chr16 | 651279 | WFIKKN1 | -4.5422 | 1.27E-05 | 0.1377 | 0.0446 |
| rs11209479 | chr1 | 69965403 | HHLA3 | -4.5414 | 1.27E-05 | 0.1379 | 0.0447 |
| rs13017295 | chr2 | 204977614 | ICOS | 4.5397 | 1.28E-05 | 0.1344 | 0.0450 |
| rs4445669 | chr11 | 115045237 | CADM1 | -4.5384 | 1.29E-05 | 0.1337 | 0.0452 |
| rs11205911 | chr1 | 52396312 | RAB3B | 4.5381 | 1.29E-05 | 0.1355 | 0.0452 |
| rs4954231 | chr2 | 136016514 | CCNT2 | -4.5378 | 1.29E-05 | 0.1326 | 0.0452 |
| rs6523235 | chr23 | 78914942 | TBX22 | 4.5378 | 1.29E-05 | 0.1353 | 0.0452 |
| rs7780365 | chr7 | 31438610 | NEUROD6 | 4.5362 | 1.30E-05 | 0.1318 | 0.0455 |
| rs17575480 | chr12 | 45156678 | NELL2 | 4.5359 | 1.30E-05 | 0.1332 | 0.0455 |
| rs6813 | chr17 | 19808445 | AKAP10 | 4.5358 | 1.30E-05 | 0.1442 | 0.0455 |
| rs2330745 | chr7 | 43195455 | HECW1 | -4.5349 | 1.31E-05 | 0.1317 | 0.0456 |
| rs10507165 | chr12 | 103856813 | PAH | 4.5347 | 1.31E-05 | 0.1357 | 0.0456 |
| rs1336900 | chr1 | 150679033 | HORMAD1 | 4.5346 | 1.31E-05 | 0.1325 | 0.0456 |
| rs10948728 | chr6 | 52723635 | GSTA3 | -4.5340 | 1.31E-05 | 0.1423 | 0.0457 |
| rs2144694 | chr6 | 52604350 | GSTA3 | -4.5339 | 1.31E-05 | 0.1323 | 0.0457 |
| rs7485576 | chr12 | 75692607 | GLIPR1L2 | -4.5336 | 1.32E-05 | 0.1324 | 0.0457 |
| rs1004770 | chr17 | 45917147 | MRPL10 | 4.5326 | 1.32E-05 | 0.1648 | 0.0459 |
| rs11654099 | chr17 | 6364753 | TXNDC17 | 4.5316 | 1.33E-05 | 0.1396 | 0.0460 |
| rs223510 | chr4 | 103627968 | MANBA | 4.5313 | 1.33E-05 | 0.1410 | 0.0461 |
| rs529587 | chr1 | 15430228 | TMEM51 | 4.5311 | 1.33E-05 | 0.1432 | 0.0461 |
| rs17574839 | chr12 | 45108480 | NELL2 | 4.5306 | 1.33E-05 | 0.1371 | 0.0461 |
| rs4729594 | chr7 | 77298752 | TMEM60 | -4.5306 | 1.33E-05 | 0.1317 | 0.0461 |
| rs6979076 | chr7 | 1116247 | ZFAND2A | 4.5305 | 1.33E-05 | 0.1333 | 0.0461 |
| rs10414048 | chr19 | 43607205 | ZNF526 | 4.5303 | 1.33E-05 | 0.1339 | 0.0461 |
| rs11663269 | chr18 | 33205797 | MOCOS | -4.5297 | 1.34E-05 | 0.1339 | 0.0462 |
| rs1321000 | chr20 | 44801026 | UBE2C | -4.5289 | 1.34E-05 | 0.1416 | 0.0463 |
| rs7338828 | chr13 | 104066713 | KDELC1 | 4.5286 | 1.34E-05 | 0.1521 | 0.0463 |
| rs6521151 | chr23 | 46636971 | RBM10 | -4.5280 | 1.35E-05 | 0.1324 | 0.0464 |
| rs9921035 | chr15 | 45546660 | SLC28A2 | -4.5279 | 1.35E-05 | 0.1734 | 0.0464 |
| rs4411798 | chr21 | 38453183 | PIGP | -4.5276 | 1.35E-05 | 0.1363 | 0.0464 |
| rs1697754 | chr12 | 75893865 | GLIPR1L2 | 4.5273 | 1.35E-05 | 0.1339 | 0.0464 |
| rs1112308 | chr16 | 20629585 | ACSM1 | 4.5271 | 1.35E-05 | 0.1338 | 0.0464 |
| rs753279 | chr5 | 140023818 | ZMAT2 | -4.5271 | 1.35E-05 | 0.1360 | 0.0464 |
| rs223467 | chr4 | 103700878 | MANBA | 4.5270 | 1.35E-05 | 0.1337 | 0.0464 |
| rs4769547 | chr13 | 27802956 | LNX2 | 4.5249 | 1.36E-05 | 0.1372 | 0.0468 |
| rs4255987 | chr2 | 259296 | SH3YL1 | 4.5233 | 1.37E-05 | 0.1387 | 0.0470 |
| rs7772706 | chr6 | 106601936 | PREP | -4.5232 | 1.37E-05 | 0.1391 | 0.0470 |
| rs2293293 | chr3 | 134322742 | ANAPC13 | -4.5222 | 1.38E-05 | 0.1318 | 0.0472 |
| rs10492391 | chr13 | 32742925 | KL | 4.5216 | 1.38E-05 | 0.1329 | 0.0473 |
| rs4815398 | chr20 | 25237338 | ABHD12 | 4.5214 | 1.38E-05 | 0.1374 | 0.0473 |
| rs10733139 | chr1 | 15996555 | SLC25A34 | -4.5196 | 1.39E-05 | 0.1357 | 0.0476 |
| rs3112524 | chr17 | 16245778 | ZSWIM7 | 4.5193 | 1.39E-05 | 0.1782 | 0.0476 |
| rs17199131 | chr12 | 76963662 | BBS10 | -4.5192 | 1.40E-05 | 0.1366 | 0.0476 |
| rs9295308 | chr6 | 158537842 | SYNJ2 | -4.5174 | 1.41E-05 | 0.1308 | 0.0479 |
| rs8037583 | chr15 | 45546378 | SLC28A2 | -4.5174 | 1.41E-05 | 0.1427 | 0.0479 |
| rs8006587 | chr14 | 75664939 | MLH3 | -4.5173 | 1.41E-05 | 0.1456 | 0.0479 |
| rs1063392 | chr22 | 42454950 | CYP2D6 | -4.5170 | 1.41E-05 | 0.1327 | 0.0479 |
| rs2156076 | chr21 | 38452634 | PIGP | -4.5170 | 1.41E-05 | 0.1448 | 0.0479 |
| rs8080555 | chr17 | 66371273 | FAM20A | -4.5165 | 1.41E-05 | 0.1332 | 0.0479 |
| rs4523733 | chr12 | 48927514 | TUBA1C | 4.5161 | 1.41E-05 | 0.1476 | 0.0480 |
| rs12466395 | chr2 | 190780698 | ORMDL1 | 4.5160 | 1.41E-05 | 0.1352 | 0.0480 |
| rs920921 | chr15 | 68786285 | ITGA11 | 4.5154 | 1.42E-05 | 0.1400 | 0.0480 |
| rs2744572 | chr6 | 24490511 | DCDC2 | 4.5154 | 1.42E-05 | 0.1344 | 0.0480 |
| rs9393713 | chr6 | 26373678 | BTN3A2 | -4.5150 | 1.42E-05 | 0.1307 | 0.0480 |
| rs1978 | chr6 | 26377573 | BTN3A2 | -4.5150 | 1.42E-05 | 0.1307 | 0.0480 |
| rs376206 | chr20 | 44377039 | WFDC3 | -4.5145 | 1.42E-05 | 0.1807 | 0.0481 |
| rs17648414 | chr5 | 177851690 | COL23A1 | -4.5145 | 1.42E-05 | 0.1364 | 0.0481 |
| rs7695701 | chr4 | 57190356 | PPAT | -4.5137 | 1.43E-05 | 0.1520 | 0.0482 |
| rs620730 | chr23 | 112073165 | TRPC5 | 4.5132 | 1.43E-05 | 0.1306 | 0.0483 |
| rs10849538 | chr12 | 7014607 | SPSB2 | 4.5128 | 1.43E-05 | 0.1394 | 0.0483 |
| rs17258091 | chr23 | 101174035 | ARMCX6 | -4.5123 | 1.43E-05 | 0.1315 | 0.0483 |
| rs4866796 | chr5 | 42744895 | C5orf34 | 4.5123 | 1.43E-05 | 0.1378 | 0.0483 |
| rs2581922 | chr11 | 57265400 | TIMM10 | 4.5117 | 1.44E-05 | 0.1340 | 0.0484 |
| rs555996 | chr9 | 124396311 | STOM | 4.5115 | 1.44E-05 | 0.1306 | 0.0484 |
| rs351187 | chr15 | 74595812 | ARID3B | 4.5107 | 1.44E-05 | 0.1390 | 0.0486 |
| rs2274517 | chr6 | 42932715 | GNMT | 4.5086 | 1.46E-05 | 0.1375 | 0.0489 |
| rs352148 | chr3 | 52208898 | SEMA3G | 4.5082 | 1.46E-05 | 0.1334 | 0.0490 |
| rs10167387 | chr2 | 201808618 | CFLAR | -4.5046 | 1.48E-05 | 0.1352 | 0.0497 |
| rs2964543 | chr5 | 114623745 | PGGT1B | -4.5044 | 1.48E-05 | 0.1446 | 0.0497 |
| rs2403279 | chr11 | 18416615 | LDHC | 4.5031 | 1.49E-05 | 0.1423 | 0.0499 |
| rs11903909 | chr2 | 106589441 | ST6GAL2 | -4.5023 | 1.49E-05 | 0.1300 | 0.0500 |
| rs158570 | chr5 | 60224914 | ERCC8 | 4.5003 | 1.51E-05 | 0.1444 | 0.0504 |
| rs42873 | chr16 | 23694732 | DCTN5 | -4.4995 | 1.51E-05 | 0.1329 | 0.0505 |
| rs7648255 | chr3 | 122167680 | CCDC58 | 4.4995 | 1.51E-05 | 0.1306 | 0.0505 |
| rs17091314 | chr23 | 152826592 | PNMA5 | 4.4992 | 1.51E-05 | 0.1309 | 0.0505 |
| rs12308045 | chr12 | 53165178 | CSAD | 4.4988 | 1.52E-05 | 0.1299 | 0.0506 |
| rs11197775 | chr10 | 118396138 | PNLIPRP2 | 4.4977 | 1.52E-05 | 0.1388 | 0.0508 |
| rs4766601 | chr12 | 109890080 | UBE3B | 4.4973 | 1.53E-05 | 0.1344 | 0.0508 |
| rs1026049 | chr4 | 140430208 | RAB33B | -4.4970 | 1.53E-05 | 0.1336 | 0.0509 |
| rs6957709 | chr7 | 1171730 | ZFAND2A | -4.4959 | 1.53E-05 | 0.1350 | 0.0510 |
| rs7521001 | chr1 | 52354382 | RAB3B | 4.4959 | 1.53E-05 | 0.1340 | 0.0510 |
| rs10056565 | chr5 | 80619046 | RASGRF2 | 4.4950 | 1.54E-05 | 0.1417 | 0.0512 |
| rs4903289 | chr14 | 75623988 | MLH3 | -4.4926 | 1.55E-05 | 0.1371 | 0.0516 |
| rs17774144 | chr17 | 45904886 | MRPL10 | 4.4904 | 1.57E-05 | 0.1353 | 0.0521 |
| rs2670026 | chr8 | 57424338 | PENK | 4.4903 | 1.57E-05 | 0.1608 | 0.0521 |
| rs6553951 | chr4 | 177308667 | GPM6A | 4.4900 | 1.57E-05 | 0.1422 | 0.0521 |
| rs8021125 | chr14 | 81704787 | GTF2A1 | 4.4897 | 1.57E-05 | 0.1458 | 0.0521 |
| rs11190749 | chr10 | 102665261 | MRPL43 | 4.4895 | 1.57E-05 | 0.1313 | 0.0521 |
| rs119672 | chr17 | 19818601 | AKAP10 | 4.4882 | 1.58E-05 | 0.1558 | 0.0524 |
| rs982848 | chr16 | 58738198 | KATNB1 | 4.4880 | 1.58E-05 | 0.1292 | 0.0524 |
| rs7904890 | chr10 | 88776702 | BMPR1A | 4.4870 | 1.59E-05 | 0.1422 | 0.0525 |
| rs16966998 | chr18 | 33259637 | MOCOS | -4.4858 | 1.60E-05 | 0.1293 | 0.0528 |
| rs4268717 | chr15 | 78600568 | CHRNA5 | -4.4849 | 1.60E-05 | 0.1393 | 0.0529 |
| rs17716041 | chr4 | 88832384 | SPP1 | -4.4825 | 1.62E-05 | 0.1315 | 0.0534 |
| rs12023402 | chr1 | 162252348 | NOS1AP | 4.4821 | 1.62E-05 | 0.1289 | 0.0535 |
| rs4002957 | chr8 | 101988226 | YWHAZ | -4.4819 | 1.62E-05 | 0.1297 | 0.0535 |
| rs7112492 | chr11 | 18405510 | LDHC | 4.4811 | 1.63E-05 | 0.1450 | 0.0536 |
| rs6917325 | chr6 | 52666273 | GSTA5 | -4.4809 | 1.63E-05 | 0.1381 | 0.0536 |
| rs1570544 | chr13 | 103472072 | BIVM | 4.4807 | 1.63E-05 | 0.1493 | 0.0536 |
| rs7670601 | chr4 | 44715341 | GUF1 | 4.4805 | 1.63E-05 | 0.1334 | 0.0536 |
| rs12866387 | chr13 | 44391936 | CCDC122 | 4.4801 | 1.64E-05 | 0.1379 | 0.0537 |
| rs6895004 | chr5 | 73724543 | FOXD1 | 4.4801 | 1.64E-05 | 0.1291 | 0.0537 |
| rs12923097 | chr16 | 84143565 | HSDL1 | -4.4795 | 1.64E-05 | 0.1301 | 0.0537 |
| rs2936840 | chr12 | 118578843 | PEBP1 | -4.4792 | 1.64E-05 | 0.1307 | 0.0538 |
| rs4866954 | chr5 | 42747162 | C5orf34 | 4.4790 | 1.64E-05 | 0.1343 | 0.0538 |
| rs6429757 | chr1 | 15909850 | CASP9 | -4.4783 | 1.65E-05 | 0.1461 | 0.0539 |
| rs954302 | chr1 | 69951009 | HHLA3 | -4.4777 | 1.65E-05 | 0.1325 | 0.0540 |
| rs7800269 | chr7 | 42428478 | HECW1 | -4.4768 | 1.66E-05 | 0.1400 | 0.0541 |
| rs1969964 | chr9 | 34155112 | NUDT2 | -4.4767 | 1.66E-05 | 0.1365 | 0.0541 |
| rs3817731 | chr20 | 44518843 | WFDC3 | -4.4767 | 1.66E-05 | 0.1386 | 0.0541 |
| rs11214589 | chr11 | 113245048 | TTC12 | 4.4762 | 1.66E-05 | 0.1344 | 0.0542 |
| rs171786 | chr16 | 58761450 | KATNB1 | 4.4759 | 1.66E-05 | 0.1485 | 0.0542 |
| rs629168 | chr5 | 140575211 | PCDHB8 | -4.4759 | 1.66E-05 | 0.1305 | 0.0542 |
| rs7295704 | chr12 | 884889 | RAD52 | -4.4758 | 1.66E-05 | 0.1497 | 0.0542 |
| rs2046910 | chr4 | 44653239 | GUF1 | 4.4756 | 1.67E-05 | 0.1403 | 0.0542 |
| rs6107060 | chr20 | 25592476 | NANP | 4.4747 | 1.67E-05 | 0.1575 | 0.0543 |
| rs2044029 | chr15 | 71679959 | LARP6 | -4.4740 | 1.68E-05 | 0.1300 | 0.0544 |
| rs3793735 | chr10 | 1282828 | ADARB2 | 4.4731 | 1.68E-05 | 0.1289 | 0.0546 |
| rs527721 | chr11 | 125600594 | PUS3 | -4.4730 | 1.68E-05 | 0.1284 | 0.0546 |
| rs295573 | chr5 | 60426260 | ERCC8 | 4.4724 | 1.69E-05 | 0.1608 | 0.0547 |
| rs3742778 | chr14 | 75538217 | MLH3 | -4.4724 | 1.69E-05 | 0.1480 | 0.0547 |
| rs13360035 | chr5 | 36087026 | SKP2 | -4.4719 | 1.69E-05 | 0.1292 | 0.0547 |
| rs12620435 | chr2 | 201995779 | PPIL3 | -4.4719 | 1.69E-05 | 0.1284 | 0.0547 |
| rs380691 | chr5 | 79952034 | DHFR | 4.4718 | 1.69E-05 | 0.1307 | 0.0547 |
| rs17012821 | chr12 | 85767668 | NTS | 4.4708 | 1.70E-05 | 0.1311 | 0.0549 |
| rs3821689 | chr3 | 53208917 | PBRM1 | 4.4703 | 1.70E-05 | 0.1344 | 0.0550 |
| rs11215416 | chr11 | 115077327 | CADM1 | -4.4701 | 1.70E-05 | 0.1312 | 0.0550 |
| rs12447788 | chr16 | 9215165 | C16orf72 | 4.4692 | 1.71E-05 | 0.1282 | 0.0551 |
| rs9460143 | chr6 | 170717215 | TBP | 4.4690 | 1.71E-05 | 0.1347 | 0.0551 |
| rs17652715 | chr12 | 45092660 | NELL2 | 4.4690 | 1.71E-05 | 0.1283 | 0.0551 |
| rs555297 | chr11 | 125599866 | PUS3 | -4.4685 | 1.71E-05 | 0.1282 | 0.0551 |
| rs6985891 | chr8 | 103921795 | ODF1 | 4.4685 | 1.71E-05 | 0.1450 | 0.0551 |
| rs902696 | chr2 | 113954879 | PAX8 | -4.4685 | 1.71E-05 | 0.1281 | 0.0551 |
| rs559032 | chr11 | 68710847 | MRPL21 | -4.4677 | 1.72E-05 | 0.1287 | 0.0553 |
| rs6586030 | chr10 | 82254047 | TSPAN14 | 4.4675 | 1.72E-05 | 0.1282 | 0.0553 |
| rs2195072 | chr2 | 172028746 | TLK1 | 4.4673 | 1.72E-05 | 0.1289 | 0.0553 |
| rs2735515 | chr16 | 3200660 | ZNF213 | -4.4667 | 1.73E-05 | 0.1326 | 0.0554 |
| rs1333647 | chr1 | 36161383 | C1orf216 | -4.4662 | 1.73E-05 | 0.1280 | 0.0554 |
| rs1638527 | chr17 | 19845393 | AKAP10 | 4.4661 | 1.73E-05 | 0.1376 | 0.0554 |
| rs10489673 | chr1 | 157550066 | FCRL3 | -4.4656 | 1.73E-05 | 0.1282 | 0.0555 |
| rs7322 | chr15 | 65255704 | SPG21 | -4.4654 | 1.74E-05 | 0.1393 | 0.0555 |
| rs2144694 | chr6 | 52604350 | GSTA5 | -4.4645 | 1.74E-05 | 0.1285 | 0.0557 |
| rs7928249 | chr11 | 71161063 | NADSYN1 | 4.4640 | 1.75E-05 | 0.1279 | 0.0558 |
| rs2280724 | chr7 | 1064720 | ZFAND2A | 4.4627 | 1.75E-05 | 0.1376 | 0.0560 |
| rs11692330 | chr2 | 210076 | SH3YL1 | 4.4622 | 1.76E-05 | 0.1327 | 0.0561 |
| rs2097429 | chr22 | 46256480 | PHF21B | 4.4604 | 1.77E-05 | 0.1301 | 0.0565 |
| rs10775191 | chr15 | 67090870 | TIPIN | -4.4601 | 1.77E-05 | 0.1327 | 0.0565 |
| rs4955526 | chr3 | 134317337 | ANAPC13 | -4.4600 | 1.77E-05 | 0.1287 | 0.0565 |
| rs1450685 | chr9 | 106937745 | SMC2 | -4.4595 | 1.78E-05 | 0.1301 | 0.0566 |
| rs10418550 | chr19 | 10719482 | CARM1 | -4.4592 | 1.78E-05 | 0.1277 | 0.0566 |
| rs1691341 | chr6 | 74319303 | SLC17A5 | 4.4589 | 1.78E-05 | 0.1277 | 0.0566 |
| rs527606 | chr6 | 74346172 | SLC17A5 | 4.4589 | 1.78E-05 | 0.1277 | 0.0566 |
| rs10417171 | chr19 | 34386229 | LSM14A | -4.4588 | 1.78E-05 | 0.1294 | 0.0566 |
| rs7122402 | chr11 | 115016307 | CADM1 | -4.4581 | 1.79E-05 | 0.1298 | 0.0567 |
| rs12164334 | chr23 | 100974000 | ARMCX3 | -4.4578 | 1.79E-05 | 0.1276 | 0.0567 |
| rs10789942 | chr11 | 113236199 | TTC12 | 4.4578 | 1.79E-05 | 0.1287 | 0.0567 |
| rs6130959 | chr20 | 44501458 | WFDC3 | -4.4568 | 1.80E-05 | 0.1406 | 0.0569 |
| rs9315093 | chr13 | 31585928 | EEF1DP3 | -4.4566 | 1.80E-05 | 0.1335 | 0.0569 |
| rs729784 | chr12 | 109981264 | UBE3B | -4.4559 | 1.80E-05 | 0.1297 | 0.0570 |
| rs2910324 | chr5 | 140570199 | PCDHB8 | -4.4543 | 1.81E-05 | 0.1292 | 0.0574 |
| rs4792721 | chr17 | 16006827 | ZSWIM7 | 4.4539 | 1.82E-05 | 0.1413 | 0.0574 |
| rs10502290 | chr18 | 675903 | ENOSF1 | 4.4531 | 1.82E-05 | 0.1343 | 0.0576 |
| rs11778699 | chr8 | 29423230 | RBPMS | -4.4529 | 1.82E-05 | 0.1362 | 0.0576 |
| rs11649508 | chr16 | 3987523 | MEFV | -4.4528 | 1.83E-05 | 0.1284 | 0.0576 |
| rs848481 | chr7 | 77545104 | TMEM60 | -4.4524 | 1.83E-05 | 0.1273 | 0.0576 |
| rs867198 | chr22 | 38708606 | CSNK1E | -4.4511 | 1.84E-05 | 0.1479 | 0.0579 |
| rs7031998 | chr9 | 4490134 | SLC1A1 | -4.4506 | 1.84E-05 | 0.1416 | 0.0580 |
| rs257621 | chr16 | 58761580 | KATNB1 | -4.4493 | 1.85E-05 | 0.1297 | 0.0582 |
| rs4919690 | chr10 | 104616500 | C10orf32 | -4.4493 | 1.85E-05 | 0.1326 | 0.0582 |
| rs680355 | chr22 | 25918709 | CRYBB2 | 4.4492 | 1.85E-05 | 0.1598 | 0.0582 |
| rs11627442 | chr14 | 75669649 | MLH3 | -4.4489 | 1.85E-05 | 0.1409 | 0.0582 |
| rs3759352 | chr12 | 7012055 | SPSB2 | 4.4487 | 1.86E-05 | 0.1653 | 0.0583 |
| rs7048305 | chr9 | 116189948 | C9orf43 | -4.4484 | 1.86E-05 | 0.1344 | 0.0583 |
| rs16831873 | chr2 | 190556898 | ORMDL1 | 4.4479 | 1.86E-05 | 0.1280 | 0.0584 |
| rs6838327 | chr4 | 44626846 | GUF1 | 4.4468 | 1.87E-05 | 0.1405 | 0.0586 |
| rs2466421 | chr19 | 44819582 | ZNF227 | -4.4462 | 1.87E-05 | 0.1400 | 0.0587 |
| rs462145 | chr20 | 44378211 | WFDC10B | 4.4442 | 1.89E-05 | 0.1445 | 0.0591 |
| rs3996993 | chr6 | 52725521 | GSTA5 | -4.4441 | 1.89E-05 | 0.1282 | 0.0591 |
| rs2098058 | chr5 | 140157427 | ZMAT2 | -4.4438 | 1.89E-05 | 0.1309 | 0.0592 |
| rs673753 | chr11 | 65206017 | DPF2 | -4.4430 | 1.90E-05 | 0.1319 | 0.0593 |
| rs12627745 | chr21 | 46797389 | COL18A1 | 4.4421 | 1.91E-05 | 0.1293 | 0.0595 |
| rs2012627 | chr14 | 75443103 | EIF2B2 | -4.4412 | 1.91E-05 | 0.1367 | 0.0597 |
| rs7995465 | chr13 | 103424331 | BIVM | 4.4410 | 1.91E-05 | 0.1311 | 0.0597 |
| rs6421653 | chr11 | 68719598 | MRPL21 | -4.4407 | 1.92E-05 | 0.1307 | 0.0597 |
| rs4647575 | chr11 | 65107169 | DPF2 | -4.4398 | 1.92E-05 | 0.1326 | 0.0599 |
| rs3764396 | chr17 | 46015969 | MRPL10 | -4.4395 | 1.93E-05 | 0.1326 | 0.0599 |
| rs12224788 | chr11 | 125329061 | PUS3 | -4.4393 | 1.93E-05 | 0.1267 | 0.0599 |
| rs2236927 | chr2 | 102634984 | IL1R2 | 4.4385 | 1.93E-05 | 0.1279 | 0.0601 |
| rs17565659 | chr13 | 27411229 | RNF6 | -4.4378 | 1.94E-05 | 0.1276 | 0.0602 |
| rs7735365 | chr5 | 140570472 | PCDHB8 | -4.4374 | 1.94E-05 | 0.1330 | 0.0602 |
| rs12717860 | chr5 | 140171442 | ZMAT2 | -4.4373 | 1.94E-05 | 0.1303 | 0.0602 |
| rs11815919 | chr10 | 44831379 | ZNF239 | -4.4373 | 1.94E-05 | 0.1267 | 0.0602 |
| rs17279498 | chr23 | 150467271 | MAGEA4 | -4.4366 | 1.95E-05 | 0.1270 | 0.0604 |
| rs17032053 | chr2 | 106574745 | ST6GAL2 | -4.4358 | 1.96E-05 | 0.1265 | 0.0605 |
| rs2505133 | chr10 | 30325539 | MAP3K8 | 4.4348 | 1.96E-05 | 0.1264 | 0.0607 |
| rs8012716 | chr14 | 21202336 | RNASE1 | -4.4346 | 1.96E-05 | 0.1367 | 0.0607 |
| rs2709373 | chr2 | 208386024 | CREB1 | 4.4345 | 1.96E-05 | 0.1264 | 0.0607 |
| rs17398173 | chr9 | 85648814 | RASEF | -4.4340 | 1.97E-05 | 0.1264 | 0.0608 |
| rs16842071 | chr2 | 201931730 | CFLAR | -4.4332 | 1.98E-05 | 0.1359 | 0.0610 |
| rs11704481 | chr22 | 45732328 | RIBC2 | 4.4328 | 1.98E-05 | 0.1385 | 0.0610 |
| rs1078763 | chr2 | 113763 | SH3YL1 | -4.4322 | 1.98E-05 | 0.1430 | 0.0611 |
| rs4934282 | chr10 | 88732476 | BMPR1A | 4.4316 | 1.99E-05 | 0.1531 | 0.0612 |
| rs11906429 | chr20 | 44432193 | WFDC10B | 4.4313 | 1.99E-05 | 0.1368 | 0.0613 |
| rs311856 | chr14 | 59205486 | TIMM9 | 4.4306 | 2.00E-05 | 0.1262 | 0.0614 |
| rs10136732 | chr14 | 64950973 | ZBTB1 | -4.4302 | 2.00E-05 | 0.1411 | 0.0615 |
| rs7193136 | chr16 | 4580034 | CORO7 | -4.4295 | 2.01E-05 | 0.1306 | 0.0616 |
| rs10407110 | chr19 | 52036639 | SIGLEC12 | 4.4287 | 2.01E-05 | 0.1260 | 0.0618 |
| rs26760 | chr16 | 23680286 | DCTN5 | -4.4280 | 2.02E-05 | 0.1272 | 0.0619 |
| rs2078106 | chr2 | 18058393 | KCNS3 | 4.4275 | 2.02E-05 | 0.1279 | 0.0620 |
| rs10413398 | chr19 | 2106696 | PCSK4 | -4.4274 | 2.02E-05 | 0.1303 | 0.0620 |
| rs6806417 | chr3 | 134211446 | ANAPC13 | -4.4265 | 2.03E-05 | 0.1317 | 0.0621 |
| rs9614462 | chr22 | 45751231 | RIBC2 | 4.4264 | 2.03E-05 | 0.1292 | 0.0621 |
| rs2526477 | chr17 | 20131193 | AKAP10 | -4.4260 | 2.03E-05 | 0.1516 | 0.0622 |
| rs735452 | chr14 | 75497984 | MLH3 | -4.4250 | 2.04E-05 | 0.1460 | 0.0623 |
| rs12027327 | chr1 | 114664905 | OLFML3 | 4.4250 | 2.04E-05 | 0.1396 | 0.0623 |
| rs7299563 | chr12 | 76769209 | BBS10 | 4.4250 | 2.04E-05 | 0.1264 | 0.0623 |
| rs17197538 | chr12 | 76822661 | BBS10 | 4.4250 | 2.04E-05 | 0.1264 | 0.0623 |
| rs2194337 | chr16 | 3205335 | ZNF213 | -4.4245 | 2.05E-05 | 0.1259 | 0.0624 |
| rs10784673 | chr12 | 68487460 | NUP107 | -4.4240 | 2.05E-05 | 0.1585 | 0.0625 |
| rs390409 | chr20 | 44381615 | WFDC10B | 4.4229 | 2.06E-05 | 0.1366 | 0.0627 |
| rs7997347 | chr13 | 48624890 | MED4 | -4.4229 | 2.06E-05 | 0.1290 | 0.0627 |
| rs12692412 | chr2 | 10720054 | NOL10 | -4.4223 | 2.06E-05 | 0.1269 | 0.0627 |
| rs11125107 | chr2 | 47192397 | MCFD2 | 4.4223 | 2.06E-05 | 0.1261 | 0.0627 |
| rs1951614 | chr14 | 81672106 | GTF2A1 | 4.4222 | 2.06E-05 | 0.1400 | 0.0627 |
| rs163255 | chr16 | 20689815 | ACSM1 | 4.4221 | 2.07E-05 | 0.1260 | 0.0627 |
| rs2592178 | chr2 | 70472482 | FAM136A | -4.4208 | 2.08E-05 | 0.1326 | 0.0630 |
| rs966770 | chr4 | 30451158 | PCDH7 | 4.4207 | 2.08E-05 | 0.1257 | 0.0630 |
| rs9646413 | chr17 | 41426157 | NBR2 | -4.4181 | 2.10E-05 | 0.1481 | 0.0636 |
| rs457570 | chr20 | 44371922 | WFDC10B | 4.4179 | 2.10E-05 | 0.1386 | 0.0636 |
| rs6694039 | chr1 | 90223073 | LRRC8C | 4.4175 | 2.10E-05 | 0.1285 | 0.0637 |
| rs9857242 | chr3 | 134316378 | ANAPC13 | -4.4169 | 2.11E-05 | 0.1260 | 0.0638 |
| rs6746170 | chr2 | 44228381 | PPM1B | 4.4151 | 2.12E-05 | 0.1338 | 0.0643 |
| rs12354855 | chr10 | 127394711 | FAM53B | -4.4144 | 2.13E-05 | 0.1257 | 0.0644 |
| rs10894888 | chr11 | 134694677 | B3GAT1 | 4.4137 | 2.14E-05 | 0.1257 | 0.0645 |
| rs7842037 | chr8 | 21067151 | LGI3 | -4.4135 | 2.14E-05 | 0.1255 | 0.0645 |
| rs9349639 | chr6 | 52538242 | PAQR8 | 4.4134 | 2.14E-05 | 0.1263 | 0.0645 |
| rs3785628 | chr17 | 15970682 | ZSWIM7 | 4.4133 | 2.14E-05 | 0.1420 | 0.0645 |
| rs3759352 | chr12 | 7012055 | LRRC23 | 4.4125 | 2.15E-05 | 0.1598 | 0.0647 |
| rs4938179 | chr11 | 115033425 | CADM1 | -4.4120 | 2.15E-05 | 0.1258 | 0.0648 |
| rs4802703 | chr19 | 50884885 | NAPSA | -4.4115 | 2.16E-05 | 0.1291 | 0.0649 |
| rs17261508 | chr7 | 37457933 | ELMO1 | 4.4113 | 2.16E-05 | 0.1265 | 0.0649 |
| rs765250 | chr12 | 908283 | RAD52 | -4.4106 | 2.16E-05 | 0.1278 | 0.0650 |
| rs4657454 | chr1 | 165563940 | MGST3 | -4.4099 | 2.17E-05 | 0.1277 | 0.0652 |
| rs2072478 | chr2 | 102636526 | IL1R2 | 4.4097 | 2.17E-05 | 0.1252 | 0.0652 |
| rs12422045 | chr11 | 71154820 | NADSYN1 | 4.4082 | 2.18E-05 | 0.1278 | 0.0655 |
| rs6877 | chr23 | 149935100 | MTMR1 | 4.4082 | 2.18E-05 | 0.1354 | 0.0655 |
| rs736894 | chr11 | 71152258 | NADSYN1 | 4.4081 | 2.19E-05 | 0.1313 | 0.0655 |
| rs7090425 | chr10 | 1203168 | ZMYND11 | 4.4078 | 2.19E-05 | 0.1330 | 0.0655 |
| rs2504793 | chr6 | 39842914 | MOCS1 | -4.4053 | 2.21E-05 | 0.1290 | 0.0662 |
| rs13220304 | chr6 | 109652236 | CD164 | 4.4046 | 2.22E-05 | 0.1249 | 0.0663 |
| rs444197 | chr9 | 135558503 | GTF3C4 | -4.4035 | 2.23E-05 | 0.1364 | 0.0665 |
| rs12889472 | chr14 | 75543979 | EIF2B2 | -4.4034 | 2.23E-05 | 0.1516 | 0.0665 |
| rs2282745 | chr2 | 102632064 | IL1R2 | 4.4033 | 2.23E-05 | 0.1247 | 0.0665 |
| rs11903198 | chr2 | 20880345 | MATN3 | 4.4033 | 2.23E-05 | 0.1311 | 0.0665 |
| rs6759555 | chr2 | 75881828 | MRPL19 | -4.4018 | 2.24E-05 | 0.1247 | 0.0668 |
| rs3852689 | chr16 | 69355673 | SNTB2 | 4.4015 | 2.24E-05 | 0.1353 | 0.0669 |
| rs1034904 | chr17 | 19927699 | AKAP10 | 4.4009 | 2.25E-05 | 0.2297 | 0.0670 |
| rs584108 | chr11 | 68630282 | MRPL21 | -4.4004 | 2.25E-05 | 0.1277 | 0.0670 |
| rs7140186 | chr14 | 34594507 | NPAS3 | -4.4004 | 2.25E-05 | 0.1261 | 0.0670 |
| rs2268619 | chr14 | 75640354 | MLH3 | -4.4003 | 2.25E-05 | 0.1487 | 0.0670 |
| rs4852506 | chr2 | 70554285 | FAM136A | -4.4003 | 2.25E-05 | 0.1281 | 0.0670 |
| rs17112175 | chr10 | 98616993 | SFRP5 | -4.3999 | 2.26E-05 | 0.1440 | 0.0670 |
| rs3218872 | chr2 | 102628509 | IL1R2 | 4.3998 | 2.26E-05 | 0.1246 | 0.0670 |
| rs2072477 | chr2 | 102636759 | IL1R2 | 4.3998 | 2.26E-05 | 0.1246 | 0.0670 |
| rs2072476 | chr2 | 102636864 | IL1R2 | 4.3998 | 2.26E-05 | 0.1246 | 0.0670 |
| rs11165030 | chr1 | 94349077 | DR1 | 4.3996 | 2.26E-05 | 0.1273 | 0.0670 |
| rs4976639 | chr5 | 176550301 | PRELID1 | -4.3995 | 2.26E-05 | 0.1462 | 0.0670 |
| rs2063690 | chr15 | 66641732 | MAP2K1 | 4.3990 | 2.27E-05 | 0.1280 | 0.0671 |
| rs6993645 | chr8 | 28933444 | HMBOX1 | -4.3966 | 2.29E-05 | 0.1347 | 0.0677 |
| rs1930961 | chr22 | 25875265 | CRYBB2 | 4.3966 | 2.29E-05 | 0.1574 | 0.0677 |
| rs16957244 | chr15 | 43166029 | PLA2G4E | -4.3960 | 2.29E-05 | 0.1258 | 0.0678 |
| rs2057754 | chr22 | 19037535 | DGCR14 | 4.3957 | 2.30E-05 | 0.1243 | 0.0678 |
| rs11078889 | chr17 | 11147540 | MYH2 | 4.3951 | 2.30E-05 | 0.1285 | 0.0679 |
| rs510757 | chr19 | 37422802 | ZNF420 | -4.3939 | 2.31E-05 | 0.1259 | 0.0682 |
| rs516316 | chr19 | 49206145 | FCGRT | -4.3939 | 2.31E-05 | 0.1279 | 0.0682 |
| rs2791728 | chr9 | 133611739 | GPR107 | 4.3935 | 2.32E-05 | 0.1344 | 0.0683 |
| rs1706923 | chr7 | 105221097 | RINT1 | -4.3934 | 2.32E-05 | 0.1653 | 0.0683 |
| rs4647576 | chr11 | 65107454 | DPF2 | -4.3931 | 2.32E-05 | 0.1246 | 0.0683 |
| rs2847150 | chr18 | 668300 | ENOSF1 | 4.3928 | 2.32E-05 | 0.1402 | 0.0683 |
| rs4824414 | chr23 | 46642197 | RBM10 | -4.3922 | 2.33E-05 | 0.1242 | 0.0684 |
| rs267770 | chr5 | 36064673 | SKP2 | -4.3921 | 2.33E-05 | 0.1317 | 0.0684 |
| rs2939678 | chr8 | 56247138 | TGS1 | -4.3919 | 2.33E-05 | 0.1254 | 0.0685 |
| rs3756982 | chr6 | 52670444 | GSTA5 | -4.3914 | 2.34E-05 | 0.1337 | 0.0685 |
| rs7955510 | chr12 | 96175195 | NTN4 | -4.3912 | 2.34E-05 | 0.1252 | 0.0686 |
| rs8102985 | chr19 | 51258315 | TSKS | 4.3906 | 2.34E-05 | 0.1263 | 0.0687 |
| rs4541106 | chr17 | 37072192 | STARD3 | 4.3885 | 2.36E-05 | 0.1280 | 0.0692 |
| rs964426 | chr11 | 10632252 | MRVI1 | -4.3855 | 2.39E-05 | 0.1273 | 0.0700 |
| rs16857479 | chr4 | 44770720 | GUF1 | 4.3841 | 2.41E-05 | 0.1259 | 0.0704 |
| rs795971 | chr4 | 140776128 | ELMOD2 | -4.3837 | 2.41E-05 | 0.1436 | 0.0704 |
| rs931593 | chr23 | 78958688 | TBX22 | 4.3834 | 2.41E-05 | 0.1322 | 0.0705 |
| rs17553846 | chr13 | 43613458 | DNAJC15 | -4.3831 | 2.41E-05 | 0.1305 | 0.0705 |
| rs5742926 | chr2 | 190648805 | ORMDL1 | 4.3831 | 2.41E-05 | 0.1350 | 0.0705 |
| rs7791386 | chr7 | 42599161 | HECW1 | -4.3830 | 2.42E-05 | 0.1315 | 0.0705 |
| rs7217091 | chr17 | 17060805 | DRG2 | 4.3824 | 2.42E-05 | 0.1264 | 0.0706 |
| rs3926833 | chr6 | 155576445 | TFB1M | 4.3824 | 2.42E-05 | 0.1267 | 0.0706 |
| rs9929475 | chr16 | 4540885 | CORO7 | -4.3816 | 2.43E-05 | 0.1340 | 0.0707 |
| rs9896735 | chr17 | 5325808 | RABEP1 | -4.3815 | 2.43E-05 | 0.1429 | 0.0707 |
| rs10883559 | chr10 | 102667278 | MRPL43 | 4.3802 | 2.44E-05 | 0.1269 | 0.0711 |
| rs16898280 | chr8 | 101368080 | SPAG1 | -4.3794 | 2.45E-05 | 0.1333 | 0.0712 |
| rs2744267 | chr6 | 25276266 | HIST1H2BA | 4.3790 | 2.45E-05 | 0.1839 | 0.0713 |
| rs573540 | chr11 | 96024669 | CCDC82 | -4.3783 | 2.46E-05 | 0.1256 | 0.0714 |
| rs11645214 | chr16 | 70608987 | COG4 | -4.3783 | 2.46E-05 | 0.1254 | 0.0714 |
| rs7893317 | chr10 | 116419321 | ADRB1 | 4.3782 | 2.46E-05 | 0.1298 | 0.0714 |
| rs6462445 | chr7 | 33053099 | FKBP9 | 4.3779 | 2.47E-05 | 0.1234 | 0.0715 |
| rs9852954 | chr3 | 132911592 | SLCO2A1 | 4.3770 | 2.47E-05 | 0.1268 | 0.0716 |
| rs2126602 | chr15 | 43301476 | PLA2G4E | -4.3770 | 2.47E-05 | 0.1287 | 0.0716 |
| rs10901275 | chr9 | 133599031 | GPR107 | 4.3764 | 2.48E-05 | 0.1536 | 0.0718 |
| rs10146482 | chr14 | 75574087 | MLH3 | -4.3759 | 2.49E-05 | 0.1432 | 0.0719 |
| rs2749010 | chr6 | 52626572 | GSTA5 | -4.3755 | 2.49E-05 | 0.1247 | 0.0719 |
| rs4980968 | chr12 | 883178 | RAD52 | -4.3751 | 2.49E-05 | 0.1253 | 0.0720 |
| rs10891537 | chr11 | 113211370 | TTC12 | 4.3750 | 2.49E-05 | 0.1413 | 0.0720 |
| rs6489746 | chr12 | 865970 | RAD52 | -4.3750 | 2.49E-05 | 0.1265 | 0.0720 |
| rs376206 | chr20 | 44377039 | WFDC10B | 4.3747 | 2.50E-05 | 0.1719 | 0.0720 |
| rs2746100 | chr20 | 34686075 | CPNE1 | -4.3739 | 2.50E-05 | 0.1458 | 0.0722 |
| rs12601491 | chr17 | 4375102 | MYBBP1A | 4.3739 | 2.51E-05 | 0.1302 | 0.0722 |
| rs11127353 | chr2 | 2479822 | PXDN | 4.3734 | 2.51E-05 | 0.1232 | 0.0723 |
| rs16867551 | chr5 | 88634962 | MEF2C | 4.3729 | 2.51E-05 | 0.1270 | 0.0724 |
| rs9911122 | chr17 | 74208424 | FOXJ1 | 4.3725 | 2.52E-05 | 0.1256 | 0.0724 |
| rs501799 | chr11 | 68631240 | MRPL21 | -4.3725 | 2.52E-05 | 0.1274 | 0.0724 |
| rs3088241 | chr11 | 125763746 | PUS3 | 4.3711 | 2.53E-05 | 0.1231 | 0.0727 |
| rs2440467 | chr16 | 56417047 | OGFOD1 | 4.3709 | 2.54E-05 | 0.1235 | 0.0728 |
| rs7940105 | chr11 | 68732026 | MRPL21 | -4.3703 | 2.54E-05 | 0.1230 | 0.0729 |
| rs12973168 | chr19 | 16013338 | CYP4F11 | 4.3702 | 2.54E-05 | 0.1281 | 0.0729 |
| rs11246288 | chr11 | 718436 | PTDSS2 | -4.3696 | 2.55E-05 | 0.1261 | 0.0730 |
| rs4790594 | chr17 | 4137919 | ATP2A3 | 4.3696 | 2.55E-05 | 0.1431 | 0.0730 |
| rs11159121 | chr14 | 75666029 | MLH3 | -4.3689 | 2.55E-05 | 0.1367 | 0.0731 |
| rs11718493 | chr3 | 126247848 | CHST13 | -4.3676 | 2.57E-05 | 0.1236 | 0.0735 |
| rs7223476 | chr17 | 20215352 | AKAP10 | -4.3668 | 2.58E-05 | 0.1600 | 0.0737 |
| rs11466749 | chr5 | 110412585 | STARD4 | -4.3664 | 2.58E-05 | 0.1251 | 0.0737 |
| rs1284108 | chr11 | 93498833 | C11orf54 | -4.3659 | 2.59E-05 | 0.1236 | 0.0739 |
| rs7518839 | chr1 | 224331779 | FBXO28 | -4.3655 | 2.59E-05 | 0.1333 | 0.0739 |
| rs2387715 | chr5 | 180361266 | BTNL8 | -4.3650 | 2.60E-05 | 0.1271 | 0.0740 |
| rs17024195 | chr1 | 110143755 | GNAI3 | -4.3650 | 2.60E-05 | 0.1381 | 0.0740 |
| rs4808394 | chr19 | 15976196 | CYP4F11 | 4.3650 | 2.60E-05 | 0.1235 | 0.0740 |
| rs4338000 | chr7 | 33093389 | FKBP9 | -4.3648 | 2.60E-05 | 0.1281 | 0.0740 |
| rs7308827 | chr12 | 96382644 | AMDHD1 | -4.3646 | 2.60E-05 | 0.1227 | 0.0740 |
| rs2024596 | chr20 | 47518052 | ARFGEF2 | -4.3635 | 2.61E-05 | 0.1329 | 0.0743 |
| rs12147118 | chr14 | 81652975 | GTF2A1 | 4.3626 | 2.62E-05 | 0.1251 | 0.0745 |
| rs223334 | chr4 | 103788211 | MANBA | 4.3624 | 2.62E-05 | 0.1271 | 0.0745 |
| rs4715344 | chr6 | 52697576 | GSTA3 | -4.3623 | 2.62E-05 | 0.1246 | 0.0745 |
| rs224419 | chr20 | 34143092 | CPNE1 | 4.3618 | 2.63E-05 | 0.1557 | 0.0746 |
| rs2765023 | chr1 | 1297065 | B3GALT6 | -4.3618 | 2.63E-05 | 0.1290 | 0.0746 |
| rs799482 | chr14 | 35458745 | NFKBIA | 4.3617 | 2.63E-05 | 0.1427 | 0.0746 |
| rs4534498 | chr10 | 133960422 | DPYSL4 | 4.3611 | 2.64E-05 | 0.1261 | 0.0747 |
| rs16950739 | chr18 | 47138509 | CXXC1 | 4.3600 | 2.65E-05 | 0.1266 | 0.0750 |
| rs2037075 | chr17 | 41305826 | NBR2 | -4.3595 | 2.65E-05 | 0.1298 | 0.0750 |
| rs3848663 | chr19 | 36225620 | U2AF1L4 | -4.3594 | 2.65E-05 | 0.1255 | 0.0750 |
| rs11465670 | chr2 | 103034440 | IL18RAP | 4.3593 | 2.65E-05 | 0.1290 | 0.0750 |
| rs10800409 | chr1 | 162260715 | NOS1AP | 4.3585 | 2.66E-05 | 0.1414 | 0.0752 |
| rs1485587 | chr5 | 81327042 | RPS23 | -4.3580 | 2.67E-05 | 0.1261 | 0.0753 |
| rs4573657 | chr11 | 34434898 | ABTB2 | -4.3580 | 2.67E-05 | 0.1259 | 0.0753 |
| rs920749 | chr17 | 1007540 | YWHAE | -4.3579 | 2.67E-05 | 0.1258 | 0.0753 |
| rs9287718 | chr2 | 10701629 | NOL10 | -4.3579 | 2.67E-05 | 0.1228 | 0.0753 |
| rs6590229 | chr11 | 126655921 | PUS3 | 4.3574 | 2.67E-05 | 0.1224 | 0.0754 |
| rs12378199 | chr9 | 124753832 | NDUFA8 | 4.3570 | 2.68E-05 | 0.1288 | 0.0755 |
| rs4933414 | chr10 | 88580682 | BMPR1A | -4.3568 | 2.68E-05 | 0.1225 | 0.0755 |
| rs9289471 | chr3 | 134230811 | ANAPC13 | -4.3563 | 2.69E-05 | 0.1273 | 0.0755 |
| rs2438127 | chr15 | 61408492 | ANXA2 | 4.3563 | 2.69E-05 | 0.1311 | 0.0755 |
| rs6548222 | chr2 | 296812 | SH3YL1 | 4.3556 | 2.69E-05 | 0.1266 | 0.0757 |
| rs11735407 | chr4 | 130233602 | C4orf33 | 4.3533 | 2.72E-05 | 0.1239 | 0.0763 |
| rs4661594 | chr1 | 15507952 | TMEM51 | 4.3532 | 2.72E-05 | 0.1732 | 0.0763 |
| rs13284731 | chr9 | 95880252 | NINJ1 | -4.3529 | 2.72E-05 | 0.1375 | 0.0764 |
| rs2359964 | chr2 | 207949082 | NDUFS1 | 4.3526 | 2.73E-05 | 0.1302 | 0.0764 |
| rs5764698 | chr22 | 45749983 | RIBC2 | 4.3524 | 2.73E-05 | 0.1275 | 0.0765 |
| rs2067040 | chr16 | 66764809 | C16orf70 | 4.3518 | 2.74E-05 | 0.1258 | 0.0766 |
| rs2187986 | chr11 | 115001410 | CADM1 | -4.3508 | 2.75E-05 | 0.1452 | 0.0769 |
| rs12274772 | chr11 | 115098768 | CADM1 | -4.3505 | 2.75E-05 | 0.1400 | 0.0769 |
| rs2276360 | chr11 | 71169547 | NADSYN1 | 4.3502 | 2.75E-05 | 0.1429 | 0.0769 |
| rs150893 | chr4 | 103738232 | MANBA | 4.3501 | 2.75E-05 | 0.1231 | 0.0769 |
| rs5743047 | chr2 | 190684668 | ORMDL1 | 4.3500 | 2.75E-05 | 0.1224 | 0.0769 |
| rs7961807 | chr12 | 96356807 | AMDHD1 | 4.3499 | 2.76E-05 | 0.1225 | 0.0769 |
| rs1409158 | chr1 | 119538890 | WARS2 | 4.3494 | 2.76E-05 | 0.1303 | 0.0770 |
| rs11874836 | chr18 | 47360028 | DYM | -4.3492 | 2.76E-05 | 0.9797 | 0.0771 |
| rs6136499 | chr20 | 18717724 | DTD1 | -4.3485 | 2.77E-05 | 0.1279 | 0.0772 |
| rs17103412 | chr14 | 66725775 | FUT8 | 4.3478 | 2.78E-05 | 0.1219 | 0.0774 |
| rs2516739 | chr16 | 2097158 | SLC9A3R2 | -4.3478 | 2.78E-05 | 0.1218 | 0.0774 |
| rs961067 | chr4 | 2505606 | RNF4 | 4.3454 | 2.81E-05 | 0.1251 | 0.0781 |
| rs658181 | chr11 | 93506034 | C11orf54 | -4.3446 | 2.81E-05 | 0.1253 | 0.0782 |
| rs6582294 | chr12 | 76034992 | GLIPR1L2 | -4.3445 | 2.82E-05 | 0.1265 | 0.0782 |
| rs2285934 | chr12 | 113351520 | OAS1 | -4.3444 | 2.82E-05 | 0.1258 | 0.0782 |
| rs185397 | chr16 | 58762311 | KATNB1 | -4.3443 | 2.82E-05 | 0.1217 | 0.0782 |
| rs2282580 | chr11 | 126081403 | TIRAP | -4.3442 | 2.82E-05 | 0.1217 | 0.0782 |
| rs850622 | chr17 | 19837536 | AKAP10 | 4.3437 | 2.82E-05 | 0.1439 | 0.0783 |
| rs12528822 | chr6 | 109661541 | CD164 | 4.3423 | 2.84E-05 | 0.1229 | 0.0787 |
| rs12950642 | chr17 | 74206340 | FOXJ1 | 4.3418 | 2.85E-05 | 0.1289 | 0.0788 |
| rs9911983 | chr17 | 45885756 | MRPL10 | -4.3416 | 2.85E-05 | 0.1309 | 0.0788 |
| rs4864984 | chr4 | 56240287 | TMEM165 | 4.3409 | 2.86E-05 | 0.1381 | 0.0790 |
| rs1777668 | chr13 | 36153301 | NBEA | 4.3387 | 2.88E-05 | 0.1500 | 0.0797 |
| rs1859708 | chr2 | 75862484 | MRPL19 | -4.3386 | 2.88E-05 | 0.1214 | 0.0797 |
| rs10891545 | chr11 | 113258065 | TTC12 | 4.3382 | 2.89E-05 | 0.1221 | 0.0797 |
| rs1568585 | chr3 | 150332011 | EIF2A | -4.3378 | 2.89E-05 | 0.1239 | 0.0798 |
| rs17544039 | chr17 | 42693848 | SOST | 4.3378 | 2.89E-05 | 0.1257 | 0.0798 |
| rs1858446 | chr10 | 5852172 | GDI2 | -4.3375 | 2.90E-05 | 0.1213 | 0.0798 |
| rs2404843 | chr1 | 224299719 | FBXO28 | -4.3363 | 2.91E-05 | 0.1405 | 0.0801 |
| rs9446955 | chr6 | 74339192 | SLC17A5 | 4.3362 | 2.91E-05 | 0.1223 | 0.0801 |
| rs6811616 | chr4 | 57194712 | PPAT | -4.3360 | 2.91E-05 | 0.1213 | 0.0802 |
| rs787240 | chr9 | 135560139 | GTF3C4 | -4.3354 | 2.92E-05 | 0.1561 | 0.0803 |
| rs7792037 | chr7 | 19291993 | TWIST1 | -4.3350 | 2.92E-05 | 0.1212 | 0.0803 |
| rs12081842 | chr1 | 34383108 | TRIM62 | -4.3349 | 2.92E-05 | 0.1211 | 0.0803 |
| rs175435 | chr14 | 75612025 | EIF2B2 | -4.3349 | 2.92E-05 | 0.1614 | 0.0803 |
| rs17340542 | chr7 | 128720045 | IRF5 | 4.3347 | 2.93E-05 | 0.1227 | 0.0804 |
| rs8043812 | chr16 | 23662796 | DCTN5 | -4.3345 | 2.93E-05 | 0.1261 | 0.0804 |
| rs478647 | chr11 | 68700424 | MRPL21 | -4.3344 | 2.93E-05 | 0.1212 | 0.0804 |
| rs6519120 | chr22 | 38991393 | GTPBP1 | -4.3339 | 2.94E-05 | 0.1215 | 0.0805 |
| rs6842840 | chr4 | 100887972 | DNAJB14 | -4.3338 | 2.94E-05 | 0.1286 | 0.0805 |
| rs7079565 | chr10 | 16590829 | PTER | -4.3336 | 2.94E-05 | 0.1229 | 0.0805 |
| rs17140993 | chr7 | 19288379 | TWIST1 | -4.3325 | 2.95E-05 | 0.1212 | 0.0808 |
| rs2583896 | chr7 | 42751965 | HECW1 | -4.3325 | 2.95E-05 | 0.1220 | 0.0808 |
| rs2966205 | chr16 | 56446612 | OGFOD1 | 4.3307 | 2.97E-05 | 0.1223 | 0.0813 |
| rs11932 | chr3 | 122141135 | CCDC58 | -4.3305 | 2.98E-05 | 0.1217 | 0.0813 |
| rs1194758 | chr11 | 65238085 | DPF2 | -4.3303 | 2.98E-05 | 0.1419 | 0.0813 |
| rs10951192 | chr7 | 28161033 | HOXA13 | -4.3301 | 2.98E-05 | 0.1225 | 0.0813 |
| rs211585 | chr3 | 139235564 | RBP1 | 4.3297 | 2.99E-05 | 0.1223 | 0.0814 |
| rs4793229 | chr17 | 41418334 | NBR2 | -4.3296 | 2.99E-05 | 0.1231 | 0.0814 |
| rs6060370 | chr20 | 33909752 | CPNE1 | 4.3295 | 2.99E-05 | 0.1327 | 0.0814 |
| rs2277693 | chr17 | 45909183 | MRPL10 | 4.3292 | 2.99E-05 | 0.1228 | 0.0815 |
| rs2587871 | chr16 | 56412526 | OGFOD1 | 4.3287 | 3.00E-05 | 0.1209 | 0.0815 |
| rs2587873 | chr16 | 56412734 | OGFOD1 | 4.3287 | 3.00E-05 | 0.1209 | 0.0815 |
| rs6499837 | chr16 | 56453981 | OGFOD1 | 4.3287 | 3.00E-05 | 0.1209 | 0.0815 |
| rs17458304 | chr5 | 113804486 | PGGT1B | -4.3285 | 3.00E-05 | 0.1230 | 0.0815 |
| rs7307025 | chr12 | 53948786 | KRT72 | -4.3281 | 3.00E-05 | 0.1257 | 0.0816 |
| rs5927704 | chr23 | 31225071 | MAGEB3 | -4.3273 | 3.01E-05 | 0.1285 | 0.0818 |
| rs9725887 | chr1 | 179042227 | FAM20B | 4.3265 | 3.02E-05 | 0.1236 | 0.0820 |
| rs721575 | chr17 | 61782920 | FTSJ3 | 4.3262 | 3.03E-05 | 0.1298 | 0.0821 |
| rs7651886 | chr3 | 11572728 | ATG7 | 4.3261 | 3.03E-05 | 0.1226 | 0.0821 |
| rs11609603 | chr12 | 95948366 | NR2C1 | 4.3254 | 3.04E-05 | 0.1232 | 0.0823 |
| rs10517118 | chr4 | 44760236 | GUF1 | 4.3249 | 3.04E-05 | 0.1222 | 0.0823 |
| rs13229290 | chr7 | 54779952 | SEC61G | -4.3248 | 3.04E-05 | 0.1215 | 0.0823 |
| rs2305169 | chr11 | 36298101 | RAG1 | 4.3247 | 3.05E-05 | 0.1230 | 0.0823 |
| rs10085826 | chr7 | 42504691 | HECW1 | -4.3243 | 3.05E-05 | 0.1206 | 0.0824 |
| rs11083046 | chr18 | 51781019 | POLI | 4.3225 | 3.07E-05 | 0.1311 | 0.0830 |
| rs17138855 | chr10 | 16649790 | PTER | 4.3221 | 3.08E-05 | 0.1205 | 0.0831 |
| rs2500432 | chr20 | 25290512 | ABHD12 | 4.3216 | 3.08E-05 | 0.1697 | 0.0832 |
| rs7412396 | chr1 | 150666797 | HORMAD1 | 4.3215 | 3.08E-05 | 0.1321 | 0.0832 |
| rs7209653 | chr17 | 19882084 | AKAP10 | 4.3211 | 3.09E-05 | 0.1379 | 0.0833 |
| rs12225960 | chr11 | 115043055 | CADM1 | -4.3201 | 3.10E-05 | 0.1477 | 0.0835 |
| rs1006147 | chr1 | 16046109 | SLC25A34 | -4.3199 | 3.10E-05 | 0.1274 | 0.0836 |
| rs875500 | chr11 | 125698654 | PUS3 | 4.3192 | 3.11E-05 | 0.1205 | 0.0837 |
| rs4149128 | chr12 | 21028774 | ABCC9 | 4.3190 | 3.11E-05 | 0.1745 | 0.0837 |
| rs12332040 | chr4 | 146829530 | SLC10A7 | 4.3190 | 3.12E-05 | 0.1204 | 0.0837 |
| rs935614 | chr2 | 135932720 | CCNT2 | -4.3181 | 3.13E-05 | 0.1203 | 0.0840 |
| rs10958708 | chr8 | 42003108 | HOOK3 | -4.3179 | 3.13E-05 | 0.1215 | 0.0840 |
| rs6088466 | chr20 | 32913534 | TRPC4AP | 4.3177 | 3.13E-05 | 0.1254 | 0.0840 |
| rs800861 | chr2 | 128474326 | SAP130 | 4.3176 | 3.13E-05 | 0.1250 | 0.0840 |
| rs16857329 | chr4 | 44662171 | GUF1 | 4.3162 | 3.15E-05 | 0.1293 | 0.0844 |
| rs1450050 | chr3 | 132911040 | SLCO2A1 | 4.3161 | 3.15E-05 | 0.1267 | 0.0844 |
| rs12615616 | chr2 | 20855911 | APOB | -4.3154 | 3.16E-05 | 0.1304 | 0.0846 |
| rs8080662 | chr17 | 46190819 | SNX11 | -4.3154 | 3.16E-05 | 0.1365 | 0.0846 |
| rs10798651 | chr1 | 179048423 | TOR3A | -4.3152 | 3.16E-05 | 0.1304 | 0.0846 |
| rs11601041 | chr11 | 115083348 | CADM1 | -4.3149 | 3.17E-05 | 0.1275 | 0.0847 |
| rs2713651 | chr3 | 124272171 | ITGB5 | -4.3131 | 3.19E-05 | 0.1201 | 0.0852 |
| rs7156586 | chr14 | 75506230 | EIF2B2 | -4.3128 | 3.19E-05 | 0.1547 | 0.0852 |
| rs763151 | chr16 | 449345 | DECR2 | 4.3112 | 3.21E-05 | 0.1252 | 0.0857 |
| rs11807 | chr1 | 110260742 | GSTM1 | 4.3112 | 3.21E-05 | 0.1303 | 0.0857 |
| rs1466835 | chr3 | 12579944 | RAF1 | -4.3111 | 3.21E-05 | 0.1237 | 0.0857 |
| rs2145082 | chr20 | 33905326 | CPNE1 | 4.3108 | 3.22E-05 | 0.1593 | 0.0858 |
| rs2324142 | chr17 | 16041306 | ZSWIM7 | 4.3103 | 3.22E-05 | 0.1405 | 0.0859 |
| rs3820163 | chr1 | 179041011 | FAM20B | 4.3101 | 3.23E-05 | 0.1216 | 0.0859 |
| rs748193 | chr8 | 145682115 | LRRC14 | -4.3098 | 3.23E-05 | 0.1326 | 0.0859 |
| rs12449580 | chr17 | 6337247 | ALOX12 | 4.3093 | 3.24E-05 | 0.1311 | 0.0861 |
| rs6961098 | chr7 | 25140047 | CYCS | 4.3091 | 3.24E-05 | 0.1286 | 0.0861 |
| rs12151768 | chr2 | 70497824 | FAM136A | -4.3091 | 3.24E-05 | 0.1219 | 0.0861 |
| rs8060794 | chr16 | 88368499 | ZFPM1 | -4.3088 | 3.24E-05 | 0.1594 | 0.0861 |
| rs2704767 | chr12 | 75903191 | GLIPR1L2 | -4.3085 | 3.25E-05 | 0.1198 | 0.0862 |
| rs763121 | chr22 | 38879940 | CBY1 | 4.3081 | 3.25E-05 | 0.1812 | 0.0862 |
| rs2573276 | chr13 | 46493812 | HTR2A | 4.3081 | 3.25E-05 | 0.1260 | 0.0862 |
| rs12646793 | chr4 | 150886317 | LRBA | 4.3075 | 3.26E-05 | 0.1221 | 0.0864 |
| rs2709370 | chr2 | 208382602 | CREB1 | 4.3062 | 3.28E-05 | 0.1197 | 0.0868 |
| rs11950217 | chr5 | 81189826 | RPS23 | -4.3060 | 3.28E-05 | 0.1229 | 0.0868 |
| rs1048687 | chr16 | 9212987 | C16orf72 | 4.3057 | 3.28E-05 | 0.1313 | 0.0869 |
| rs9687397 | chr5 | 34575747 | PRLR | -4.3054 | 3.29E-05 | 0.1246 | 0.0869 |
| rs12230722 | chr12 | 95956778 | NR2C1 | -4.3045 | 3.30E-05 | 0.1200 | 0.0872 |
| rs1051527 | chr18 | 668376 | ENOSF1 | 4.3043 | 3.30E-05 | 0.1322 | 0.0872 |
| rs10066278 | chr5 | 140156318 | ZMAT2 | -4.3042 | 3.30E-05 | 0.1235 | 0.0872 |
| rs7040878 | chr9 | 85765521 | RASEF | 4.3030 | 3.32E-05 | 0.1284 | 0.0876 |
| rs1482337 | chr8 | 22395509 | PPP3CC | -4.3023 | 3.33E-05 | 0.1449 | 0.0877 |
| rs3815575 | chr12 | 109947905 | UBE3B | 4.3022 | 3.33E-05 | 0.1195 | 0.0878 |
| rs12219858 | chr10 | 102685584 | MRPL43 | 4.3019 | 3.33E-05 | 0.1282 | 0.0878 |
| rs16981436 | chr20 | 56473826 | C20orf85 | 4.3019 | 3.33E-05 | 0.1353 | 0.0878 |
| rs4652895 | chr1 | 36316571 | C1orf216 | -4.3016 | 3.34E-05 | 0.1211 | 0.0878 |
| rs1535708 | chr6 | 6520931 | RIOK1 | 4.3011 | 3.34E-05 | 0.1236 | 0.0879 |
| rs318427 | chr6 | 2945331 | NQO2 | -4.3009 | 3.34E-05 | 0.1194 | 0.0879 |
| rs7734902 | chr5 | 80619470 | RASGRF2 | 4.3005 | 3.35E-05 | 0.1370 | 0.0880 |
| rs6886255 | chr5 | 176718361 | PRELID1 | -4.2994 | 3.37E-05 | 0.1222 | 0.0884 |
| rs1442987 | chr18 | 28038282 | DSC3 | -4.2990 | 3.37E-05 | 0.1376 | 0.0884 |
| rs6698826 | chr1 | 52374952 | RAB3B | 4.2990 | 3.37E-05 | 0.1195 | 0.0884 |
| rs9395826 | chr6 | 52679690 | GSTA3 | -4.2986 | 3.38E-05 | 0.1394 | 0.0885 |
| rs2297657 | chr1 | 53746595 | C1orf123 | -4.2983 | 3.38E-05 | 0.1213 | 0.0886 |
| rs1891222 | chr1 | 119751002 | WARS2 | 4.2976 | 3.39E-05 | 0.1258 | 0.0888 |
| rs4906269 | chr14 | 103362017 | MARK3 | 4.2972 | 3.39E-05 | 0.1207 | 0.0889 |
| rs2982619 | chr11 | 34150839 | FBXO3 | 4.2971 | 3.40E-05 | 0.1228 | 0.0889 |
| rs10744601 | chr12 | 3405902 | CCND2 | 4.2970 | 3.40E-05 | 0.1213 | 0.0889 |
| rs1535225 | chr20 | 4157853 | PANK2 | -4.2966 | 3.40E-05 | 0.1272 | 0.0889 |
| rs5906184 | chr23 | 46209518 | RGN | 4.2964 | 3.40E-05 | 0.1415 | 0.0890 |
| rs4425985 | chr1 | 116207833 | ATP1A1 | -4.2962 | 3.41E-05 | 0.1314 | 0.0890 |
| rs17183465 | chr12 | 110029750 | UBE3B | -4.2960 | 3.41E-05 | 0.1234 | 0.0890 |
| rs1719236 | chr15 | 45577783 | SLC28A2 | -4.2956 | 3.42E-05 | 0.1464 | 0.0891 |
| rs9371369 | chr6 | 155579557 | TFB1M | 4.2954 | 3.42E-05 | 0.1214 | 0.0891 |
| rs11611277 | chr12 | 109826634 | UBE3B | -4.2951 | 3.42E-05 | 0.1512 | 0.0892 |
| rs2791961 | chr1 | 36371419 | C1orf216 | -4.2948 | 3.43E-05 | 0.1191 | 0.0893 |
| rs507900 | chr10 | 131398144 | MGMT | 4.2947 | 3.43E-05 | 0.1254 | 0.0893 |
| rs10417536 | chr19 | 18364800 | PDE4C | 4.2944 | 3.43E-05 | 0.1191 | 0.0893 |
| rs218489 | chr3 | 130693691 | NEK11 | 4.2939 | 3.44E-05 | 0.1225 | 0.0894 |
| rs3742604 | chr14 | 65026242 | ZBTB1 | 4.2936 | 3.44E-05 | 0.1339 | 0.0895 |
| rs934141 | chr15 | 84224793 | SH3GL3 | -4.2934 | 3.45E-05 | 0.1204 | 0.0895 |
| rs2002878 | chr2 | 69738093 | GFPT1 | -4.2920 | 3.46E-05 | 0.1222 | 0.0900 |
| rs7290139 | chr22 | 45718743 | RIBC2 | 4.2916 | 3.47E-05 | 0.1216 | 0.0901 |
| rs2850900 | chr18 | 75015847 | GALR1 | 4.2915 | 3.47E-05 | 0.1463 | 0.0901 |
| rs252895 | chr5 | 56224124 | MIER3 | -4.2912 | 3.48E-05 | 0.1193 | 0.0901 |
| rs6799694 | chr3 | 196264713 | TFRC | 4.2911 | 3.48E-05 | 0.1338 | 0.0901 |
| rs1148007 | chr12 | 75972957 | GLIPR1L2 | -4.2901 | 3.49E-05 | 0.1354 | 0.0904 |
| rs1967381 | chr10 | 14071075 | OPTN | -4.2899 | 3.49E-05 | 0.1194 | 0.0904 |
| rs994423 | chr23 | 151382195 | MAGEA5 | 4.2896 | 3.50E-05 | 0.1200 | 0.0905 |
| rs12610082 | chr19 | 5227008 | C19orf10 | 4.2886 | 3.51E-05 | 0.1188 | 0.0908 |
| rs1145077 | chr15 | 45683795 | SLC28A2 | -4.2880 | 3.52E-05 | 0.1427 | 0.0910 |
| rs1298964 | chr3 | 102299605 | ZBTB11 | 4.2879 | 3.52E-05 | 0.1436 | 0.0910 |
| rs6669370 | chr1 | 114506675 | DCLRE1B | 4.2871 | 3.53E-05 | 0.1293 | 0.0912 |
| rs4661003 | chr1 | 40290696 | PABPC4 | -4.2867 | 3.54E-05 | 0.1188 | 0.0913 |
| rs223452 | chr4 | 103711434 | MANBA | 4.2865 | 3.54E-05 | 0.1191 | 0.0913 |
| rs7605014 | chr2 | 75905719 | MRPL19 | -4.2859 | 3.55E-05 | 0.1201 | 0.0914 |
| rs11108331 | chr12 | 96309290 | NTN4 | -4.2858 | 3.55E-05 | 0.1203 | 0.0914 |
| rs1745901 | chr10 | 88768657 | BMPR1A | 4.2858 | 3.55E-05 | 0.1187 | 0.0914 |
| rs7313235 | chr12 | 10132283 | CLEC12A | 4.2857 | 3.55E-05 | 0.1215 | 0.0914 |
| rs6429661 | chr1 | 14780811 | TMEM51 | -4.2854 | 3.56E-05 | 0.1197 | 0.0915 |
| rs4975016 | chr4 | 39439719 | FAM114A1 | 4.2849 | 3.56E-05 | 0.1227 | 0.0916 |
| rs9944085 | chr14 | 81717620 | GTF2A1 | 4.2847 | 3.57E-05 | 0.1327 | 0.0916 |
| rs6050425 | chr20 | 25178119 | ABHD12 | 4.2845 | 3.57E-05 | 0.1234 | 0.0917 |
| rs8176126 | chr17 | 41259049 | NBR2 | -4.2831 | 3.59E-05 | 0.1195 | 0.0921 |
| rs864344 | chr12 | 53121995 | KRT1 | 4.2830 | 3.59E-05 | 0.1185 | 0.0921 |
| rs12792346 | chr11 | 96175277 | CCDC82 | 4.2826 | 3.60E-05 | 0.1189 | 0.0922 |
| rs1163254 | chr12 | 81104350 | PPFIA2 | -4.2822 | 3.60E-05 | 0.1256 | 0.0923 |
| rs1864183 | chr5 | 81549216 | RPS23 | 4.2817 | 3.61E-05 | 0.1203 | 0.0924 |
| rs4693555 | chr4 | 83992535 | THAP9 | -4.2815 | 3.61E-05 | 0.1211 | 0.0925 |
| rs948025 | chr11 | 120679052 | GRIK4 | 4.2806 | 3.62E-05 | 0.1184 | 0.0927 |
| rs11623413 | chr14 | 75588824 | EIF2B2 | -4.2797 | 3.64E-05 | 0.1261 | 0.0930 |
| rs163260 | chr16 | 20666782 | ACSM1 | 4.2794 | 3.64E-05 | 0.1191 | 0.0931 |
| rs17035 | chr17 | 45900968 | MRPL10 | 4.2790 | 3.65E-05 | 0.1217 | 0.0932 |
| rs35635 | chr16 | 23693770 | DCTN5 | -4.2788 | 3.65E-05 | 0.1193 | 0.0932 |
| rs7734188 | chr5 | 81647580 | RPS23 | 4.2787 | 3.65E-05 | 0.1214 | 0.0932 |
| rs869987 | chr1 | 53723190 | C1orf123 | -4.2784 | 3.66E-05 | 0.1191 | 0.0933 |
| rs7110115 | chr11 | 43780193 | HSD17B12 | -4.2770 | 3.67E-05 | 0.1210 | 0.0937 |
| rs9911630 | chr17 | 41188342 | NBR2 | -4.2764 | 3.68E-05 | 0.1383 | 0.0939 |
| rs4729644 | chr7 | 100669857 | VGF | -4.2762 | 3.69E-05 | 0.1248 | 0.0939 |
| rs9913360 | chr17 | 2725145 | MNT | -4.2760 | 3.69E-05 | 0.1201 | 0.0939 |
| rs2814167 | chr10 | 133989118 | DPYSL4 | 4.2759 | 3.69E-05 | 0.1184 | 0.0939 |
| rs713354 | chr3 | 140718373 | CLSTN2 | 4.2757 | 3.69E-05 | 0.1181 | 0.0940 |
| rs1359182 | chr13 | 43603093 | DNAJC15 | -4.2756 | 3.70E-05 | 0.1371 | 0.0940 |
| rs12503191 | chr4 | 15935497 | FGFBP2 | -4.2741 | 3.72E-05 | 0.1397 | 0.0945 |
| rs10838183 | chr11 | 43855072 | HSD17B12 | -4.2740 | 3.72E-05 | 0.1186 | 0.0945 |
| rs1736657 | chr23 | 78579355 | TBX22 | 4.2738 | 3.72E-05 | 0.1268 | 0.0945 |
| rs2853528 | chr18 | 668650 | ENOSF1 | 4.2731 | 3.73E-05 | 0.1317 | 0.0947 |
| rs17084735 | chr13 | 27411121 | RNF6 | -4.2721 | 3.75E-05 | 0.1191 | 0.0950 |
| rs996906 | chr3 | 86607698 | CHMP2B | -4.2713 | 3.76E-05 | 0.1262 | 0.0952 |
| rs14234 | chr2 | 70524142 | FAM136A | -4.2713 | 3.76E-05 | 0.1191 | 0.0952 |
| rs12038567 | chr1 | 100348655 | SASS6 | -4.2710 | 3.76E-05 | 0.1207 | 0.0953 |
| rs7332994 | chr13 | 48674135 | MED4 | -4.2709 | 3.76E-05 | 0.1302 | 0.0953 |
| rs2207951 | chr6 | 52611118 | GSTA3 | -4.2707 | 3.77E-05 | 0.1205 | 0.0953 |
| rs10793970 | chr9 | 133635546 | GPR107 | 4.2705 | 3.77E-05 | 0.1277 | 0.0953 |
| rs9514064 | chr13 | 103488276 | BIVM | 4.2691 | 3.79E-05 | 0.1324 | 0.0958 |
| rs869638 | chr11 | 118100411 | MPZL3 | 4.2691 | 3.79E-05 | 0.1997 | 0.0958 |
| rs471390 | chr10 | 131382825 | MGMT | 4.2688 | 3.79E-05 | 0.1277 | 0.0958 |
| rs1500530 | chr3 | 122275472 | CCDC58 | 4.2682 | 3.80E-05 | 0.1220 | 0.0960 |
| rs1167636 | chr12 | 26058772 | LRMP | 4.2671 | 3.82E-05 | 0.1177 | 0.0964 |
| rs7263437 | chr20 | 44410887 | WFDC10B | 4.2666 | 3.83E-05 | 0.1254 | 0.0965 |
| rs4431432 | chr6 | 48970405 | CRISP1 | 4.2660 | 3.84E-05 | 0.1180 | 0.0967 |
| rs2301178 | chr10 | 118404700 | PNLIPRP2 | 4.2658 | 3.84E-05 | 0.1207 | 0.0967 |
| rs659495 | chr7 | 129194092 | CPA1 | -4.2648 | 3.86E-05 | 0.1178 | 0.0970 |
| rs958075 | chr20 | 25595123 | ABHD12 | 4.2647 | 3.86E-05 | 0.1584 | 0.0971 |
| rs3809116 | chr12 | 122715535 | SETD1B | -4.2639 | 3.87E-05 | 0.1856 | 0.0973 |
| rs3003930 | chr6 | 39852037 | MOCS1 | -4.2638 | 3.87E-05 | 0.1182 | 0.0973 |
| rs2366988 | chr12 | 75958858 | GLIPR1L2 | -4.2625 | 3.89E-05 | 0.1199 | 0.0977 |
| rs13000817 | chr2 | 70273707 | FAM136A | -4.2620 | 3.90E-05 | 0.1219 | 0.0979 |
| rs6489756 | chr12 | 964506 | RAD52 | -4.2619 | 3.90E-05 | 0.1221 | 0.0979 |
| rs2170387 | chr3 | 98997337 | TBC1D23 | -4.2617 | 3.90E-05 | 0.1516 | 0.0979 |
| rs2637658 | chr10 | 133992803 | DPYSL4 | 4.2615 | 3.91E-05 | 0.1222 | 0.0979 |
| rs1240376 | chr10 | 88733150 | BMPR1A | 4.2612 | 3.91E-05 | 0.1363 | 0.0980 |
| rs7638552 | chr3 | 58091098 | FLNB | -4.2609 | 3.92E-05 | 0.1220 | 0.0981 |
| rs4246533 | chr1 | 157552944 | FCRL3 | -4.2604 | 3.92E-05 | 0.1180 | 0.0982 |
| rs12478277 | chr2 | 178886723 | FKBP7 | -4.2599 | 3.93E-05 | 0.1235 | 0.0982 |
| rs3011894 | chr6 | 155575867 | TFB1M | 4.2598 | 3.93E-05 | 0.1215 | 0.0982 |
| rs4903292 | chr14 | 75656245 | MLH3 | -4.2598 | 3.93E-05 | 0.1265 | 0.0982 |
| rs9303417 | chr17 | 58168322 | HEATR6 | 4.2597 | 3.93E-05 | 0.1173 | 0.0982 |
| rs5928423 | chr23 | 29830159 | MAGEB4 | 4.2596 | 3.93E-05 | 0.1205 | 0.0982 |
| rs1232027 | chr5 | 79915020 | DHFR | -4.2594 | 3.94E-05 | 0.1218 | 0.0983 |
| rs7317685 | chr13 | 48715753 | MED4 | -4.2592 | 3.94E-05 | 0.1296 | 0.0983 |
| rs4451093 | chr5 | 140158542 | ZMAT2 | -4.2589 | 3.94E-05 | 0.1311 | 0.0984 |
| rs2073077 | chr20 | 25195913 | ABHD12 | 4.2587 | 3.95E-05 | 0.1199 | 0.0984 |
| rs6847 | chr17 | 46147807 | SNX11 | -4.2584 | 3.95E-05 | 0.1351 | 0.0985 |
| rs11068715 | chr12 | 118327954 | TAOK3 | -4.2579 | 3.96E-05 | 0.1178 | 0.0986 |
| rs3779647 | chr8 | 30560887 | GSR | -4.2578 | 3.96E-05 | 0.1251 | 0.0986 |
| rs11554583 | chr19 | 1357830 | CIRBP | -4.2577 | 3.96E-05 | 0.1273 | 0.0986 |
| rs7256746 | chr19 | 37665975 | ZNF420 | -4.2574 | 3.97E-05 | 0.1405 | 0.0987 |
| rs11000948 | chr10 | 76031721 | USP54 | -4.2569 | 3.98E-05 | 0.1172 | 0.0988 |
| rs11001014 | chr10 | 76128832 | USP54 | -4.2569 | 3.98E-05 | 0.1172 | 0.0988 |
| rs4766335 | chr12 | 5168849 | FGF6 | 4.2568 | 3.98E-05 | 0.1199 | 0.0988 |
| rs9814342 | chr3 | 196271307 | TFRC | 4.2552 | 4.00E-05 | 0.1177 | 0.0993 |
| rs17346077 | chr4 | 129740590 | C4orf33 | 4.2550 | 4.01E-05 | 0.1171 | 0.0993 |
| rs2563287 | chr5 | 140124786 | ZMAT2 | -4.2543 | 4.02E-05 | 0.1239 | 0.0996 |
| rs491745 | chr11 | 96043720 | CCDC82 | -4.2532 | 4.03E-05 | 0.1169 | 0.1000 |

**Table S3. Cis-eQTL analysis for significant associations between GWAS SNPs and genes expression within ± 1Mb.**

| **risk SNP** | **Region** | **Gene** | ***P value*** | **FDR** |
| --- | --- | --- | --- | --- |
| rs8180040 | chr3 | CCDC12 | 8.83E-07 | 0.0059 |
| rs7226855 | chr18 | DYM | 0.0013 | 0.5162 |
| rs5934683 | chr23 | WWC3 | 0.0043 | 0.6845 |
| rs5005940 | chr20 | MCM8 | 0.0058 | 0.7229 |
| rs3217810 | chr12 | DYRK4 | 0.0092 | 0.7718 |
| rs4925386 | chr20 | C20orf166 | 0.0092 | 0.7721 |
| rs12696304 | chr3 | PRKCI | 0.0098 | 0.7777 |
| rs4813802 | chr20 | C20orf196 | 0.0122 | 0.7966 |
| rs3123636 | chr6 | PARK2 | 0.0134 | 0.8044 |
| rs39454 | chr7 | C7orf31 | 0.0178 | 0.8280 |
| rs827401 | chr10 | KIN | 0.0186 | 0.8313 |
| rs3802840 | chr11 | POU2AF1 | 0.0187 | 0.8319 |
| rs4488237 | chr12 | TSPAN8 | 0.0247 | 0.8526 |
| rs9925923 | chr16 | VPS4A | 0.0269 | 0.8573 |
| rs3104964 | chr8 | CCNE2 | 0.0296 | 0.8648 |
| rs1892124 | chr1 | SH2D1B | 0.0327 | 0.8707 |
| rs12696304 | chr3 | CLDN11 | 0.0361 | 0.8766 |
| rs713424 | chr14 | SOCS4 | 0.0403 | 0.8845 |
| rs12657484 | chr5 | C5orf24 | 0.0428 | 0.8873 |
| rs10899024 | chr11 | MRPL48 | 0.0428 | 0.8873 |
| rs11934535 | chr4 | GRID2 | 0.0447 | 0.8900 |
| rs10797838 | chr1 | GLUL | 0.0456 | 0.8910 |
| rs1892124 | chr1 | HSD17B7 | 0.0461 | 0.8915 |
| rs12657484 | chr5 | H2AFY | 0.0471 | 0.8927 |
| rs1321310 | chr6 | C6orf89 | 0.0482 | 0.8942 |
| rs9925923 | chr16 | TERF2 | 0.0524 | 0.8982 |
| rs5934683 | chr23 | CLCN4 | 0.0553 | 0.9013 |
| rs4925386 | chr20 | PSMA7 | 0.0558 | 0.9017 |
| rs5934683 | chr23 | MID1 | 0.0590 | 0.9052 |
| rs11169544 | chr12 | POU6F1 | 0.0605 | 0.9064 |
| rs10774214 | chr12 | EFCAB4B | 0.0615 | 0.9073 |
| rs3786934 | chr19 | CHST8 | 0.0620 | 0.9076 |
| rs11579490 | chr1 | TAF1A | 0.0649 | 0.9098 |
| rs9925923 | chr16 | EDC4 | 0.0655 | 0.9101 |
| rs4925386 | chr20 | SLCO4A1 | 0.0672 | 0.9114 |
| rs9925923 | chr16 | COG8 | 0.0690 | 0.9127 |
| rs7315438 | chr12 | MED13L | 0.0698 | 0.9129 |
| rs10797838 | chr1 | RGL1 | 0.0735 | 0.9161 |
| rs1892124 | chr1 | NOS1AP | 0.0782 | 0.9191 |
| rs12696304 | chr3 | GPR160 | 0.0785 | 0.9193 |
| rs10774214 | chr12 | FGF6 | 0.0823 | 0.9220 |
| rs10899024 | chr11 | CHRDL2 | 0.0823 | 0.9221 |
| rs17087196 | chr4 | EPHA5 | 0.0841 | 0.9229 |
| rs1665650 | chr10 | EMX2 | 0.0855 | 0.9239 |
| rs59336 | chr12 | RBM19 | 0.0855 | 0.9239 |
| rs9925923 | chr16 | CYB5B | 0.0874 | 0.9249 |
| rs2071047 | chr14 | DDHD1 | 0.0875 | 0.9250 |
| rs11579490 | chr1 | DUSP10 | 0.0890 | 0.9255 |
| rs6911915 | chr6 | VGLL2 | 0.0909 | 0.9266 |
| rs9925923 | chr16 | TMED6 | 0.0922 | 0.9274 |
| rs710005 | chr14 | RTN1 | 0.0930 | 0.9277 |
| rs9925923 | chr16 | NOB1 | 0.0949 | 0.9286 |
| rs7315438 | chr12 | TBX3 | 0.0971 | 0.9296 |
| rs1321310 | chr6 | CLPS | 0.0983 | 0.9299 |

**Table S4. Results and analysis of iTRAQ assay.**

| Accession | GENE/Protein | NC-1 | NC-2 | saCCDC12-1 | saCCDC12-2 | Foldchange | Foldchange CV | *P value* | adj. *P value* | Level |
| --- | --- | --- | --- | --- | --- | --- | --- | --- | --- | --- |
| Q6P656-2 | CFAP161 | 119 | 119 | 182.7 | 179.3 | 1.525 | 0.0114 | 8.99533E-06 | 0.000338806 | up |
| Q8TE01 | derp12 | 94.5 | 92.6 | 138.2 | 136.6 | 1.47 | 0.0124 | 9.23297E-06 | 0.000343489 | up |
| Q8WUB8 | PHF10 | 169.9 | 169.8 | 130.9 | 129.4 | 0.765 | 0.0075 | 9.92183E-06 | 0.000364642 | down |
| Q8N3F8 | MICALL1 | 179.7 | 177.5 | 120 | 122.7 | 0.68 | 0.012 | 1.11336E-05 | 0.000404275 | down |
| O95863 | SNAI1 | 128.1 | 129.9 | 170.8 | 171.1 | 1.325 | 0.0097 | 1.14349E-05 | 0.000412744 | up |
| A6NHL2 | TUBAL3 | 199.8 | 193.7 | 104.7 | 101.7 | 0.525 | 0.0246 | 1.52982E-05 | 0.000539349 | down |
| Q9Y5V3-2 | MAGED1 | 4.6 | 4.5 | 7 | 7 | 1.54 | 0.015 | 1.6059E-05 | 0.000556466 | up |
| O43291 | SPINT2 | 170.8 | 169.2 | 131.1 | 128.9 | 0.7625 | 0.0126 | 1.82411E-05 | 0.000621427 | down |
| Q9H5K3 | POMK | 106.4 | 102.9 | 68.2 | 67.6 | 0.65 | 0.0178 | 2.45622E-05 | 0.000779819 | down |
| Q96K21 | ZFYVE19 | 181.4 | 181.8 | 119.8 | 117 | 0.65 | 0.0178 | 2.45622E-05 | 0.000783923 | down |
| Q96BP2 | CHCHD1 | 123.7 | 124.7 | 173.9 | 177.8 | 1.4175 | 0.0156 | 2.49679E-05 | 0.000788568 | up |
| A0A0B6XK00 | LGALS1 | 207.8 | 197.3 | 99.5 | 95.5 | 0.48 | 0.034 | 3.09551E-05 | 0.000911222 | down |
| Q8NA72 | POC5 | 175.7 | 180 | 123.5 | 120.8 | 0.6875 | 0.0183 | 3.23117E-05 | 0.000946562 | down |
| A0A024R8I8 | C9orf140 | 210.4 | 200.4 | 95.2 | 94 | 0.4625 | 0.0324 | 3.84372E-05 | 0.001074117 | down |
| A0A0A0MTS7 | TTN | 92 | 89.7 | 118.5 | 117.9 | 1.3 | 0.014 | 4.22181E-05 | 0.001148029 | up |
| P78562 | PHEX | 121.8 | 124.6 | 179.2 | 174.4 | 1.435 | 0.0201 | 4.7596E-05 | 0.001202592 | up |
| P52655 | GTF2A1 | 9.7 | 10 | 13.4 | 13.3 | 1.355 | 0.0176 | 5.32238E-05 | 0.001301407 | up |
| P27449 | ATP6V0C | 128.6 | 130.5 | 172.5 | 168.4 | 1.315 | 0.0158 | 5.31717E-05 | 0.001305397 | up |
| Q02446 | SP4 | 188.5 | 180.7 | 113.1 | 117.7 | 0.625 | 0.0333 | 9.75761E-05 | 0.001761017 | down |
| A8K9M5 | cDNA FLJ77947 mRNA | 128 | 131.6 | 169.1 | 171.2 | 1.31 | 0.0197 | 0.000106922 | 0.00182641 | up |
| P17029 | ZKSCAN1 | 170 | 176.3 | 125.5 | 128.2 | 0.7325 | 0.0233 | 0.000116627 | 0.001875927 | down |
| A0A024R035 | C9 | 95 | 99.1 | 134.5 | 134.8 | 1.39 | 0.0249 | 0.000128561 | 0.001901442 | up |
| Q9H1C4 | UNC93B1 | 129.5 | 131.4 | 166.8 | 172.4 | 1.3 | 0.0199 | 0.000119244 | 0.001907909 | up |
| Q460N5 | PARP14 | 195.4 | 184.9 | 112.2 | 107.5 | 0.5775 | 0.0433 | 0.000131856 | 0.001936015 | down |
| O60645 | EXOC3 | 167.5 | 174.3 | 129.3 | 128.8 | 0.755 | 0.0229 | 0.000158874 | 0.002160122 | down |
| Q6UWP8 | SBSN | 197.4 | 185.8 | 105.5 | 111.3 | 0.565 | 0.0511 | 0.000196016 | 0.002290248 | down |
| A0A0S2Z3C5 | BCL2L1 | 173.9 | 167.6 | 130.2 | 128.3 | 0.76 | 0.024 | 0.000183316 | 0.002301512 | down |
| Q86U70 | LDB1 | 182.1 | 190.7 | 109.8 | 117.5 | 0.6125 | 0.0488 | 0.000264167 | 0.002600501 | down |
| Q9UK99 | FBXO3 | 132 | 126.3 | 168.9 | 172.9 | 1.325 | 0.0292 | 0.000307351 | 0.002811126 | up |
| V9GY16 | TTLL12 | 166 | 173.3 | 129.9 | 130.7 | 0.7675 | 0.0269 | 0.000368177 | 0.003058392 | down |
| O43623 | SNAI2 | 124.4 | 120.6 | 182.8 | 172.2 | 1.45 | 0.041 | 0.000368976 | 0.003060835 | up |
| Q08AJ9 | HIST1H2AB | 125.8 | 132.4 | 172.5 | 169.3 | 1.325 | 0.0317 | 0.000392353 | 0.003122346 | up |
| Q96BQ1 | FAM3D | 176.1 | 165.7 | 129.6 | 128.7 | 0.7575 | 0.0347 | 0.000436166 | 0.003322756 | down |
| P60953 | CDC42 | 123 | 130.2 | 176.3 | 170.5 | 1.37 | 0.0377 | 0.000470437 | 0.003483188 | up |
| O00559-2 | EBAG9 | 122.5 | 130.7 | 173.6 | 173.1 | 1.37 | 0.0382 | 0.000487686 | 0.003529034 | up |
| Q49AM3 | TTC31 | 179.5 | 174.6 | 127.4 | 118.5 | 0.695 | 0.0447 | 0.000503647 | 0.003601554 | down |
| Q4KWH8 | PLCH1 | 188.7 | 173.7 | 118.1 | 119.5 | 0.6575 | 0.0487 | 0.00053197 | 0.003737969 | down |
| Q15555 | MAPRE2 | 17.5 | 18.5 | 23.6 | 24.3 | 1.3325 | 0.0359 | 0.000533045 | 0.003741184 | up |
| Q6ZN08 | ZNF66 | 202.3 | 178.9 | 108.9 | 109.9 | 0.575 | 0.0703 | 0.000568688 | 0.003793756 | down |
| O95644 | NFATC1 | 171.9 | 182 | 119.5 | 126.6 | 0.7 | 0.0467 | 0.000601894 | 0.003891135 | down |
| P46940 | IQGAP1 | 131 | 121.9 | 172.5 | 174.7 | 1.375 | 0.0422 | 0.000636248 | 0.003905068 | up |
| Q8WYL5 | SSH1 | 180.3 | 167.6 | 128.1 | 124 | 0.725 | 0.0429 | 0.000640781 | 0.003924943 | down |
| A0A024R435 | ZFAND2B | 124.7 | 121.6 | 184.4 | 169.4 | 1.4375 | 0.0522 | 0.000809884 | 0.004513915 | up |
| O75054-2 | IGSF3 | 124.7 | 132.8 | 168.2 | 174.4 | 1.3325 | 0.0417 | 0.000832886 | 0.004599836 | up |
| O75385 | ULK1 | 184.8 | 180.8 | 117.6 | 116.8 | 0.6425 | 0.0149 | 6.55614E-06 | 0.004969553 | down |
| P98173 | FAM3A | 126.1 | 134.7 | 170.2 | 169 | 1.3 | 0.0402 | 0.000980159 | 0.005146049 | up |
| B7Z2Q0 | cDNA FLJ57979 | 108.9 | 106.3 | 192.6 | 192.3 | 1.79 | 0.0129 | 6.09723E-06 | 0.005281941 | up |
| Q9UKI8-2 | TLK1 | 204.8 | 176.3 | 108.2 | 110.7 | 0.5775 | 0.0864 | 0.001036216 | 0.005284787 | down |
| B0QYD3 | APOBEC3B | 128.7 | 129.7 | 170 | 171.6 | 1.32 | 0.0062 | 5.2803E-06 | 0.005336619 | up |
| Q9HC85 | MB2 | 63.4 | 63.9 | 87.7 | 87 | 1.37 | 0.006 | 3.8492E-06 | 0.005835384 | up |
| Q16531-2 | DDB1 | 75 | 75.7 | 48.9 | 48.6 | 0.6475 | 0.0077 | 4.89685E-06 | 0.005938898 | down |
| Q9H1X3 | DNAJC25 | 126.7 | 133.9 | 164.5 | 174.9 | 1.305 | 0.047 | 0.001497757 | 0.006199589 | up |
| Q9UJU1 | VIL2 | 36.8 | 34 | 47 | 50 | 1.3725 | 0.0568 | 0.001565606 | 0.006384555 | up |
| A0A024R1V5 | MLLT6 | 175.3 | 178.4 | 129.9 | 116.3 | 0.695 | 0.067 | 0.001651718 | 0.006606873 | down |
| Q8IYL3 | C1orf174 | 204.2 | 200.6 | 96.1 | 99.1 | 0.4825 | 0.0198 | 3.42975E-06 | 0.006932672 | down |
| Q6ZUY8 | Lipase | 180 | 165.7 | 130.5 | 123.8 | 0.7375 | 0.0579 | 0.001801993 | 0.006933559 | down |
| Q6UVK1 | CSPG4 | 121.6 | 134 | 174.2 | 170.2 | 1.35 | 0.0571 | 0.001862421 | 0.007098504 | up |
| Q5LJA5 | UCHL5 | 123.7 | 135.4 | 169.8 | 171 | 1.315 | 0.0529 | 0.001945072 | 0.007249486 | up |
| P78386 | KRT85 | 123.1 | 135.2 | 172 | 169.7 | 1.3275 | 0.0548 | 0.001953285 | 0.007266701 | up |
| P09914 | IFIT1 | 123.1 | 135 | 172 | 169.9 | 1.3275 | 0.0548 | 0.001953285 | 0.007271162 | up |
| Q15326 | ZMYND11 | 124.1 | 136.1 | 168.9 | 171 | 1.31 | 0.0536 | 0.002113497 | 0.007525687 | up |
| Q6ZMI0 | PPP1R21 | 164.3 | 176.7 | 132.8 | 126.2 | 0.76 | 0.0548 | 0.002101047 | 0.00762919 | down |
| A0A0U1RRE5 | NBDY | 182.5 | 165.8 | 122.4 | 129.4 | 0.725 | 0.0642 | 0.002100616 | 0.007632195 | down |
| A0A087X1Z1 | TUBGCP5 | 26.2 | 28 | 20.9 | 19.4 | 0.745 | 0.0605 | 0.002291089 | 0.007998369 | down |
| P0CJ79 | ZNF888 | 80.2 | 71.8 | 103.2 | 103.1 | 1.365 | 0.0634 | 0.002315865 | 0.008024802 | up |
| Q9BXP2 | SLC12A9 | 165.7 | 173.3 | 125 | 136 | 0.7675 | 0.0557 | 0.002417527 | 0.008254441 | down |
| J3KR35 | CCDC12 | 43.8 | 44.2 | 126.3 | 125.1 | 2.8575 | 0.0072 | 1.36174E-06 | 0.008257605 | up |
| Q9H0U9 | TSPYL1 | 121.6 | 135.1 | 170.6 | 172.8 | 1.34 | 0.0609 | 0.002434897 | 0.008295066 | up |
| A0A024RCE4 | RASSF7 | 122.7 | 135.4 | 168.4 | 173.5 | 1.325 | 0.0593 | 0.00251927 | 0.008534556 | up |
| Q12986 | NFX1 | 179 | 181.2 | 110.9 | 128.9 | 0.665 | 0.0873 | 0.002552239 | 0.008579144 | down |
| P35052 | GPC1 | 186.4 | 164.8 | 125.3 | 123.5 | 0.71 | 0.0736 | 0.002595585 | 0.00868633 | down |
| C9JJN9 | VPS8 | 108 | 127.4 | 179.5 | 185.1 | 1.5575 | 0.096 | 0.002767282 | 0.009085436 | up |
| Q9NZ23 | YA61 | 80.5 | 80.2 | 106.4 | 105.4 | 1.3175 | 0.0073 | 3.05389E-06 | 0.009259406 | up |
| P30501 | HLA-C | 120.1 | 133.6 | 168.5 | 177.8 | 1.3675 | 0.069 | 0.002871659 | 0.009302213 | up |
| Q9HAT2 | SIAE | 183.8 | 163.5 | 128.4 | 124.4 | 0.7325 | 0.0699 | 0.002930961 | 0.00945894 | down |
| P33552 | CKS2 | 100.3 | 122.8 | 193.3 | 183.6 | 1.7075 | 0.1202 | 0.003097913 | 0.009698371 | up |
| Q9NS00 | C1GALT1 | 110.7 | 129.7 | 175.9 | 183.7 | 1.5075 | 0.0934 | 0.003191851 | 0.009701946 | up |
| Q9Y3Q8 | TSC22D4 | 23 | 25.5 | 31.3 | 32.9 | 1.3275 | 0.0652 | 0.003257569 | 0.009822925 | up |
| P38398-7 | BRCA1 | 184.6 | 167.3 | 117.8 | 130.3 | 0.7075 | 0.0811 | 0.003298091 | 0.009920448 | down |
| Q9HBM1 | SPC25 | 121.5 | 135.3 | 168.3 | 174.8 | 1.34 | 0.0681 | 0.003389355 | 0.009977208 | up |
| Q8NCN4 | RNF169 | 117.6 | 133.1 | 170 | 179.4 | 1.4025 | 0.0784 | 0.003346462 | 0.01001132 | up |
| Q9H2P9-5 | DPH5 | 124 | 137.4 | 165.9 | 172.7 | 1.3 | 0.0619 | 0.00351099 | 0.010123939 | up |
| A1A5C4 | RRBP1 | 118.3 | 132.6 | 168.6 | 180.6 | 1.3975 | 0.0787 | 0.003489576 | 0.010222603 | up |
| Q99638 | RAD9A | 113.2 | 130.9 | 171.7 | 184.2 | 1.4675 | 0.0941 | 0.003989919 | 0.010977708 | up |
| P02774-3 | GC | 120.3 | 137 | 170.8 | 172 | 1.34 | 0.0734 | 0.00420964 | 0.011467771 | up |
| Q3MIH3 | UBA52 | 113.4 | 131.1 | 184.8 | 170.7 | 1.4625 | 0.0963 | 0.004376268 | 0.011826065 | up |
| A0A024RC24 | IMPACT | 118.4 | 133.3 | 166.9 | 181.4 | 1.3875 | 0.0837 | 0.004466428 | 0.012021491 | up |
| Q9UL54-2 | TAOK2 | 115.5 | 135.1 | 173.8 | 175.6 | 1.4025 | 0.0888 | 0.004829288 | 0.012699394 | up |
| Q9Y2Z0-2 | SUGT1 | 159.8 | 181.5 | 129.3 | 129.5 | 0.76 | 0.076 | 0.005399266 | 0.013563027 | down |
| Q96QD5 | DEPDC7 | 161 | 182.7 | 124.5 | 131.9 | 0.7475 | 0.0813 | 0.005461251 | 0.013628406 | down |
| O15121 | DEGS1 | 122.8 | 138.5 | 165.9 | 172.8 | 1.3025 | 0.0729 | 0.005543029 | 0.01378709 | up |
| Q9ULR5 | PAIP2B | 102.2 | 124.4 | 173 | 200.4 | 1.6625 | 0.1417 | 0.005819512 | 0.014235384 | up |
| B3KQT6 | cDNA PSEC0169 fis | 21.5 | 24.6 | 29.7 | 30.8 | 1.3175 | 0.0792 | 0.006243896 | 0.014854055 | up |
| A0A024R0L2 | MGC20255 | 119.7 | 136 | 164.6 | 179.7 | 1.3525 | 0.0894 | 0.006856532 | 0.01582116 | up |
| Q6ZN64 | NEK3 | 112.7 | 133.4 | 169.3 | 184.6 | 1.4475 | 0.1098 | 0.006918563 | 0.015946092 | up |
| Q9UPU7 | TBC1D2B | 121 | 138.2 | 166.4 | 174.4 | 1.32 | 0.083 | 0.007046103 | 0.016196955 | up |
| Q9BWT6 | MND1 | 53.2 | 61.2 | 73.5 | 76.6 | 1.3175 | 0.0846 | 0.007563856 | 0.017159455 | up |
| B2RDL4 | cDNA, FLJ96668 | 160 | 184 | 122.9 | 133 | 0.7475 | 0.0916 | 0.007655368 | 0.017315236 | down |
| E9PLY5 | MACF1 | 121 | 139.9 | 169 | 170.1 | 1.31 | 0.0839 | 0.007844788 | 0.017618812 | up |
| Q0VAN5 | TBCEL | 118.6 | 138.9 | 169.3 | 173.1 | 1.34 | 0.0914 | 0.00800482 | 0.017925121 | up |
| O00273-2 | DFFA | 112.1 | 135.1 | 170.9 | 181.8 | 1.4375 | 0.113 | 0.008035936 | 0.017968259 | up |
| Q5U0N1 | MLF2 | 105.8 | 132.5 | 175 | 186.7 | 1.535 | 0.1334 | 0.00806614 | 0.018029147 | up |
| Q9C0H5-2 | ARHGAP39 | 28.3 | 34.9 | 45.5 | 47 | 1.48 | 0.1225 | 0.008147082 | 0.018169882 | up |
| B2RE40 | cDNA, FLJ96911 | 104.7 | 132.1 | 175.2 | 188 | 1.555 | 0.14 | 0.008479514 | 0.018657392 | up |
| A0A024R1E6 | PRAME | 115.8 | 138.4 | 175.4 | 170.3 | 1.37 | 0.1025 | 0.009080041 | 0.019650738 | up |
| A0A024R2V1 | hCG_20123 | 119.6 | 140.3 | 167.9 | 172.2 | 1.3175 | 0.0912 | 0.009394445 | 0.02023727 | up |
| O75648 | TRMU | 120.6 | 138.6 | 164.1 | 176.6 | 1.3175 | 0.0912 | 0.00939977 | 0.020241549 | up |
| Q96KF7 | SMIM8 | 111.8 | 129.1 | 162.9 | 196.1 | 1.4975 | 0.1347 | 0.009835937 | 0.020876836 | up |
| P27815 | PDE4A | 109.2 | 136.2 | 178.4 | 176.1 | 1.46 | 0.1268 | 0.00995812 | 0.021069798 | up |
| B1AQP5 | DEDD | 104 | 134 | 177.1 | 184.9 | 1.545 | 0.1481 | 0.010525952 | 0.021866864 | up |
| A0A0A6YYJ5 | MACF1 | 109.9 | 137.1 | 172.6 | 180.4 | 1.4475 | 0.1283 | 0.010998439 | 0.022623655 | up |
| B2R4A5 | cDNA, FLJ92019 | 116.6 | 139.7 | 167.6 | 176.1 | 1.3525 | 0.1082 | 0.01192158 | 0.023922059 | up |
| P14373 | TRIM27 | 116.7 | 140.1 | 167.8 | 175.4 | 1.3475 | 0.1076 | 0.012139726 | 0.024311525 | up |
| Q9UK58 | CCNL1 | 98.5 | 131.7 | 174.9 | 194.8 | 1.6425 | 0.1781 | 0.012396163 | 0.02468648 | up |
| Q6UB28 | METAP1D | 103.2 | 132.1 | 169.3 | 195.5 | 1.5725 | 0.1639 | 0.012580649 | 0.025004607 | up |
| Q8NFZ8 | CADM4 | 14.5 | 18.7 | 24.2 | 25.1 | 1.5075 | 0.149 | 0.012650146 | 0.025126265 | up |
| Q8WZA1-2 | POMGNT1 | 18.7 | 22.6 | 28.9 | 27.1 | 1.37 | 0.116 | 0.012906913 | 0.025206931 | up |
| Q9H7E2-3 | TDRD3 | 32.1 | 41.2 | 52.2 | 56.5 | 1.5075 | 0.1504 | 0.012931646 | 0.025247103 | up |
| P78345 | RPP38 | 119.8 | 141.4 | 165.4 | 173.5 | 1.3075 | 0.0992 | 0.012970931 | 0.025299366 | up |
| Q7L8L6 | FASTKD5 | 155.3 | 187.6 | 129.1 | 128 | 0.755 | 0.1073 | 0.013097066 | 0.025463484 | down |
| Q6YHU6 | THADA | 121.2 | 125.9 | 156.4 | 196.5 | 1.4275 | 0.1333 | 0.013701385 | 0.025947908 | up |
| A0A024R5P6 | C1orf121 | 118.4 | 139.8 | 163 | 178.7 | 1.335 | 0.1085 | 0.013524767 | 0.025970293 | up |
| A0A0S2Z319 | BAK1 | 139.1 | 121.9 | 160.1 | 178.9 | 1.305 | 0.1004 | 0.013626625 | 0.026132781 | up |
| O94964-2 | SOGA1 | 119.6 | 141.6 | 165.4 | 173.4 | 1.305 | 0.101 | 0.013865391 | 0.026184905 | up |
| P40337 | VHL | 110.6 | 137.6 | 167.7 | 184.2 | 1.4375 | 0.1377 | 0.014180515 | 0.026409902 | up |
| A8K6L3 | cDNA FLJ76883 | 115.7 | 141 | 168.6 | 174.8 | 1.3525 | 0.1147 | 0.014108688 | 0.026594679 | up |
| A0A024R5L8 | STARD10 | 27.3 | 33.1 | 39.5 | 43.8 | 1.39 | 0.1264 | 0.014587051 | 0.026821067 | up |
| P17535 | JUND | 115.5 | 140.5 | 167.7 | 176.3 | 1.355 | 0.1189 | 0.015311025 | 0.027756668 | up |
| Q9UJ41 | RABGEF1 | 22.9 | 27.6 | 32.3 | 34.8 | 1.34 | 0.1161 | 0.015854759 | 0.028269114 | up |
| Q8N1G1 | REXO1 | 117.5 | 142.4 | 171.8 | 168.3 | 1.32 | 0.1101 | 0.015886925 | 0.028309818 | up |
| Q96AQ6 | PBXIP1 | 27.6 | 33 | 38.3 | 40.8 | 1.3175 | 0.1096 | 0.015926676 | 0.028355656 | up |
| B2R9K5 | cDNA, FLJ94436 | 111.1 | 140.2 | 171.1 | 177.6 | 1.4075 | 0.1352 | 0.015983283 | 0.028439738 | up |
| Q6ZUT1-2 | NKAPD1 | 119.5 | 140.8 | 162.3 | 177.5 | 1.315 | 0.1101 | 0.016401628 | 0.028912638 | up |
| O00488 | ZNF593 | 113.8 | 139.2 | 164.7 | 182.3 | 1.385 | 0.1306 | 0.01651768 | 0.029058082 | up |
| A0A024R8Y1 | POGK | 117.1 | 142.5 | 169.3 | 171.1 | 1.325 | 0.1134 | 0.016607649 | 0.029199415 | up |
| K7EPJ5 | MGRN1 | 113.7 | 141.1 | 166.1 | 179.1 | 1.3725 | 0.1318 | 0.018265603 | 0.031646462 | up |
| P41229 | KDM5C | 119.1 | 142.7 | 163.7 | 174.5 | 1.3025 | 0.1109 | 0.018397085 | 0.031846967 | up |
| A0A024R9R3 | VPS18 | 33.2 | 40.3 | 46.9 | 50 | 1.33 | 0.1195 | 0.018505472 | 0.031988935 | up |
| Q5JUR7 | TEX30 | 113.8 | 141.8 | 166.4 | 178 | 1.3625 | 0.1314 | 0.019436893 | 0.033220214 | up |
| B7ZKJ5 | ZFX | 112.8 | 142.1 | 169.1 | 176 | 1.3725 | 0.1345 | 0.019473557 | 0.0332735 | up |
| B4DZS0 | DFFB | 14.2 | 19.1 | 23.6 | 25.8 | 1.5175 | 0.1771 | 0.019840124 | 0.033785598 | up |
| Q9BUU9 | TUBB | 107.9 | 140.2 | 170 | 181.9 | 1.445 | 0.157 | 0.019972176 | 0.033991377 | up |
| D3VVG8 | ATXN3 | 116.4 | 141.8 | 162.6 | 179.2 | 1.3375 | 0.1267 | 0.020566397 | 0.034826761 | up |
| Q9UG01 | IFT172 | 109.7 | 141.7 | 171.3 | 177.2 | 1.41 | 0.1489 | 0.020710598 | 0.035041592 | up |
| A0A024RC97 | PTDSS2 | 98.9 | 138.2 | 175.3 | 187.6 | 1.575 | 0.195 | 0.020782831 | 0.035144196 | up |
| A8K6I6 | cDNA FLJ75092 | 113 | 140.1 | 162.3 | 184.6 | 1.3875 | 0.1428 | 0.020961194 | 0.035376756 | up |
| Q8WUH1-2 | CHURC1 | 112.1 | 142.3 | 171.4 | 174.2 | 1.375 | 0.1388 | 0.020974304 | 0.035389031 | up |
| Q9UJP4 | KLHL21 | 114.5 | 142.7 | 166.2 | 176.6 | 1.3475 | 0.1316 | 0.021569205 | 0.03612142 | up |
| Q8N729 | NPW | 117.3 | 142 | 161.5 | 179.2 | 1.3275 | 0.1256 | 0.021605426 | 0.036172088 | up |
| Q86TB9 | PATL1 | 106.2 | 141 | 170.1 | 182.6 | 1.4575 | 0.1659 | 0.021842139 | 0.036477756 | up |
| A0A0A0MSZ4 | FDXR | 113 | 143.7 | 169.9 | 173.4 | 1.355 | 0.1369 | 0.022969781 | 0.038182224 | up |
| Q96LZ7 | RMDN2 | 105.3 | 138.2 | 164.4 | 192.1 | 1.49 | 0.1792 | 0.023099951 | 0.038189233 | up |
| P10636-7 | MAPT | 117.8 | 141.4 | 159.7 | 181.1 | 1.3275 | 0.1286 | 0.023093788 | 0.038189455 | up |
| Q9ULV0 | MYO5B | 106.8 | 141.3 | 168.1 | 183.8 | 1.445 | 0.1682 | 0.02416294 | 0.039676162 | up |
| Q8TDI0 | CHD5 | 110.8 | 142.7 | 167.4 | 179.2 | 1.39 | 0.1513 | 0.024345535 | 0.039932737 | up |
| Q96S66 | CLCC1 | 114.1 | 142.7 | 163.1 | 180.1 | 1.3525 | 0.1425 | 0.025939411 | 0.041778643 | up |
| A0A024R3H2 | SORL1 | 115.8 | 144.2 | 163.2 | 176.8 | 1.325 | 0.1352 | 0.027184472 | 0.043529611 | up |
| E9PR47 | AAMDC | 23.8 | 30.1 | 35.3 | 35.2 | 1.325 | 0.1351 | 0.027232057 | 0.043594296 | up |
| A0A024R1Y7 | LGP1 | 56.4 | 69.6 | 78.2 | 85.6 | 1.315 | 0.1338 | 0.028425711 | 0.045135772 | up |
| O60663-3 | LMX1B | 197.6 | 150.7 | 122.2 | 129.5 | 0.7375 | 0.1567 | 0.028339196 | 0.045175837 | down |
| H0YIV9 | Uncharacterized protein (Fragment) | 105.2 | 141.6 | 165 | 188.2 | 1.465 | 0.1856 | 0.028770502 | 0.045587751 | up |
| Q8NB37 | GATD1 | 23.8 | 31.1 | 36.3 | 37.7 | 1.3725 | 0.1547 | 0.028890073 | 0.045753304 | up |
| Q9Y6R0 | NUMBL | 19 | 24.7 | 29 | 29.6 | 1.365 | 0.1528 | 0.029270353 | 0.046138658 | up |
| Q6NUK7 | LYN | 96.2 | 138.8 | 166.7 | 198.3 | 1.605 | 0.2324 | 0.030511823 | 0.047649676 | up |
| G5E9I4 | BRMS1 | 114.3 | 146 | 166.7 | 173.1 | 1.325 | 0.1411 | 0.030744794 | 0.047988785 | up |
| P13631 | RARG | 105.4 | 141.8 | 164.9 | 187.8 | 1.455 | 0.1869 | 0.030802116 | 0.048053519 | up |
| Q13751 | LAMB3 | 108.8 | 145.5 | 171.3 | 174.5 | 1.3875 | 0.1646 | 0.031433372 | 0.048724941 | up |
| P52815 | MRPL12 | 114 | 145.5 | 164.7 | 175.8 | 1.33 | 0.1444 | 0.0314544 | 0.048745076 | up |
| P08138 | NGFR | 98 | 141.2 | 167.4 | 193.4 | 1.56 | 0.2232 | 0.032261034 | 0.049880395 | up |

**Table S5. Summary of the 50 Colorectal Cancer GWAS Risk Alleles evaluated in our study.**

| **Region** | **Chr.** | **Physical Position** | **Strongest Risk Allele** | **SNP-Proxy** | ***P-value*** | **OR (95% CI)** |  |
| --- | --- | --- | --- | --- | --- | --- | --- |
| 9q22.32 | 9 | 96631134 | rs10114408 | rs7869842 | 9.00E-07 | 1.13(1.08-1.19) |  |
| 19q13.11 | 19 | 33532300 | rs10411210 | rs3786934 | 8.00E-28 | 1.2(1.16-1.24) |  |
| 8q24.21 | 8 | 128407443 | rs10505477 | rs10505477 | 1.00E-09 | 1.06(1.03-1.09) |  |
| 12p13.32 | 12 | 4368352 | rs10774214 | rs10774214 | 4.00E-06 | 1.27(1.14-1.40) |  |
| 10p14 | 10 | 8701219 | rs10795668 | rs827401 | 2.00E-07 | 1.14(1.08-1.19) |  |
| 12q21.1 | 12 | 72414563 | rs10879357 | rs4488237 | 8.00E-07 | 1.18(1.11-1.27) |  |
| 1q25.3 | 1 | 183081194 | rs10911251 | rs10797838 | 1.00E-07 | 1.14(1.08-1.18) |  |
| 3q26.2 | 3 | 169492101 | rs10936599 | rs12696304 | 8.00E-09 | 1.28(1.18-1.39) |  |
| 12q13.12 | 12 | 51155663 | rs11169552 | rs11169544 | 2.00E-08 | 1.18(1.11-1.25) |  |
| 15q13.3 | 15 | 33004247 | rs11632715 | rs11638007 | 6.00E-10 | 1.11(1.08-1.15) |  |
| 2q32.3 | 2 | 192587204 | rs11903757 | rs12475675 | 3.00E-13 | 1.12(1.10-1.16) |  |
| 8p12 | 8 | 29344462 | rs12548021 | rs12548021 | 2.00E-07 | 1.14(1.06-1.19) |  |
| 4q22.2 | 4 | 94887031 | rs13130787 | rs11934535 | 7.00E-10 | 1.07(1.04-1.10) |  |
| 6p21.2 | 6 | 36622900 | rs1321311 | rs1321310 | 1.00E-14 | 1.27(1.16-1.39) |  |
| 10q25.3 | 10 | 118487100 | rs1665650 | rs1665650 | 3.00E-11 | 1.17(1.12-1.23) |  |
| 8q23.3 | 8 | 117630683 | rs16892766 | rs16892766 | 4.00E-08 | 1.35(1.20-1.49) |  |
| 15q13.3 | 15 | 32993111 | rs16969681 | rs16969344 | 3.00E-06 | 1.47(1.25-1.72) |  |
| 14q23.1 | 14 | 59189361 | rs17094983 | rs710005 | 4.00E-07 | 1.24(1.14-1.34) |  |
| 4q13.2 | 4 | 67357454 | rs17730929 | rs17087196 | 2.00E-10 | 1.12(1.08-1.16) |  |
| 1q23.3 | 1 | 162821291 | rs1912453 | rs1892124 | 1.00E-08 | 1.1(1.06-1.12) |  |
| 14q22.2 | 14 | 54560018 | rs1957636 | rs713424 | 9.00E-26 | 1.19(1.15-1.23) |  |
| 6q22.1 | 6 | 117819357 | rs2057314 | rs6911915 | 7.00E-11 | 1.24(1.17-1.33) |  |
| 8q24.21 | 8 | 130820039 | rs2128382 | rs2894493 | 3.00E-06 | 1.37(1.20-1.56) |  |
| 20p12.3 | 20 | 7812350 | rs2423279 | rs6086235 | 9.00E-07 | 1.13(1.08-1.19) |  |
| 8q22.1 | 8 | 96595736 | rs3104964 | rs3104964 | 2.00E-10 | 1.12(1.09-1.16) |  |
| 12p13.32 | 12 | 4388271 | rs3217810 | rs3217810 | 4.00E-10 | 1.17(1.11-1.22) |  |
| 5q21.3 | 5 | 108948937 | rs367615 | rs367615 | 3.00E-06 | 1.13(1.08-1.20) |  |
| 11q23.1 | 11 | 111171709 | rs3802842 | rs3802840 | 2.00E-10 | 1.12(1.18-1.16) |  |
| 11q13.4 | 11 | 74345550 | rs3824999 | rs10899024 | 6.00E-06 | 1.11(1.06-1.15) |  |
| 7p15.3 | 7 | 25133849 | rs39453 | rs39454 | 3.00E-18 | 1.27(1.20-1.34) |  |
| 14q22.2 | 14 | 54410919 | rs4444235 | rs2071047 | 1.00E-10 | 1.11(1.08-1.15) |  |
| 3p24.3 | 3 | 23143047 | rs4591517 | rs11713229 | 1.00E-10 | 1.1(1.07-1.13) |  |
| 15q13.3 | 15 | 32994756 | rs4779584 | rs4779584 | 2.00E-10 | 1.09(1.05-1.11) |  |
| 20p12.3 | 20 | 6699595 | rs4813802 | rs4813802 | 8.00E-07 | 1.18(1.11-1.27) |  |
| 20q13.33 | 20 | 60921044 | rs4925386 | rs4925386 | 2.00E-06 | 1.28(1.16-1.43) |  |
| 18q21.1 | 18 | 46453463 | rs4939827 | rs7226855 | 3.00E-06 | 1.06(0.88-1.29) |  |
| 12q24.21 | 12 | 115116352 | rs59336 | rs59336 | 3.00E-08 | 1.09(1.06-1.13) |  |
| Xp22.2 | 23 | 9751474 | rs5934683 | rs5934683 | 1.00E-11 | 1.13(1.09-1.18) |  |
| 5q31.1 | 5 | 134499092 | rs647161 | rs12657484 | 5.00E-07 | 1.12(1.08-1.19) |  |
| 1q41 | 1 | 222164948 | rs6687758 | rs6695584 | 7.00E-06 | 1.1(1.05-1.14) |  |
| 1q41 | 1 | 222045446 | rs6691170 | rs11579490 | 4.00E-08 | 1.16(1.10-1.22) |  |
| 8q24.21 | 8 | 128413305 | rs6983267 | rs6983267 | 9.00E-06 | 1.07(1.04-1.11) |  |
| 8q24.21 | 8 | 128424792 | rs7014346 | rs7014346 | 3.00E-07 | 1.09(1.06-1.13) |  |
| 15q13.1 | 15 | 28705281 | rs7163702 | - | 8.00E-06 | 1.11(1.06-1.16) |  |
| 12q24.21 | 12 | 115891403 | rs7315438 | rs7315438 | 5.00E-08 | 1.18(1.11-1.25) |  |
| 6q25.3 | 6 | 160840252 | rs7758229 | rs3123636 | 2.00E-08 | 1.18(1.11-1.24) |  |
| 3p21.31 | 3 | 47388947 | rs8180040 | rs8180040 | 4.00E-07 | 1.14(1.08-1.20) |  |
| 6q25.3 | 6 | 158435572 | rs9365723 | rs1998791 | 3.00E-08 | 1.04(1.04-1.10) |  |
| 20p12.3 | 20 | 6404281 | rs961253 | rs5005940 | 8.00E-10 | 1.11(1.08-1.15) |  |
| 16q22.1 | 16 | 68820946 | rs9929218 | rs9925923 | 2.00E-06 | 1.28(1.15-1.41) |  |

**Supplementary methods**

**1. Association analysis and expression Quantitative Trait Loci (eQTL) analysis**

The expression data of the gene was adjusted for somatic copy-number effects and CpG methylation status using a multivariate linear model. Cis-eQTL analysis between 656,857 SNP loci and corresponding mRNA transcripts was performed. After excluded with MAF < 0.05, absent calls >90%, false discovery rate (FDR) > 0.1 and matched with NHGRI GWAS database, 18 SNPs were present in TCGA germline genotype. SNAPinfo software was selected to obtain pairwise linkage disequilibrium between SNPs. In cis-eQTL analysis, the range was set within ± 1Mb regions between SNP locus and transcripts. And each target gene was overlapped using ENCODE DNaseI hypersensitivity data from the HCT116 cell line and then analyzed for transcription factor DNA binding motif enrichment. A hypergeometric distribution test was used for overlap analyses with a significance level of *P* < 0.05. TFs that satisfied the above criteria were considered as candidates for trans-acting risk SNPs.

**2. Cell culture**

HCT116, T84, SW480, RKO, and CCD-18Co cell lines were cultured in DMEM/high glucose medium (Hyclone, Cat.SH30022.01B, USA), LOVO cell line was F-12K medium (Gibco, Cat.21127022, USA). Each medium was supplemented with 10% fetal bovine serum (Hyclone, Cat.SH30087.01, USA) and 1% penicillin-streptomycin (Hyclone, Cat.SH30010, USA). Cells were cultured at 37℃ containing 5% CO_2_.

**3. Antibodies information**

| Gene | Manufacturer | Cat. | Dilution for WB | Dilution for IHC |
| --- | --- | --- | --- | --- |
| CCDC12 | Ptgcn | 25138-1-AP | 1:500 |  |
| CCDC12 | Abcam | ab203656 |  | 1:100 |
| Snail | CST | 3879 | 1:300 |  |
| N-CAD | CST | 13116s | 1:1000 |  |
| VIM | CST | 5741s | 1:1000 |  |
| FN | Abcam | ab2413 | 1:1000 |  |
| E-CAD | CST | 3195s | 1:1000 |  |
| GAPDH | Abcam | ab8245 | 1:5000 |  |
| β-actin | Abcam | Ab8226 | 1:5000 |  |

**4. Primer sequences**

a. 18s rRNA

forward 5’-CCTGGATACCGCAGCTAGGA-3’

reverse 5’-GCGGCGCAATACGAATGCCCC-3’

b. CCDC12

forward 5’-CTGACTGGGACCTCAAGAGA-3’

reverse 5’-CCTTTCAGCCTTTCACGGAT-3’

c. Snail

forward 5’-GAGGCGGTGGCAGACTAGAGT-3’

reverse 5’-CGGGCCCCCAGAATAGTTC-3’
